# Extracellular vesicle-based therapies in IBD: immunomodulation, regeneration, and translation

**DOI:** 10.3389/fimmu.2026.1943448

**Published:** 2026-09-07

**Authors:** Enhui Lu, Minli Sun, Hai Meng, Miao Zhang

**Affiliations:** 1 Department of Gastroenterology, Binhai County People’s Hospital, Yancheng, China; 2 Department of Geriatrics, Binhai County People’s Hospital, Yancheng, China

**Keywords:** engineered extracellular vesicles, extracellular vesicles, immunomodulation, inflammatory bowel disease, innate immunity, mucosal regeneration, plant-derived EV-like nanoparticles

## Abstract

Inflammatory bowel disease (IBD) is sustained by dysregulated mucosal immune activation, maladaptive immune-cell crosstalk, epithelial barrier failure, and impaired tissue repair. This narrative review critically evaluates unmodified native mammalian EVs, donor-conditioned EVs, milk-derived EVs, post-isolation engineered EV products, and plant-derived EV-like nanoparticles (PELNs) as candidate immunomodulatory and mucosal-repair platforms. The evidence is dominated by chemically induced rodent colitis, particularly acute mucosal injury models; controlled efficacy data in patients with luminal ulcerative colitis or Crohn’s disease are not available, and the small uncontrolled perianal-fistula study provides only preliminary local safety and feasibility observations. Reported outcomes include changes in inflammatory myeloid states, tolerance-associated dendritic-cell states, Treg/Th17 balance, epithelial protection, barrier restoration, tissue repair, and, for a narrower subset of preparations, stem/progenitor-associated regeneration. Mechanistic intervention studies include dendritic-cell AMPK dependence for broccoli-derived nanoparticles and PD-1/PHB1-related activity for engineered PD-L1/miR-27a-3p EVs, whereas many macrophage, microbiota, barrier, and cargo-delivery claims remain associative or preparation-specific. Engineering may improve exposure, target-cell engagement, cargo control, or receptor signaling, but each proposed advantage requires direct comparison with an appropriate unmodified or clinically relevant comparator and introduces additional manufacturing and safety requirements. We therefore separate product identity, disease-relevant regional or systemic exposure, target-cell engagement, functional intracellular cargo delivery, surface-receptor signaling, pharmacodynamic response, and repair outcomes. Translation will require reproducible dose metrics, mechanism-linked validation, validated lot-release potency assays, chronic repeat-dose safety, and phenotype-relevant human models.

## 1 Introduction

Inflammatory bowel disease (IBD), principally comprising ulcerative colitis (UC) and Crohn’s disease (CD), is a chronic, relapsing, immune-mediated disorder of the gastrointestinal tract with a rising global burden (1). UC typically involves continuous mucosal inflammation of the colon and rectum, whereas CD may affect any gastrointestinal segment and can produce transmural inflammation, strictures, fistulas, and progressive bowel damage (2). These anatomical and biological differences are relevant to therapeutic development because the accessibility, cellular composition, and regenerative capacity of the affected intestinal compartment vary across disease phenotypes.

The therapeutic landscape of IBD has expanded through biologics and small molecules targeting inflammatory cytokines, lymphocyte trafficking, and intracellular signaling. Nevertheless, primary non-response, secondary loss of response, incomplete mucosal healing, and treatment-limiting adverse effects remain important clinical challenges. Current treat-to-target strategies therefore extend beyond symptom control toward objective suppression of inflammation, restoration of mucosal integrity, and prevention of progressive bowel damage (3). Achieving these goals requires more than inhibition of an individual cytokine. Intestinal inflammation is sustained by reciprocal interactions among epithelial barrier disruption, microbial translocation, innate immune activation, antigen presentation, maladaptive lymphocyte responses, and defective tissue repair (4).

At the mucosal interface, macrophages, dendritic cells, epithelial cells, and lymphocytes form interconnected inflammatory and regenerative circuits. Activated myeloid-cell states can amplify cytokine production and tissue injury, whereas tolerogenic dendritic-cell programs, regulatory T-cell responses, and inflammation-resolving macrophage states may support immune restraint and epithelial restitution. Barrier repair is likewise not a passive consequence of reduced inflammation: it requires epithelial survival, tight-junction restoration, crypt-cell renewal, and coordinated regenerative signaling. Therapeutic platforms capable of influencing several of these processes may therefore provide advantages over interventions that suppress inflammation without directly supporting mucosal repair.

Extracellular vesicles (EVs) have attracted attention as candidate regulators of these immune-regenerative networks. EVs are lipid bilayer-enclosed particles that transport proteins, lipids, nucleic acids, metabolites, and surface-associated signals between cells. Their biological activity may involve cargo transfer, receptor engagement, protection of labile molecules, or modification of recipient-cell signaling. In experimental colitis, EV-based interventions have been associated with reductions in disease activity, histological injury, myeloperoxidase activity, and pro-inflammatory mediators, together with increases in anti-inflammatory and tissue-protective responses (5). However, these findings mainly provide preclinical proof-of-concept. They do not establish a shared mechanism across EV sources, and clinical evidence for EV-based treatment of luminal UC or CD remains extremely limited.

Native EV preparations illustrate both the therapeutic potential and the mechanistic limitations of the field. Mesenchymal stromal/stem cell-derived EVs, milk-derived EVs, and other native vesicle preparations have been linked to macrophage-state regulation, T-cell responses, epithelial protection, and microbiota-related changes. Yet their active components, target-cell preferences, dose metrics, and potency determinants are often incompletely defined. Fluorescent accumulation or cellular uptake further demonstrates exposure rather than necessarily proving intact-vesicle delivery, functional cargo activity, receptor-dependent signaling, or causal pathway engagement. EV-based therapy should therefore be evaluated as an immunomodulatory and regenerative strategy, not simply as a nanoscale delivery system.

Engineering provides one approach to increasing mechanistic and product control. Surface ligands, defined cargo loading, immune-checkpoint display, donor-cell programming, hybrid membranes, and biomaterial-assisted retention may improve intestinal exposure, cell-specific engagement, or regulation of selected immune pathways. For example, engineered PD-L1/miR-27a-3p EVs showed preferential localization to inflamed intestine and regulation of PD-1- and PHB1-related signaling supported by pathway intervention, accompanied by reduced Th17 polarization, increased FOXP3-positive regulatory T cells, and epithelial protection in preclinical and patient-derived experimental systems (6). This study supports a programmable immune-regulatory design principle, although the evidence remains preparation-specific and preclinical.

Plant-derived EV-like nanoparticles (PELNs) represent a distinct but related strategy, particularly for oral intestinal intervention. Because their biogenesis and marker systems are not equivalent to those of mammalian EVs, they should be described as operationally isolated vesicle-like nanoparticles rather than assumed to be plant exosomes. Selected preparations have nevertheless demonstrated gastrointestinal exposure and interactions with intestinal epithelial or immune cells. Grape-derived nanoparticles were linked to intestinal stem-cell and β-catenin-associated regenerative activity, whereas broccoli-derived nanoparticles showed transfer- and AMPK-deficiency-supported regulation of tolerance-associated dendritic-cell states (7, 8). Ginger- and turmeric-derived particles have been associated with macrophage-state changes, inflammatory regulation, epithelial protection, and microbiota alterations, although the causal contributions of vesicle structure, lipids, RNAs, and co-isolated phytochemicals remain incompletely resolved (9, 10). Hyaluronic acid-modified red cabbage-derived particles showed increased epithelial and immune-cell association relative to unmodified particles, but receptor-dependent targeting was not demonstrated (11). Existing reviews have summarized PELN sources, preparation methods, and therapeutic applications (12–14); the key unresolved issue is how their reported immune and regenerative activities should be functionally validated.

Translation is further complicated by heterogeneity in product identity, isolation, characterization, dose definition, stability, and manufacturing. MISEV2023 emphasizes rigorous nomenclature, pre-analytical control, separation and characterization procedures, and functional analysis of EV release, uptake, and biological activity (15). Reviews of EV clinical development have likewise identified incomplete reporting, uncertain EV-subpopulation definitions, and limited standardization across studies (16). Engineered, cargo-loaded, milk-derived, and plant-derived products introduce additional requirements involving comparability, impurities, altered biodistribution, immunogenicity, source control, and batch-to-batch consistency (17). These development issues should be linked to biological mechanism: a clinically relevant product requires not only reproducible manufacture but also a potency assay that reflects its intended immunomodulatory or regenerative function.

**Figure 1:**
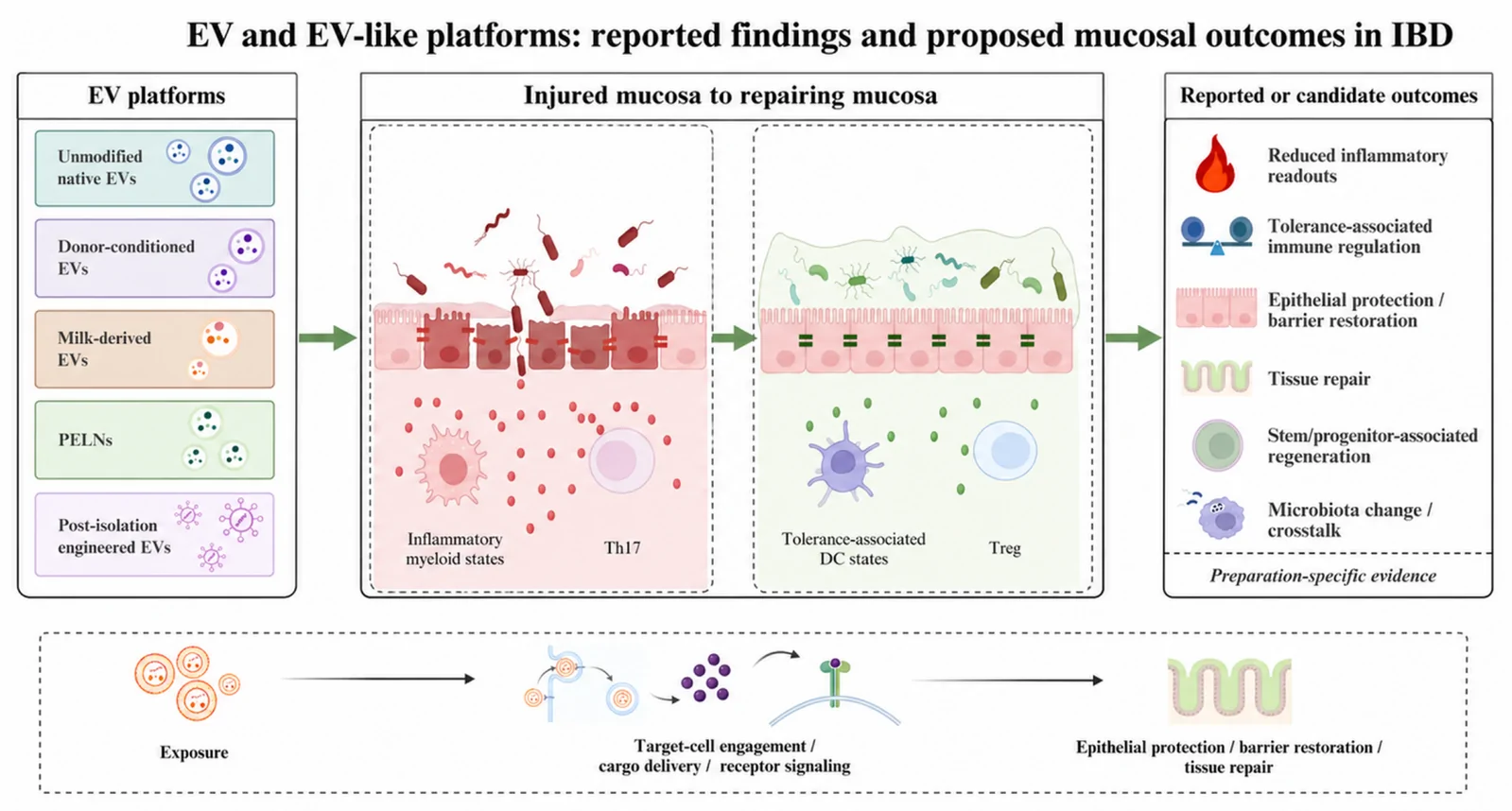
EV and EV-like platforms for immunomodulation and mucosal repair in IBD. Unmodified native EVs, donor-conditioned EVs, milk-derived EVs, plant-derived EV-like nanoparticles (PELNs), and post-isolation engineered EVs are shown alongside inflammatory and repair-associated mucosal states. Reported or proposed outcomes include reduced inflammatory activity, immune regulation, epithelial protection, barrier restoration, tissue repair, and microbiota changes. Evidence for stem/progenitor-associated regeneration is more limited and should be distinguished from epithelial protection. Exposure, target-cell engagement, intracellular cargo delivery, and receptor signaling represent distinct aspects of activity. Arrows indicate conceptual relationships, not established causal pathways shared by all preparations.

This structured narrative review evaluates native and engineered mammalian EVs, milk-derived EVs, and PELNs as candidate immunomodulatory and regenerative therapies for IBD. Rather than providing another source-based catalogue of vesicle preparations or anti-inflammatory outcomes (18), we organize the evidence around three questions. First, which myeloid, lymphocyte, epithelial, and regenerative pathways are associated with or causally involved in therapeutic activity? Second, how should mucosal exposure, disease-relevant cell engagement, functional cargo delivery or receptor signaling, pharmacodynamic immune regulation, and tissue repair be distinguished experimentally? Third, which product-identity, potency, safety, manufacturing, and human-model requirements should determine whether a candidate progresses toward clinical development? By integrating immune mechanism, engineering, functional targeting, and translational validation, this review aims to define the evidence needed to convert heterogeneous vesicle preparations into reproducible immune-regenerative therapies for IBD (**Figure 1**).

## 2 Scope, terminology, and conceptual framework

### 2.1 Literature identification and evidence selection

This structured narrative review was informed by PubMed and OpenAlex searches from inception through 5 August 2026, with Crossref used for metadata checks, Google Scholar for targeted supplementary searching, and ClinicalTrials.gov for interventional records. The search combined terms for IBD and intestinal inflammation with mammalian EVs, milk-derived EVs, PELNs, engineered vesicle delivery, immune-regenerative mechanisms, and translation. Full concepts, search expressions, and database records are reported in **Supplementary Tables S1, S2**.

Representative studies were selected for direct IBD relevance, platform distinctiveness, mechanistic or delivery evidence, translational value, and informative null or harmful findings. Evidence descriptors were assigned to individual claims. ‘Perturbation support’ required blockade, loss-of-function, rescue, transfer, or equivalent dependency testing, whereas a ‘defined link’ indicated a cargo-, surface-signal-, formulation-, or pathway-associated finding without decisive dependency testing. No meta-analysis, formal risk-of-bias assessment, or certainty grading was performed. Eligibility rules, study dispositions, and study-level data are reported in **SSupplementary Tables S3, S5**.

### 2.2 Product terminology and platform taxonomy

Consistent with current extracellular-vesicle nomenclature, EV is used as the generic term for lipid-bilayer particles released from mammalian cells or recovered from mammalian biofluids unless a specific biogenetic origin has been demonstrated (15, 19). The term exosome is therefore not used as an author-defined synonym for a small EV. When an original report or registered product uses exosome in its title or product name, that wording is retained only for accurate identification and does not imply independent confirmation of endosomal origin. Source-qualified or operational terms are preferred throughout the synthesis.

The platform taxonomy uses two complementary axes: biological source and modification status. Mammalian products are classified as (i) unmodified native EVs, obtained without intentional donor programming or post-isolation modification; (ii) donor-conditioned or donor-programmed EVs, produced after pharmacological, cytokine, hypoxic, metabolic, or genetic manipulation of the source cells; or (iii) post-isolation engineered EV products, including cargo-loaded, surface-modified, hybrid, chemically modified, or biomaterial-formulated preparations (20). Donor-conditioned products are not grouped with unmodified native EVs, and increased yield or selected bioactivity after donor manipulation is not interpreted as evidence of manufacturing scalability, batch equivalence, or long-term safety.

Milk-derived EVs are identified as mammalian biofluid EVs whose product attributes are additionally shaped by the food matrix, processing, digestion, storage, and purification. Plant- and food-derived preparations are described operationally as plant-derived EV-like nanoparticles (PELNs) unless vesicular identity and biogenesis are adequately established; minimally processed plant particles are distinguished from particles recovered after decoction, heating, or extensive extraction because processing may alter particle identity and co-isolated phytochemicals. Probiotic- or commensal-derived preparations are termed bacterial EVs rather than exosomes and are treated as a separate source class because their composition, innate-immune activity, safety controls, manufacturing requirements, and regulatory interpretation differ from mammalian and plant platforms. Engineering status is reported in addition to, rather than instead of, source identity.

### 2.3 Evidence domains and interpretive boundaries

Four distinct claims recur in EV and PELN studies: tissue exposure, target-cell engagement, functional intracellular delivery, and surface-receptor signaling. Fluorescent accumulation or cellular internalization supports the first two claims only. Functional delivery requires evidence that cargo reaches and acts in the relevant intracellular compartment, whereas surface signaling requires receptor engagement and a downstream response. Preferential localization to a disease-relevant cell population is additionally needed to support a targeting claim (21).

Throughout this review, the wording reflects how directly the experiment tests mechanism. “Associated with,” “accompanied by,” and “reported” describe co-occurrence. “Linked to” or “supported” indicates a defined relationship among cargo, formulation, surface signal, pathway, and function when dependency has not been established. Terms such as “mediated by,” “dependent on,” and “required” are reserved for blockade, loss-of-function, rescue, transfer, receptor-dependence, or comparable intervention studies. These descriptors characterize mechanistic resolution; they are not formal ratings of internal validity, risk of bias, or certainty of therapeutic benefit.

## 3 Mammalian EV platforms: unmodified native, donor-conditioned, and milk-derived EVs

Mammalian EV evidence is predominantly preclinical and preparation-specific. Unmodified native, donor-conditioned, and milk-derived platforms are considered here; post-isolation engineered products are addressed in Section 5.

### 3.1 Unmodified MSC-EVs and mucosal immune regulation

Unmodified MSC-EVs are the most extensively studied mammalian EV platform in experimental IBD. Here, the term refers to preparations produced without deliberate donor conditioning. These EVs can reproduce selected immune-regulatory and tissue-repair effects of parental MSCs without the hazards of administering viable cells, including persistent engraftment, ectopic tissue formation, and vascular obstruction. They nonetheless contain biologically active proteins, lipids, and RNAs that may affect myeloid cells, lymphocyte differentiation, inflammatory signaling, and epithelial survival. Risks related to proliferative, angiogenic, or immunosuppressive activity therefore remain relevant (22).

Human umbilical cord MSC-EVs have been tested in several preclinical colitis models, with reviews reporting anti-inflammatory, immune-regulatory, and repair-associated outcomes (23). The evidence is heterogeneous. Donor source, passage, baseline culture, oxygen tension, isolation, storage, and assay design can all change composition and measured activity. Preparations generated after inflammatory, cytokine, pharmacological, metabolic, hypoxic, or genetic programming belong to the donor-conditioned category and should be evaluated separately.

Myeloid-cell changes are a recurring observation. In one bone-marrow MSC-EV study, lower disease activity, histological injury, and inflammatory readouts coincided with increased CD163 expression and JAK/STAT-associated changes (24). The absence of macrophage depletion, cell-specific pathway disruption, and transfer experiments prevents a causal assignment to macrophage reprogramming. This distinction matters because human IBD mucosa contains diverse monocyte, macrophage, and neutrophil states that are not captured by a binary polarization model. More precise descriptions include myeloid-state modulation, reduced inflammatory activation, or increased resolution-associated markers.

The TSG-6 study provides a more direct cargo-function link. Knocking down exosomal TSG-6 weakened the reduction of NLRP3/caspase-1/GSDMD-associated pyroptosis and the recovery of tight-junction readouts in DSS colitis (25). The result implicates TSG-6 in that preparation, although the acute model and incomplete recipient-cell pathway testing limit broader inference.

### 3.2 Donor-conditioned and preparation-specific cargo-linked findings

Several hUC-MSC-EV studies have examined preparation-specific RNA cargo and downstream molecular targets. In preparations not reported as deliberately donor-conditioned, EV-associated miR-326 was linked to neddylation-related inflammatory signaling, and miR-129-5p was linked to ACSL4-related epithelial ferroptosis and mucosal injury (26, 27). By contrast, the IFN-γ-promoted MSC-EVs associated with miR-125a/miR-125b and STAT3-related regulation of Th17 differentiation are classified as donor-conditioned rather than native EVs (28). This reclassification identifies donor programming as part of the product definition and does not imply that the reported RNA was the sole active component.

These cargo-pathway findings are preparation-specific and do not support a shared MSC-EV mechanism. Cargo profiling and target assays can define a link, but intracellular delivery *in vivo* and product-specific dose-response still require direct testing. Changes in Treg/Th17 balance may also arise indirectly through antigen-presenting cells or epithelial recovery and do not identify T cells as the primary EV target.

A recent donor-conditioning example used exosomes from IL-33-stimulated macrophages in TNBS colitis and compared them with exosomes from untreated macrophages. The preparation was associated with improved epithelial-barrier outcomes and increased Wnt/beta-catenin-related readouts. Because no cargo loss-of-function, receptor blockade, or pathway-dependence experiment was reported, the findings support a donor-conditioning and barrier-repair association rather than a defined IL-33-exosome mechanism (29). LPS-pretreated PDLSC-EVs also outperformed unconditioned PDLSC-EVs in acute DSS colitis across disease activity, barrier, macrophage-state, and microbiota readouts; because PI3K/AKT dependency was not tested, the study supports donor-conditioning benefit and a pathway association, not a proven PI3K/AKT mechanism (30).

### 3.3 Milk-derived EVs and innate immune–epithelial crosstalk

Milk-derived EVs represent a distinct, generally unmodified mammalian biofluid EV source and are particularly relevant to oral intestinal exposure. In experimental UC, orally administered milk-derived EVs were associated with reduced TLR4-NF-κB- and NLRP3-related readouts, a shifted Treg/Th17 balance, and partial recovery of gut microbiota composition (31). These findings report changes across several connected domains, including innate inflammatory sensing, adaptive immune balance, epithelial protection, and microbial ecology, but do not establish that all domains contributed causally to the treatment effect.

Because no pathway-dependence experiment was performed, the TLR4-NF-κB and NLRP3 changes remain associated readouts.

The reported microbiota changes should also be interpreted cautiously. Microbial community recovery may contribute to reduced immune stimulation and improved barrier function, but it may alternatively occur as a secondary consequence of reduced inflammation or altered intestinal physiology. Without antibiotic depletion, germ-free experiments, microbiota transfer, metabolite rescue, or a comparable intervention experiment, microbiota normalization remains an associated ecological outcome rather than a demonstrated mediator.

Milk-derived EVs also raise distinctive product-definition questions. Their composition may be affected by animal species, lactation state, food matrix, pasteurization or other processing, digestion, storage, purification, and co-isolated proteins or lipids. The distinction between nutritional exposure and therapeutic dosing is especially important. A reproducible therapeutic milk-EV product requires defined source material, dose metrics, gastrointestinal stability, active-component attribution, and potency assays linked to its intended immune or epithelial mechanism.

### 3.4 Mechanistic maturity, model limitations, and clinical relevance

Systematic analyses of heterogeneous mammalian EV interventions in rodent colitis report lower disease activity, histological injury, myeloperoxidase activity, TNF-α, NF-κB, IL-1β, and IL-6, with higher IL-10 (5). A later review reached a similar descriptive conclusion while noting insufficient safety assessment (32). These pooled associations do not identify a class-wide mechanism or separate all unmodified from donor-conditioned preparations.

Most studies use acute DSS or TNBS colitis, which models epithelial injury, innate inflammation, and early repair but not chronic, relapsing, transmural, fibrotic, fistulizing, or postoperative IBD. Mammalian EV efficacy in these models therefore supports biological plausibility, not clinical readiness.

Clinical evidence is limited to a small uncontrolled phase I study in five patients with refractory perianal Crohn’s disease fistulas: four were responders at six months, three achieved complete healing, and no local or systemic adverse events were reported (33). This is a local feasibility and safety signal, not evidence for luminal UC or CD or for oral or systemic administration.

### 3.5 Negative and counter-evidence: when EVs fail or exacerbate inflammation

The therapeutic direction of an EV preparation cannot be inferred from vesicle identity alone. Available counter-evidence indicates at least three distinct failure modes: loss of particle or cargo integrity during administration, insufficient intrinsic activity of an unloaded or unmodified preparation, and source- or cargo-associated signaling that may aggravate rather than resolve inflammation.

MSC-derived EVs have also produced effects that diverged from those of their parental cells in the same DSS-colitis setting: cytokine-primed MSC-EVs improved clinical, histological, and macrophage-associated readouts, whereas administration of the corresponding MSCs worsened disease. This comparison reinforces that vesicle activity cannot be inferred directly from source-cell behavior and should be tested for each preparation, dose, and disease context (34).

The 2025 bovine milk-EV study provides a direct example of formulation and activity failure. Milk-EV membranes were disrupted in biorelevant gastrointestinal fluids, and unloaded vesicles did not improve DSS colitis after either oral or rectal administration (35). These results do not invalidate protected or cargo-loaded milk-EV systems, but they demonstrate that food-matrix origin, particle recovery, gastrointestinal stability, dose, and retained cargo must be verified rather than assumed. Unloaded-vesicle and disrupted-particle controls are therefore essential when therapeutic activity is attributed to the carrier itself.

Counter-evidence also shows that vesicular uptake can be associated with harmful biological effects. Epithelial EVs carrying iron-loaded ferritin were taken up by macrophages through MSR1 and exacerbated oxidative inflammatory responses in experimental IBD (36). This study supports a preparation-specific ferritin-MSR1 inflammatory mechanism under the tested conditions; it does not establish that epithelial EV uptake is generally harmful. The finding nevertheless illustrates how EV source, cargo composition, recipient-cell state, dose, and disease context can change the direction of biological activity. Related safety concerns are particularly relevant to checkpoint-displaying, cytokine-loaded, or regenerative platforms, in which excessive immune suppression, off-target signaling, or sustained proliferative stimulation could offset local benefit.

Negative and harmful findings are therefore retained in **Table 1**, and future studies should prespecify failure criteria and appropriate inactive-product and pathway controls.

**Table 1:** Multidimensional evidence profile of representative EV-based and EV-like IBD studies.

Panel A. Study context and principal limitations.					
Platform/study	Model/route	Intervention and principal readout		Main limitation	
Unmodified BM-MSC EVs - Cao et al. (24)	DSS colitis; systemic	Unmodified EV preparation; macrophage-state and cytokine readouts; DAI and histology		Acute model; macrophage-state association without cell-specific dependency testing	
Unmodified hUC-MSC EVs - miR-326 study (26)	DSS colitis; systemic	EV-associated miR-326; inflammation and mucosal injury; neddylation-related link		Preparation-specific cargo/pathway link; intact intracellular delivery and human relevance unresolved	
Unmodified hUC-MSC EVs - miR-129-5p study (27)	DSS colitis; systemic	EV-associated miR-129-5p; epithelial ferroptosis; ACSL4-related link		Preparation-specific cargo/target link; intact delivery and human relevance unresolved	
Unmodified hUC-MSC exosomes - Liang et al. (95)	DSS and TNBS colitis; experimental organoids; intraperitoneal	Lgr5, Ki-67, EdU, and organoid-growth readouts; Wnt-C59 attenuation of repair		Pharmacologic pathway support without lineage tracing, durable differentiation, or long-term functional recovery	
iPSC-MSC-derived exosomes - Feng et al. (96)	TNBS colitis; patient CD LPMCs; intraperitoneal	Lgr5/Bmi1/Ki-67 readouts; miR-34a-5p gain/loss; PPP2R3A/Wnt link		Cargo-target evidence with 7-day follow-up; no lineage tracing or durable tissue-function validation	
Donor-conditioned PDLSC EVs - Tang et al. (30)	Acute DSS colitis; systemic	LPS-pretreated versus unconditioned PDLSC EVs; barrier, myeloid-state, microbiota, and PI3K/AKT readouts		Conditioning benefit shown, but PI3K/AKT dependency, active cargo, and chronic or human relevance remain unresolved	
Unloaded bovine milk EVs - Seegobin et al. (35)	Biorelevant GI fluids; DSS colitis; oral/rectal	Unloaded milk EVs; membrane disruption; no improvement in DSS colitis		Preparation- and processing-specific negative result; does not exclude protected or loaded formulations	
Cargo-engineered milk EVs - anti-TNF-alpha nanobody/LL37 (67)	DSS colitis; oral	Nanobody and LL37 loading; barrier, cytokine, and antimicrobial-associated readouts		Functional delivery, digestion, dose, residual free cargo, and comparison with unloaded EVs remain uncertain	
Grape EV-like nanoparticles (7)	DSS; experimental organoids; oral	Natural lipid-rich particles; stem/progenitor engagement; lipid and beta-catenin perturbation; tissue repair		Mouse and experimental-organoid evidence; active molecule and intact delivery uncertain	
Broccoli-derived nanoparticles (8)	Mouse colitis; oral	Plant lipids and sulforaphane-associated activity; tolerance-associated DC states; AMPK dependence and DC transfer		No human intestinal DC or mucosal validation	
Ginger-derived nanoparticles (9)	DSS/CAC models; oral	Lipids, miRNAs, and ginger compounds; exposure, cell uptake, cytokine and survival readouts		Particle-versus-compound causality and functional delivery remain unclear	
Turmeric-derived nanovesicles (10, 44)	DSS colitis; oral	Distinct preparations with curcumin-associated cargo; macrophage, microbiota, barrier, HO-1, and NF-kappaB readouts		Preparation-specific findings should not be merged; cargo-microbiota-barrier causal chain unresolved	
Momordica charantia-derived EVs - Gao et al. (54)	DSS colitis; oral	Lipid/carotenoid-associated cargo; inflammatory, oxidative, microbiota, and barrier readouts		Cargo-function causality and human mucosal validation remain limited	
Post-isolation engineered PELNs - HA-red cabbage (11)	Experimental colitis; oral	HA surface conjugation; increased epithelial and immune-cell association; inflammatory/barrier readouts		Preferential association shown; receptor dependence, off-target distribution, and repeat-dose safety incomplete	
Post-isolation checkpoint/cargo-engineered EVs - PD-L1/miR-27a-3p (6)	IBD models; patient cells and colonoids	PD-L1 surface signal and miR-27a-3p cargo; PD-1/PHB1 branches; immune and barrier readouts		Preparation-specific human-relevant experiments; no clinical efficacy and repeat-dose immunosuppression unresolved	
Biomaterial-retained unmodified MSC-EVs - Gan et al. (71)	Acute colitis; oral	Alginate/gelatin/enteric microcapsules; gastrointestinal protection and local release		Formulation/retention evidence; cell-specific functional delivery and repeat-dose safety not established	
Donor-conditioned and formulated Gal-IL10-EVs - Liu et al. (64)	Murine colitis; oral	IL-10 donor-cell engineering; reported macrophage engagement; chitosan/alginate protection		Must be compared with unmodified EVs; intact delivery, free IL-10, and off-target distribution unresolved	
Donor-conditioned MSC-IL-27 EV microcarriers - Nie et al. (72)	IBD rats; rectal	IL-27-high donor cells; EV microcarriers; bioadhesion, sustained release, and barrier readouts		Donor conditioning and formulation are separate modifications; target-cell engagement and chronic safety unresolved	
Cargo-engineered milk EVs carrying TNF-alpha siRNA - Han et al. (68)	DSS colitis; oral	Electroporation-loaded siRNA; gastrointestinal stability; TNF-alpha target suppression		Free-siRNA contribution, functional compartment delivery, milk-source variation, and repeat-dose safety unresolved	
Protein-hybrid EVs - Lee et al. (69)	Murine IBD organoids and colitis; oral	Backbone-cyclized immunoregulatory protein; acid resistance; barrier/inflammatory readouts		Loading comparability and functional compartment delivery require validation	
Post-isolation hybrid PrEXO-a23 vesicles - Cao et al. (65)	Murine colitis, fibrosis, and CAC; systemic	Treg-EV/platelet-membrane hybrid; MMP-cleavable anti-IL-23; DC/Treg-associated regulation		No human mucosal validation; complex manufacture and comparison with IL-23 biologics unresolved	
Unmodified MSC-EV preparation reported as exosomes - perianal CD phase I (33)	Human fistula; local injection	Local administration; preliminary safety, feasibility, and reported clinical response		Five-patient uncontrolled cohort; localized indication; not evidence for luminal UC/CD efficacy	
Panel B. Independent evidence domains.					
Study	Model relevance	Component-specific mechanistic evidence	Delivery evidence	Development stage/principal gaps	Clinical status
Cao et al. (24)	Acute rodent	Macrophage-state association; dependency not tested	Not resolved	Exploratory animal preclinical; identity, dose, and potency gaps	No clinical evidence
miR-326 EVs (26)	Acute rodent	Defined cargo/pathway link; class-wide mechanism not established	Cargo activity supported; intact delivery unresolved	Exploratory animal preclinical; human relevance and release assay absent	No clinical evidence
miR-129-5p EVs (27)	Acute rodent	Defined cargo/target link; preparation-specific	Cargo activity supported; intact delivery unresolved	Exploratory animal preclinical; human relevance and chronic safety absent	No clinical evidence
Liang et al. (95)	Acute rodent + experimental organoids	Wnt-C59 attenuation supports a Wnt/beta-catenin-related repair branch	Cell and pathway engagement; intact cargo delivery unresolved	Exploratory animal/organoid preclinical; no lineage tracing, durable differentiation, or long-term function	No clinical evidence
Feng et al. (96)	Acute rodent + patient CD LPMCs	miR-34a-5p gain/loss and PPP2R3A targeting support a preparation-specific cargo/pathway branch	Cargo activity supported; intact intracellular delivery unresolved	Human-cell-supported preclinical; 7-day follow-up; no lineage tracing or durable tissue-function validation	No clinical efficacy evidence
Tang et al. (30)	Acute rodent	Donor-conditioning benefit and PI3K/AKT association; pathway dependency not tested	Colon exposure reported; functional cargo delivery unresolved	Conditioned animal preclinical; active cargo, chronic models, and human validation absent	No clinical evidence
Unloaded bovine milk EVs (35)	Rodent + GI-fluid testing	Negative/counter-evidence for tested preparation	Membrane stability not maintained; no therapeutic delivery	Exploratory negative preclinical; formulation and product-definition gaps	No clinical evidence
Nanobody/LL37 milk EVs (67)	Acute rodent	Defined cargo/function link; dependency breadth limited	Cargo-associated activity; free-cargo contribution unresolved	Engineered animal preclinical; dose, comparability, and safety gaps	No clinical evidence
Grape nanoparticles (7)	Rodent + experimental organoids	Lipid/beta-catenin perturbation supports a regeneration-related branch	Cell engagement; intact delivery unresolved	Exploratory animal/organoid preclinical; active-component and product-control gaps	No clinical evidence
Broccoli nanoparticles (8)	Rodent	AMPK and DC-transfer support for a DC-related branch	Cell/pathway engagement; cargo delivery unresolved	Exploratory animal preclinical; no human mucosal validation	No clinical evidence
Ginger nanoparticles (9)	Rodent multi-model	Descriptive multi-component associations	Cell engagement reported	Exploratory animal preclinical; particle/cargo attribution unresolved	No clinical evidence
Turmeric nanovesicles (10, 44)	Rodent	Preparation-specific descriptive associations	Tissue exposure; functional delivery unresolved	Exploratory animal preclinical; distinct preparations and causal chain unresolved	No clinical evidence
Gao et al. (54)	Rodent	Descriptive cargo/outcome associations	Not resolved	Exploratory animal preclinical; human relevance and active-component gaps	No clinical evidence
HA-red cabbage particles (11)	Rodent	Modification/function link; receptor dependence not established	Increased cell association; functional targeting incomplete	Engineered animal preclinical; off-target and repeat-dose gaps	No clinical evidence
PD-L1/miR-27a-3p EVs (6)	Rodent + patient-derived systems	Perturbation support for PD-1/PHB1-related branches	Surface-signal and cargo activity supported; full sequence not established	Human-relevant experimental preclinical; manufacturing and chronic-safety gaps	No clinical efficacy evidence
Gan et al. (71)	Acute rodent	Formulation/retention-function link	Exposure/retention; cell delivery unresolved	Formulated animal preclinical; comparability and repeat-dose gaps	No clinical evidence
Liu et al. (64)	Rodent	Donor-conditioning/formulation link; partial perturbation	Cell engagement and cargo activity reported; intact delivery unresolved	Multi-modified animal preclinical; unmodified comparator and product-control gaps	No clinical evidence
Nie et al. (72)	Rodent	Donor-conditioning/formulation-function link	Exposure/retention; cell delivery unresolved	Multi-modified animal preclinical; target-cell and chronic-safety gaps	No clinical evidence
Han et al. (68)	Rodent	Defined siRNA/target link	Cargo activity supported; free cargo and compartment delivery unresolved	Engineered animal preclinical; oral dose, source, and repeat-dose gaps	No clinical evidence
Lee et al. (69)	Rodent + experimental organoids	Defined protein/function link; partial perturbation	Cargo-associated activity; compartment delivery unresolved	Organoid-supported preclinical; loading comparability and safety gaps	No clinical evidence
Cao et al. (65)	Rodent multi-model	Perturbation support for selected surface/cargo branches	Surface/cargo activity supported; full delivery sequence incomplete	Complex engineered animal preclinical; comparator, manufacturing, and safety gaps	No clinical evidence
Perianal CD phase I (33)	Human; localized fistula phenotype	Not assessed	Local administration; target-cell delivery unresolved	Early feasibility/safety signal; efficacy unestablished	Phase I; five-patient uncontrolled cohort

‘Perturbation support’ requires blockade, loss-of-function, rescue, transfer, or receptor-, target-, or pathway-dependence testing. A ‘defined link’ denotes cargo-, surface-signal-, formulation-, or pathway-associated functional findings without decisive dependency testing. ‘Cargo/surface-signal activity supported’ indicates compatible downstream activity, not proof of intact-vesicle cytosolic delivery. ‘Negative/counter-evidence’ denotes absent therapeutic benefit or failure of a relevant product-performance criterion. These descriptors apply to specific claims, not overall study quality, risk of bias, or certainty of benefit; registry-only records are not clinical efficacy evidence. Detailed criteria and study-level assignments are provided in **Supplementary Tables S3** and **S5**.

BM-MSC, bone marrow mesenchymal stromal/stem cell; DC, dendritic cell; EV, extracellular vesicle; HA, hyaluronic acid; hUC-MSC, human umbilical cord mesenchymal stromal/stem cell; IBD, inflammatory bowel disease; PELNs, plant-derived EV-like nanoparticles.

Panel A summarizes study context, product classification, the principal unresolved limitation, and representative negative/counter-evidence. Panel B presents model relevance, component-specific mechanistic evidence, delivery evidence, development stage and gaps, and clinical status as independent non-ordinal domains. No composite ranking was assigned, and the descriptors do not constitute ratings of overall study quality, risk of bias, or certainty of therapeutic benefit.

Native, donor-conditioned, and milk-derived EVs remain distinct preclinical platforms. Uncertain active components, variable tropism, and limited causal testing prevent class-wide mechanistic or clinical claims.

## 4 Plant-derived EV-like nanoparticles as modulators of mucosal immunity and regeneration

Plant-derived EV-like nanoparticles (PELNs) are emerging as candidate modulators of intestinal immunity and tissue repair. Their potential relevance to inflammatory bowel disease (IBD) arises from the combination of oral mucosal exposure, interactions with epithelial and immune-cell compartments, and preparation-specific lipid, RNA, protein, metabolite, or surface-associated activity. Edible plant sources may also offer practical advantages for repeated intestinal administration and scalable starting-material supply. However, PELNs should not be treated as a uniform therapeutic class. Gastrointestinal stability, mucosal distribution, cellular tropism, active-component identity, immune regulation, regenerative activity, and manufacturing consistency may differ substantially among plant species and preparation methods.

A recent meta-analysis of animal UC studies reported pooled improvements in disease activity, colon length, histological injury, inflammatory mediators, oxidative-stress measures, and barrier-related outcomes after PELN treatment. However, substantial heterogeneity in plant source, preparation, dose, model, and outcome measurement limits the result to an evidence-level preclinical association and does not support class-wide efficacy or clinical extrapolation (37).

### 4.1 Oral exposure and mucosal access

Oral delivery gives plant-derived particles direct access to the intestinal lumen. Exposure still depends on survival through acid, digestive enzymes, bile, mucus, transit, and microbial metabolism (38, 39). Inflammation can further alter mucus, permeability, residence time, and regional access.

Size, lipid architecture, and surface chemistry may protect particle-associated molecules during transit (38, 40), and some preparations retain particle or cargo features after simulated digestion (41, 42). These findings establish gastrointestinal stability, not mucosal penetration or signaling. A complete delivery analysis should follow the preparation from luminal integrity to regional exposure, cell engagement, and the intended functional response.

Cell-interaction studies illustrate the gap between exposure and mechanism. Edible-plant nanoparticles can interact with intestinal cells and macrophages (43). Ginger nanoparticles accumulate in the colon and enter epithelial cells and macrophages (9). Turmeric preparations show colonic exposure with anti-colitis activity (10, 44). These data identify candidate sites of action. They do not yet resolve the active component, the relevant intracellular compartment, or whether cellular uptake is necessary for benefit. We therefore classify localization and uptake under the delivery criteria defined in Sections 2.3 and 8.4.

### 4.2 Tolerance-associated dendritic-cell states and innate immune regulation

Dendritic cells are central to the interface among luminal antigens, commensal microorganisms, epithelial signals, and adaptive immunity. Under homeostatic conditions, intestinal dendritic cells contribute to antigen sampling, oral tolerance, regulatory T-cell induction, and controlled responses to food- and microbiota-derived antigens. During intestinal inflammation, altered dendritic-cell activation, metabolism, and cytokine production can instead promote pathogenic effector-cell responses and sustain mucosal injury (45, 46).

Broccoli-derived nanoparticles provide one of the most informative PELN examples because the proposed immune mechanism was examined using intervention experiments. Orally administered broccoli nanoparticles protected mice in several colitis models and acted on dendritic cells (8). Transfer of dendritic cells exposed to broccoli nanoparticle lipids reproduced part of the protective effect. Comparison with sulforaphane-depleted lipid fractions and loss of protection in mice receiving AMPK-deficient dendritic cells further supported a sulforaphane-related, dendritic-cell AMPK-dependent mechanism.

AMPK-deficient dendritic-cell experiments provide stronger mechanistic support than treatment-associated cytokine changes, but only for this preparation. The contributions of sulforaphane, associated lipids, particle architecture, and other components, and the reproducibility of the response in human intestinal dendritic-cell systems, remain unresolved.

### 4.3 Macrophage-state regulation and inflammatory resolution

Macrophages are another recurring immune target in PELN studies. Intestinal macrophages participate in microbial sensing, cytokine production, phagocytosis, inflammatory amplification, tissue injury, resolution, and repair. Their functions are shaped by local cytokines, microbial products, epithelial-derived signals, metabolites, and disease stage. Accordingly, PELN-associated macrophage findings should be interpreted as changes in context-dependent inflammatory or repair-associated myeloid states rather than conversion between fixed polarization categories (47, 48).

Ginger-derived nanoparticles illustrate multi-compartment mucosal activity. After oral administration, these particles were taken up mainly by intestinal epithelial cells and macrophages in experimental colitis. Treatment was accompanied by reduced TNF-α, IL-6, and IL-1β, increased IL-10 and IL-22, and greater epithelial-cell survival and proliferation (9). These findings are consistent with reduced inflammatory myeloid activity and a mucosal environment more supportive of epithelial repair. However, because ginger-derived preparations contain lipids, microRNAs, 6-gingerol, 6-shogaol, and potentially other associated compounds, the relative contribution of each component remains unresolved.

Turmeric-derived vesicle-like nanoparticles provide a second example. Oral treatment was associated with inflamed-colon accumulation, changes in macrophage-related readouts, microbiota shifts, and improved epithelial-barrier outcomes in DSS colitis (10). A separate turmeric preparation containing curcumin was associated with reduced TNF-α, IL-6, and IL-1β expression, increased HO-1 expression, and lower NF-κB reporter activity (44). These observations are compatible with anti-inflammatory and antioxidant pathway involvement, but they do not establish a causal sequence linking vesicle structure, curcumin, macrophage regulation, microbiota changes, and epithelial repair.

These macrophage-associated changes remain non-causal without depletion, lineage-restricted perturbation, uptake blockade, transfer, or rescue experiments. Human studies should identify the engaged myeloid state and test whether its alteration is required for tissue protection rather than relying on binary polarization markers.

### 4.4 Epithelial renewal and regenerative signaling

Selected PELNs may influence epithelial repair and regenerative signaling in addition to immune regulation. This distinction is important because durable mucosal healing requires restoration of epithelial continuity, crypt architecture, tight-junction function, and wound-repair capacity rather than cytokine suppression alone. Lgr5-positive crypt base columnar cells are established intestinal epithelial stem cells, and individual Lgr5-positive cells can generate self-organizing intestinal organoids (49, 50).

Grape-derived exosome-like nanoparticles provide a representative regeneration-oriented example. In experimental colitis, grape-derived nanoparticles engaged intestinal stem cells, enhanced organoid formation, and improved tissue-remodeling outcomes (7). Blocking β-catenin signaling attenuated induction of Lgr5-positive cells, while liposome-like particles reconstructed from grape nanoparticle lipids reproduced part of the reported stem-cell interaction. These experiments support lipid-associated and β-catenin-related pathway involvement.

These experiments support preparation-specific lipid/β-catenin involvement, but do not fully define intact-particle delivery or justify generalization to other PELNs or human IBD. Human organoid, explant, and immune-epithelial co-culture studies should distinguish direct progenitor engagement from repair secondary to reduced inflammation (51).

### 4.5 Microbiota–immune–barrier crosstalk

Microbiota composition, epithelial integrity, and mucosal immunity often change together after PELN treatment. Their coupling complicates causal interpretation. Dysbiosis can increase inflammatory microbial products and reduce protective metabolic functions, whereas barrier failure promotes microbial translocation and innate activation. In the opposite direction, inflammation and altered intestinal physiology can reshape the microbial community (52, 53).

Ginger-, turmeric-, and Momordica charantia-derived preparations changed inflammatory mediators, oxidative-stress markers, microbiota composition, and barrier readouts in the same experiments (9, 10, 54). Such parallel effects are consistent with activity across a mucosal network, but they do not establish the direction of influence. A 2025 multi-omic study of Centella asiatica-derived vesicles offered a more specific model by linking inflamed-colon accumulation and plant miRNA cargo with immune-cell, microbiota, and systemic metabolic changes (55). The proposed cargo-to-network sequence still requires direct testing.

Newer plant studies span several levels of mechanistic evidence. Pueraria lobata particles lost activity in a pseudo-germ-free model, implicating a microbiota-dependent component. Flos Sophorae immaturus nanovesicles were studied with antibiotics, fecal microbiota transfer, mono-colonization, organoids, and AhR antagonism, defining a microbiota-tryptophan-AhR pathway more clearly. Extremophyte-derived nanovesicles improved barrier, immune, and microbiota readouts, but pathway dependence remained incomplete (56–58). Viewed together, these studies show why evidence should be compared by experimental depth instead of grouped under one microbiota mechanism.

Microbiota causality requires intervention, such as depletion, gnotobiotic reconstruction, transfer, metabolite rescue, or host-sensing blockade. Concurrent microbial, immune, and barrier changes otherwise remain associated network responses.

### 4.6 Product identity, causal attribution, and engineering transition

Plant-derived particles should be defined by their own product attributes. Their biogenesis, markers, separation behavior, and composition differ from those of mammalian EVs. This review therefore uses PELN as an operational term for vesicle-like nanoparticles with documented plant source, extraction, characterization, composition, and purity. Mammalian exosome markers are not assumed to apply.

Biological activity may arise from the particle, associated macromolecules, metabolites, surface components, co-isolated phytochemicals, or microbiota-mediated effects. Causal attribution therefore depends on free-compound, disrupted-particle, cargo-depletion, fractionation, or reconstitution controls. Localization studies also need to address label leakage, aggregation, and signal persistence after particle degradation. Cross-kingdom RNA claims require cargo quantification, stoichiometry, intracellular availability, target engagement, and functional sufficiency.

HA-modified red-cabbage particles illustrate the boundary between native and engineered plant platforms. Relative to unmodified particles, they showed greater gastrointestinal, epithelial, and immune-cell association together with greater preclinical efficacy (11). Without receptor blocking or ligand-dependence experiments, the study demonstrates an engineering-related gain in exposure and cell engagement, not validated HA-receptor targeting. Comparative biodistribution, off-target exposure, receptor dependence, and mechanism-linked pharmacodynamic readouts would define the claimed advantage more precisely.

Processing can also change product identity without creating a validated targeting mechanism. Thermal reassembly altered ginger-particle surface composition and uptake. Magnolia biondii decoction particles contained multiple metabolite classes. Portulaca oleracea particles were reported to carry miR159-y and modulate TGFBR2-related innate-lymphoid-cell plasticity (59–61). The last example raises a cross-kingdom cargo hypothesis that still depends on quantitative delivery and loss-of-function evidence.

Mechanistic depth remains uneven: dendritic-cell AMPK dependence and lipid/beta-catenin-associated repair are more directly tested than most macrophage, microbiota, inflammatory, and barrier claims. Manufacturing and safety are consolidated in Section 8 (20, 62).

## 5 Engineered EVs for programmable immunomodulation and regeneration

### 5.1 Why engineering is needed

Engineering is useful when it controls a therapeutic attribute that native EVs cannot deliver reproducibly. The relevant attributes include cargo identity, local exposure, cell recognition, release kinetics, and dose. Donor-cell programming, post-isolation loading, ligand display, membrane fusion, and responsive or bioadhesive formulations address different points in this chain. Each claimed advantage should be tested against unloaded, unmodified, free-cargo, and mock-engineered comparators and linked to a prespecified mechanism of action (15, 20, 62). A recent MSC-EV review places source selection, targeting, cargo engineering, and biomaterial integration within the same development framework (63).

### 5.2 Surface engineering for mucosal targeting

Surface engineering can alter mucosal adhesion, cellular association, receptor-dependent binding, or inflammatory-site homing, but the demonstrated level must be stated explicitly. Hyaluronic acid-modified red cabbage particles showed greater epithelial and immune-cell engagement than unmodified particles in mouse colitis; receptor dependence, off-target distribution, and repeat-dose safety remain incompletely defined (11). The evidence therefore supports improved engineered cell engagement, not yet a validated receptor-mediated targeting claim.

A second example combines surface and cargo engineering. Galactose-modified EVs produced by IL-10-programmed mammalian cells were encapsulated in a chitosan/alginate coating for oral delivery. The design protected IL-10 in the gastrointestinal tract and was intended to enhance delivery to inflammatory macrophages in the colonic lamina propria (64). This study links donor-cell cargo programming, ligand-guided cell engagement, and pH-responsive protection, but remains a murine proof-of-concept and requires quantitative comparison of intact EV delivery, free IL-10 exposure, and off-target distribution.

Hybrid membrane engineering provides a distinct targeting strategy. Treg-derived exosomes fused with platelet membrane vesicles and linked to anti-IL-23 through an MMP-cleavable linker preferentially accumulated in inflamed colon in murine models (65). MMP-responsive release and downstream dendritic-cell and Treg readouts support a surface/cargo mechanism supported by intervention evidence in the reported system. The platform was tested across acute colitis, fibrosis, and colitis-associated cancer models, but remains preclinical and has not established superiority to available IL-23 pathway inhibitors.

Because surface modifications act through different processes, each requires matched controls for dependence, biodistribution, cell engagement, signaling, and off-target exposure.

### 5.3 Cargo engineering for immune-regenerative circuits

Cargo engineering requires evidence of cargo stability, residual free cargo, vesicle integrity, target-cell delivery, target modulation, and dose response; uptake alone does not demonstrate cytosolic release or functional activity (15, 21, 66).

Milk-derived EVs illustrate two cargo-engineering strategies with relatively defined, but still incomplete, cargo–function relationships. In one study, TAT-assisted loading of an anti-TNF-α nanobody, alone or together with LL37, improved protection from gastrointestinal degradation and was associated with changes in inflammatory, barrier, antimicrobial, and microbiota-related readouts in murine colitis (67). Comparison with free nanobody supports a contribution from the EV formulation, but does not establish that each loaded component independently mediated every observed effect. In a separate study, milk exosomes carrying TNF-α siRNA preserved RNA during oral exposure, reduced TNF-α expression, and improved DSS colitis; the use of scrambled-siRNA and alternative-carrier controls strengthened the cargo-specific target relationship (68). However, neither platform has resolved clinically relevant oral dosing, milk-matrix variability, allergenicity, repeat-dose safety, or every step of intact-vesicle intracellular delivery.

Protein-loaded hybrid EVs provide another example of a defined cargo–formulation relationship. A backbone-cyclized immunoregulatory protein incorporated into naturally bioactive EVs showed improved protein stability, loading performance, anti-inflammatory activity, and oral efficacy in murine colitis and organoid systems (69). Comparisons among free protein, native EVs, and combined formulations support a formulation-dependent functional effect, while reproducible loading, intracellular compartment access, and the relative contribution of the native EV cargo remain unresolved.

Minimum controls include unloaded EVs, free cargo, mock-loaded or scrambled-cargo EVs, modified-versus-unmodified particles, dose response, and a target-engagement assay. Cargo-loaded vesicles remain preparation-specific modified biological products (62).

### 5.4 Checkpoint-engineered EVs at the innate–adaptive immune interface

Checkpoint-engineered EVs represent a mechanism-directed form of immune reprogramming. The PD-1/PD-L1 pathway restrains T-cell activation, but its therapeutic manipulation must preserve antimicrobial defense and tissue surveillance (70). Accordingly, checkpoint display should be evaluated through receptor dependence, downstream signaling, immune competence, and durability rather than through T-cell suppression alone.

PD-L1/miR-27a-3p EVs combine surface PD-L1 with an internal regulatory miRNA (6). Receptor and pathway experiments supported PD-1-dependent attenuation of T-cell-receptor signaling and miR-27a-3p/PHB1-related regulation of Th17 bioenergetics, together with preferential localization to inflamed intestine. Patient-derived immune-cell and colonoid experiments add human relevance but do not constitute an integrated mucosal model. The evidence supports this preparation-specific receptor/pathway mechanism, not class-wide functional cytosolic delivery.

Clinical superiority, optimal dosing, durability, infection risk, and repeat-dose safety remain unresolved.

### 5.5 Biomaterial-assisted retention and hybrid delivery

Biomaterial-assisted formulations address a different limitation from molecular cargo or ligand engineering: insufficient protection, residence time, or local exposure. MSC exosomes encapsulated in alginate/gelatin enteric microcapsules retained bioactivity during storage and gastrointestinal challenge and were released at intestinal sites in acute colitis models (71). In a rectal strategy, IL-27-high MSC-EVs were incorporated into dopamine-methacrylamide bioadhesive microcarriers to prolong colonic retention and support anti-inflammatory and barrier-repair effects in rats (72). These platforms provide direct evidence for oral protection and local adhesion, respectively, but do not by themselves prove cell-specific functional delivery.

For biomaterial-retained or hybrid vesicles, the formulation is part of the product rather than an inert accessory. Development should therefore measure vesicle integrity within the carrier, release kinetics, local dose, degradation products, mucosal toxicity, sterility, and manufacturability. Comparisons with free EVs and empty materials are required to determine whether the formulation adds therapeutic value rather than merely prolonging nonspecific exposure. A distinct *in situ* strategy used wireless mechanoelectronic stimulation of hydrogel-embedded autologous macrophages to increase therapeutic EV production and modulate gut microbiota in colitic mice, expanding hybrid delivery from passive retention to on-demand vesicle biogenesis (73). More recently, sequential lipid and sodium alginate coating of macrophage-derived EVs enabled pH-resistant oral 5-ASA delivery, delayed colonic release, improved epithelial transport and macrophage exposure, and restored barrier and inflammatory readouts in DSS colitis (74).

Recent local and oral systems include thermogels, double coatings, biohybrid or IgA/pIgR-directed vesicles, microbial EVs, drug-loaded plant particles, and bacteria-assembled platforms (75–81). Their diversity does not imply equivalent identity, mechanism, safety, or manufacturability.

Pasteurization-defined Akkermansia vesicles outperformed live-bacteria vesicles in acute DSS colitis and altered STING/IκB/NF-κB-associated readouts (82), supporting a processing-function association rather than proven pathway dependence.

Potential benefits from probiotic or commensal bacterial EVs should not be generalized to pathogenic or insufficiently defined bacterial vesicles. Bacterial source, strain, growth conditions, and processing can alter inflammatory cell-envelope components and other biologically active cargo; product development therefore requires strain-level identity, purification controls, endotoxin and impurity testing, innate-immune activation assays, and repeat-dose safety evaluation (62, 83).

### 5.6 Engineering risks and translational complexity

Engineering can improve control but also alter donor-cell cargo, membrane integrity, aggregation, charge, biodistribution, immunogenicity, release kinetics, and apparent dose; these effects depend on the modification method.

Each added component should demonstrate benefit over a simpler comparator; shared CMC, comparability, and chronic-safety requirements are consolidated in Section 8.

Engineered IBD platforms remain mechanistically uneven. PD-L1/miR-27a-3p EVs provide relatively strong receptor- and pathway-dependence evidence, whereas many ligand-, cargo-, and biomaterial-modified systems mainly improve exposure or formulation. Priority should go to designs that experimentally connect the modification, disease-relevant cell engagement, functional signaling, and therapeutic outcome.

## 6 Functional mucosal targeting for immune and regenerative activity

Functional mucosal targeting links regional exposure, disease-relevant cell engagement, cargo delivery or receptor signaling, and a functional response. Because UC and CD differ anatomically and biologically, targeting evidence from acute superficial colitis cannot be generalized across IBD phenotypes.

### 6.1 Disease phenotype and target-cell selection

IBD does not present a single cellular target. Injured epithelium, mucus-associated interfaces, myeloid and lymphoid cells, stromal and vascular compartments, regenerative niches, and microbial signals occupy different tissue regions. The depth, location, cellular composition, and repair capacity of these regions also differ between UC and CD phenotypes (2). Platform design should begin with the anatomical compartment and the cell state relevant to the intended therapeutic effect.

Target choice follows function. Epithelial or stem/progenitor engagement is relevant to barrier restoration and regeneration; macrophage and dendritic-cell engagement to inflammatory resolution or tolerance; and T-cell-state modulation to immune reprogramming. The associated functional endpoint should reflect objective control of inflammation or restoration of mucosal integrity at the clinical level (3). For an EV delivery claim, however, fluorescence or internalization records exposure, not functional intracellular cargo delivery (21).

Human tissue studies reinforce the need for cell-state resolution. Single-cell and spatial analyses have identified distinct myeloid populations and tissue locations in inflamed intestine (84). Longitudinal profiling during anti-TNF therapy has linked treatment response to changes across epithelial, immune, and stromal compartments (85). Multi-omic analysis of vedolizumab-treated UC also implicated mononuclear phagocytes, fibroblasts, and epithelial subsets in treatment-associated remodeling (86). These observations favor phenotype-specific targets over broad colon accumulation.

Validation should identify the engaged disease-relevant cell state and measure the corresponding tolerogenic, myeloid, epithelial-repair, barrier, or regenerative response.

### 6.2 Route selection and compartment-specific delivery

Oral delivery is appropriate for luminal or superficial targets only when gastrointestinal stability is followed by regional distribution, cell engagement, and mechanism-linked activity (39, 40, 42, 67, 87, 88).

Rectal delivery is a phenotype-specific option for distal colonic disease, limited by retention, acceptability, formulation stability, and proximal coverage.

Systemic administration may provide better access to inflamed vasculature, circulating immune cells, lymphoid tissues, and deeper or transmural intestinal compartments, but it also increases exposure in the liver, spleen, lung, kidney, and other non-target organs. In a non-EV comparison study (89), intravenously administered HA-functionalized curcumin nanoparticles showed greater localization to colitic tissue than the corresponding oral formulation. This platform-specific finding illustrates that route selection should be guided by target anatomy, mechanism of action, and disease phenotype rather than by an assumption that intestinal disease necessarily favors oral administration.

Local-retention systems, including hydrogels, bioadhesive polymers, and pH-, ROS-, or enzyme-responsive formulations, may prolong mucosal contact or enable disease-responsive release. Evidence from non-EV intestinal nanomedicine (90) provides a design rationale for these approaches, but vesicle-based formulations must additionally verify vesicle integrity within the carrier, release kinetics, cargo activity, mucosal compatibility, degradation products, sterility, and manufacturability. The retention material should therefore be evaluated as an active component of the final product rather than as an inert delivery accessory.

Route and endpoints should match the intended phenotype and compartment; deep, fistulizing, fibrotic, or postoperative disease requires corresponding exposure and structural outcomes.

### 6.3 Ligand-guided cell engagement and functional targeting

Targeting requires more than accumulation: a modified platform should establish preferential localization, ligand-receptor interaction or receptor dependence, disease-relevant cell engagement, and the intended cargo or surface-signaling response. Inflamed tissue can otherwise increase nonspecific retention.

HA-based non-EV systems targeting CD44 illustrate how colonic localization should be linked to epithelial or macrophage engagement (91, 92); they are design comparators rather than EV efficacy evidence.

HA-modified red-cabbage particles showed greater cell association and efficacy than unmodified particles (11), but absent receptor-dependence testing limits the claim to enhanced engagement.

Other designs use peptides, antibody fragments, nanobodies, aptamers, chemokine motifs, or checkpoint molecules. PD-L1/miR-27a-3p EVs provide pathway-dependence evidence, whereas nanobody/LL37 milk EVs mainly support oral cargo protection (6, 20, 67).

## 7 Integrated immune-regenerative mechanisms

Cross-platform integration in **Table 1** treats model relevance, mechanism, delivery, development stage, and clinical status as independent domains.

### 7.1 Immune resolution and tolerance

Across EV and EV-like platforms, recurring immune-associated themes include myeloid-state modulation, tolerance-associated dendritic-cell states, and coupling between innate and adaptive immunity. Ginger- and turmeric-derived preparations have been associated with reduced inflammatory cytokines and altered macrophage-related states, but the available evidence does not establish macrophages as required mediators (9, 10). These findings are therefore interpreted as changes in the mucosal myeloid environment, including reduced inflammatory activation or increased resolution-associated programs, rather than as uniform conversion between binary polarization states, particularly because human IBD contains heterogeneous macrophage and neutrophil states (84).

More mechanistically informative examples include AMPK dependence and dendritic-cell transfer evidence for broccoli-derived nanoparticles and PD-1/PHB1-related immune regulation for engineered PD-L1/miR-27a-3p EVs. In these specific preparations, primary studies support regulation of antigen-presenting-cell function or T-cell-associated responses together with epithelial protection (6, 8). In the broader IBD context, Th17 responses are shaped by interactions among antigen-presenting-cell activity, cytokine exposure, epithelial-derived signals, and the local metabolic environment (93). These preparation-specific findings do not establish a shared mechanism for all EVs or PELNs.

Tolerance-oriented strategies should balance inflammatory control with antimicrobial defense, epithelial repair, and infection risk.

### 7.2 Barrier restitution and epithelial regeneration

Barrier restoration is closely linked to immune regulation because epithelial disruption promotes microbial translocation and sustains innate inflammatory activation. Accordingly, reductions in cytokines should be interpreted together with functional epithelial endpoints such as permeability, transepithelial electrical resistance, tight-junction integrity, mucus-associated defense, epithelial survival, and wound closure (94).

Several PELN and engineered EV preparations have produced concurrent improvements in inflammatory and barrier-related readouts (9, 11, 44, 67). These findings support coordinated immune–epithelial activity but do not identify a shared barrier mechanism across platforms. Barrier protection should also be distinguished from true regeneration. Restoration of tight-junction proteins or reduced permeability does not necessarily demonstrate activation of stem- or progenitor-cell programs.

The grape-derived nanoparticle study provides stronger regeneration-oriented evidence because lipid-fraction experiments and β-catenin blockade supported Lgr5-associated epithelial renewal (7). Even in this setting, regeneration claims should extend beyond organoid enlargement or crypt expansion. Suitable endpoints include stem/progenitor engagement, epithelial differentiation, controlled proliferation, durable wound repair, and restoration of functional tissue architecture.

Regeneration-oriented evidence extends beyond grape-derived nanoparticles but remains preparation-specific. HucMSC-derived exosomes increased Lgr5 expression, epithelial proliferation, and organoid growth; Wnt-C59 attenuated the therapeutic response, supporting Wnt/β-catenin-dependent repair (95). More recently, iPSC-MSC-derived exosomes preserved Lgr5- and Bmi1-associated stem-cell readouts, while miR-34a-5p gain- and loss-of-function experiments linked the response to PPP2R3A and Wnt/β-catenin signaling (96). Neither study used lineage tracing or demonstrated stable epithelial differentiation and functional tissue architecture after long-term follow-up; accordingly, they strengthen pathway-supported regeneration without closing the evidence-maturity gap relative to immunomodulation and barrier protection.

### 7.3 Regenerative benefit versus proliferative risk

Regenerative activity is therapeutically desirable only when it restores tissue structure without promoting uncontrolled growth. Increased epithelial survival and crypt-cell expansion may support acute healing, but repeated stimulation could potentially favor excessive proliferation, impaired differentiation, or dysplasia-prone signaling. A 2024 integrated human gut atlas identified MUC6-positive inflammatory epithelial metaplastic cells (INFLAREs) that emerge from altered stem-cell states and can recruit T cells and neutrophils, showing that repair-associated metaplasia may also perpetuate inflammation and increase concern about progression toward neoplasia (97).

Regeneration studies should pair mechanism-linked repair assays with differentiation quality, growth control, repeat-dose exposure, and long-term safety. Exploratory disease, cytokine, histology, or organoid endpoints are not validated lot-release potency assays.

### 7.4 Microbiota interaction as an indirect mechanism

Microbiota changes may precede immune regulation, follow reduced inflammation or barrier repair, or participate in a bidirectional response; establishing mediation requires intervention because before-and-after sequencing alone cannot distinguish these directions (9, 10).

Across platforms, evidence supports preparation-specific immune, epithelial, and repair effects, with mechanistic, pharmacodynamic, manufacturing, safety, and human relevance assessed separately.

## 8 From preclinical promise to translational readiness

### 8.1 Evidence maturity and model validity

Most evidence comes from acute DSS or TNBS colitis, which models epithelial injury and innate inflammation but not chronic, relapsing, transmural, fibrotic, fistulizing, or postoperative IBD. Short-term disease and inflammatory outcomes are screening endpoints; translation requires durable healing, relapse-relevant models, repeat-dose safety, and compatibility with existing therapy (98).

Meta-analyses show a consistent anti-inflammatory trend but also heterogeneous sources, doses, quality, and potential publication bias (5, 32). Models should match the biological question: epithelial injury for barrier repair, immune-mediated disease for tolerance, fibrosis models for remodeling, microbiota-dependent models for ecological effects, and human-derived systems for target-cell validation. Randomization, blinding, dose-response testing, appropriate controls, and independent replication remain essential.

Evidence maturity is multidimensional: strong pathway dependence in an acute model and direct human exposure with limited mechanism answer different questions. **Table 1** therefore reports independent domains, with the weakest decision-critical domain defining the next experiment.

#### 8.1.1 Phenotype-specific interpretation

Evidence for transmural, fibrostenotic, or postoperative Crohn’s disease is especially limited. Improvement in epithelial injury or wound closure should not be interpreted as control of mesenchymal activation, extracellular-matrix deposition, stricture progression, or transmural inflammation. Reparative signaling may be beneficial in acute mucosal injury yet require separate testing for pathological stromal remodeling and fibrosis in chronic Crohn-like models (2).

Perianal fistulizing Crohn’s disease is a distinct local indication supported only by a small uncontrolled published feasibility study and early registry programs. Human luminal UC and Crohn’s disease remain represented mainly by small, terminated, or not-yet-recruiting studies without posted results, while patient-derived organoids, mucosal explants, and ex vivo immune-epithelial systems provide human relevance but not clinical efficacy. Conclusions should therefore be stated separately for acute UC-like mucosal injury, chronic or relapsing colitis, transmural or fibrotic Crohn-like disease, perianal fistulizing Crohn’s disease, and human luminal UC or Crohn’s disease.

### 8.2 Product identity, characterization, and dose definition

A major translational barrier is unclear product identity. EVs are not single-molecule drugs; they are heterogeneous biological particles containing variable combinations of lipids, proteins, RNAs, metabolites, and surface molecules. Their characteristics may be affected by donor cell type, culture conditions, passage number, inflammatory priming, isolation method, storage condition, and formulation procedure. For PELNs, additional variability arises from plant species, cultivar, tissue source, growth conditions, harvest timing, extraction method, purification procedure, co-isolated plant materials, and microbial or environmental contamination. Recent quality-control discussions for plant-derived EVs further emphasize that source material preparation, isolation and purification, physicochemical characterization, functional testing, and contaminant control should be treated as interconnected determinants of product identity rather than as optional technical details (99).

This variability directly affects dose definition. Across EV and PELN studies, dose is reported as total protein amount, particle number, RNA quantity, vesicle volume, source material weight, or crude extract equivalent. These units are not interchangeable. A preparation with the same protein dose may contain different particle numbers, vesicle subpopulations, cargo profiles, and biological activity. Conversely, particle number alone does not define active cargo content or therapeutic potency. Therefore, dose should be linked to product identity and biological function rather than treated as a purely quantitative particle metric.

A clinically meaningful vesicle product should define critical quality attributes. For therapeutic EV products, these attributes should connect source material, manufacturing process, vesicle identity, purity, potency, impurities, sterility, endotoxin burden, stability, and safety-related parameters (62, 83). For mammalian EVs, these may include particle size distribution, concentration, morphology, protein markers, purity, cargo profile, sterility, endotoxin level, storage stability, and potency. For PELNs, identity criteria should also include lipid composition, plant-derived RNA and protein cargo, small-molecule content, gastrointestinal stability, co-isolated non-vesicular material, microbial contamination, and batch consistency. MISEV2023 provides a useful foundation for EV nomenclature, sample processing, separation, characterization, uptake, and functional analysis, but PELNs require additional platform-specific identity criteria rather than direct application of mammalian exosome markers (15).

### 8.3 Mechanistic validation, pharmacodynamic readouts, and product-release potency

Mechanistic validation, pharmacodynamic measurement, and product-release potency serve different purposes. Mechanistic experiments test whether a cargo, receptor, pathway, formulation attribute, or cell population contributes to activity. Pharmacodynamic readouts demonstrate a biological response after treatment in cells, tissues, animals, or human samples. A product-release potency assay is a quantitative, reproducible, standardized assay used to compare manufacturing lots and requires a defined analytical range, precision, robustness, acceptance criteria, and evidence linking the assay to the intended biological function. Reduced disease activity, cytokine expression, or histological injury may be useful pharmacodynamic findings, but they are not automatically causal evidence or lot-release assays (62, 100).

For IBD products, assay selection should remain mechanism- and phenotype-informed. Macrophage-directed platforms may require myeloid-state dependency testing and a separate quantitative inflammatory-response assay; dendritic-cell platforms may require antigen-presentation or T-cell-polarization assays; checkpoint products require receptor engagement plus immune-safety measurements; and epithelial-repair products may use permeability, TEER, wound closure, differentiation, or organoid readouts. Engineered products will usually require a matrix of mechanistic, pharmacodynamic, safety, and release assays rather than a single endpoint. Only the analytically validated assay or assay combination should be proposed for routine lot release.

### 8.4 Exposure, target-cell engagement, cargo delivery, and surface signaling

Applying the domains defined in Section 2.3, exposure studies should control for dye leakage, aggregation, labeling effects, and signal persistence, and distinguish intestinal from systemic or lymphoid distribution (15, 21, 66).

Cell-resolved analyses should identify engagement in inflamed and non-inflamed tissue. Cargo-loaded products require cargo stability, intracellular bioavailability, target modulation, and matched free-cargo, unloaded, and mock-loaded controls; surface-engineered products require ligand-receptor dependence and downstream signaling. Off-target distribution should be quantified before late preclinical development.

**Figure 2:**
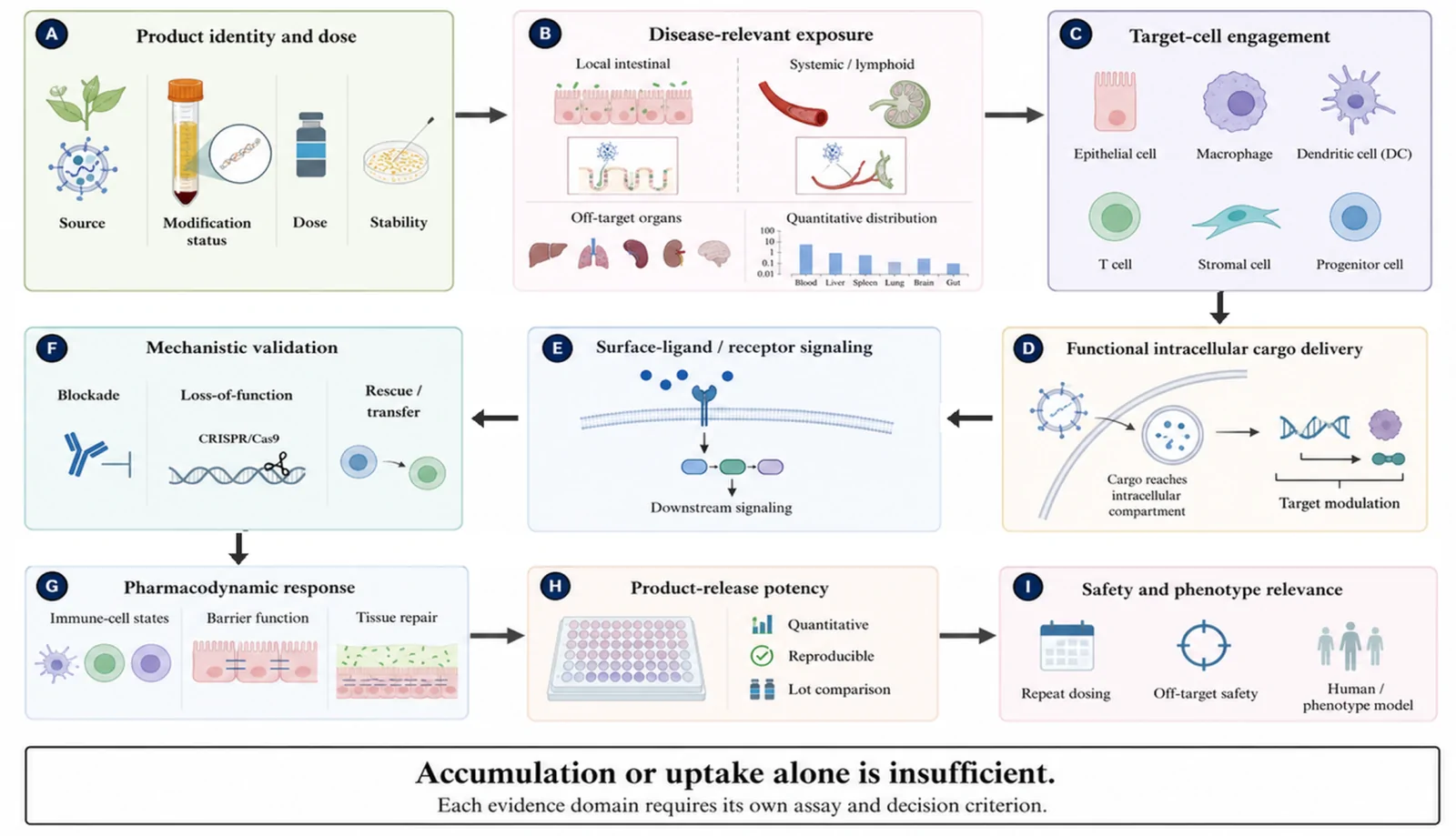
Evidence domains for evaluating EV-based and EV-like products in IBD. **(A)** Product identity and dose; **(B)** disease-relevant exposure; **(C)** target-cell engagement; **(D)** functional intracellular cargo delivery; **(E)** surface-ligand/receptor signaling; **(F)** mechanistic validation; **(G)** pharmacodynamic response; **(H)** product-release potency; and **(I)** safety and phenotype relevance. Each domain requires appropriate assays and decision criteria. Accumulation or uptake alone does not establish functional delivery or receptor signaling. Mechanistic validation, pharmacodynamic responses, and lot-release potency are assessed separately. The domains are complementary and do not prescribe a fixed experimental sequence.

Pharmacodynamic changes cannot substitute for delivery or dependency evidence. Advancement requires the relevant exposure, engagement, functional action, and benefit against an appropriate comparator; **Figure 2** and **Table 2** summarize these gates.

**Table 2:** Translational validation checklist separating exposure, cargo delivery, surface signaling, mechanistic validation, pharmacodynamic response, and lot-release potency.

Validation gate	Minimum claim	Suggested assays/readouts	No-go concern
Product identity and dose	Source, modification status, purity, cargo, stability, and dose metric are reproducible	NTA/TRPS; EM; marker panel; cargo/impurity profiling; stability; dose recovery	Identity, modification status, or dose is not reproducible
Disease-relevant regional or systemic exposure	Exposure is quantified in the intended intestinal, vascular, lymphoid, or systemic compartment with off-target distribution	Regional and organ quantification; inflamed/non-inflamed comparison; label-leakage controls; clearance	A fluorescence signal is interpreted without label and free-dye controls
Target-cell engagement	Relevant epithelial, myeloid, lymphoid, stromal, or progenitor compartments are engaged	Flow cytometry; spatial imaging; cell sorting; ligand/receptor blocking	Organ accumulation is shown but the engaged cell type is unknown
Functional intracellular cargo delivery	Cargo reaches and acts in the relevant intracellular compartment	Cargo tracking; compartment reporters; target modulation; free-cargo and unloaded-particle controls	Internalization is equated with functional cargo delivery
Surface-ligand/receptor signaling	A surface signal engages its receptor and produces downstream signaling	Binding/competition; receptor blockade or knockout; downstream signaling; internalization-independent controls	Receptor signaling is inferred from uptake or cargo activity alone
Mechanistic validation	A defined cargo, receptor, pathway, or cell population contributes to activity	Loss-of-function; blockade; depletion; rescue; transfer; appropriate modified/unmodified comparison	One pathway-associated readout is generalized to the full therapeutic sequence
Pharmacodynamic response	Treatment produces a reproducible biological response in the intended model or compartment	Cytokines; immune-cell states; TEER/permeability; histology; organoid or explant response	A downstream response is mislabeled as causal proof or lot-release potency
Product-release potency	A quantitative, reproducible assay is suitable for lot comparison and linked to intended function	Validated assay range; precision; robustness; reference standard; acceptance criteria; stability indication	An exploratory research assay is used without analytical validation or lot-discrimination evidence
Safety, durability, and repeat dosing	Repeated exposure has acceptable local and systemic safety and preserves immune competence	Immunogenicity; infection response; microbiota; proliferation; off-target organs; withdrawal/retreatment	Immune suppression, dysbiosis, accumulation, or proliferative risk is unacceptable
Human and phenotype relevance	Animal findings are tested in an appropriate human system and matched to the intended IBD phenotype	Patient organoids; immune-epithelial co-cultures; explants; gut-on-chip; phenotype-specific endpoints	Acute rodent mucosal evidence is generalized to all UC/CD phenotypes or late development

CMC, chemistry, manufacturing, and controls; EV, extracellular vesicle; IBD, inflammatory bowel disease; PELN, plant-derived EV-like nanoparticle; TEER, transepithelial electrical resistance.

### 8.5 Manufacturing, regulatory classification, and registered clinical development

Human therapeutic experience remains limited. In the small uncontrolled phase I study of locally injected MSC-derived exosomes for refractory perianal Crohn’s disease fistulas, five patients were treated; four were reported as responders at six months and three achieved complete healing, with no reported local or systemic adverse events (33). These observations provide a preliminary local safety and feasibility signal but do not establish efficacy and cannot be generalized to oral or systemic treatment of luminal IBD.

The updated ClinicalTrials.gov search identified nine participant-treatment records relevant to IBD and one additional ex vivo human-relevance study as of the registry data timestamp on 5 August 2026 (**Supplementary Table S6**). The treatment records include plant- or milk-derived oral products, intravenously administered MSC-EV or EV-enriched secretome products for luminal UC or Crohn’s disease, and locally injected MSC-derived products for perianal fistulizing Crohn’s disease. One record was completed, three were terminated, three were not yet recruiting, one was recruiting, and one had an unknown status because it had not been verified recently. Planned or actual enrollment ranged from 3 to 100 participants.

None of these registry records had posted results or a registry-linked outcome publication at the cutoff, and an exact-NCT-identifier PubMed search returned no indexed records. The ex vivo study exposes patient-derived biopsies, peripheral blood mononuclear cells, and organoid co-cultures to a secretome containing soluble factors and EVs; it may inform human biological relevance but is not a participant-dosing trial. Accordingly, registration, recruitment, completion, or ex vivo activity is reported as development status only and is not counted as evidence of safety, feasibility, or efficacy. The published five-patient perianal-fistula report is retained separately because registry linkage was not assumed without explicit evidence.

Across the broader EV field, clinical development has expanded faster than standardization. A critical review of EV-related clinical trials reported rapid growth in registered studies but found incomplete reporting of isolation and characterization methods and limited attention to EV subpopulations (16). This gap is important for IBD because chronic disease treatment requires reproducible product identity, stable dosing, validated potency assays, and long-term safety monitoring.

Regulatory classification should be determined by product source, modification status, manufacturing process, active cargo, mechanism of action, route, and repeat-dosing plan rather than by the generic designation EV or exosome. Regulatory analyses and development frameworks (17, 62, 101) support a risk-based approach in which unmodified native MSC-EVs, donor-conditioned EVs, milk-derived EVs, post-isolation engineered or cargo-loaded products, and PELNs may follow different development pathways. Introduced nucleic acids, proteins, targeting ligands, or antimicrobial components require additional evaluation of cargo pharmacology, off-target effects, immunogenicity, residual reagents, and process impurities. PELNs require plant-source control, agricultural and microbial controls, compositional consistency, and distinction between dietary exposure and therapeutic dosing. Regulatory requirements should therefore reflect the defining attributes and intended use of each individual product.

Manufacturing readiness is equally important for clinical translation. GMP-oriented EV manufacturing studies (102, 103) emphasize the need for defined starting materials, scalable production, standardized purification and concentration, in-process controls, sterile processing, release specifications, stability testing, and batch comparability. For mammalian EVs, critical variables include donor-cell identity and expansion, culture-medium composition, serum-derived contaminants, priming conditions, purification yield, sterility, endotoxin burden, storage stability, and lot-to-lot consistency. PELN manufacturing additionally requires control of plant species and cultivar, cultivation conditions, harvest timing, extraction procedures, microbial burden, pesticide or environmental residues, and co-isolated plant-derived materials. For engineered or cargo-loaded products, translational guidance (62) indicates that each loading, conjugation, membrane-modification, purification, or formulation step should be incorporated into the quality-control strategy because it may change vesicle integrity, impurity profiles, potency, and reproducibility. Comparability testing should therefore be repeated after material changes in donor-cell culture, cargo-loading procedures, surface-modification chemistry, purification workflows, formulation, storage conditions, or scale-up processes.

A donor-cell process study reported that bafilomycin A1 increased iPSC-MSC EV output and reduced parental-cell reuptake while the recovered EV preparation retained broadly comparable activity in the tested models. This is useful production-process evidence, but it does not by itself establish scalable manufacture, cargo equivalence, batch comparability, or superior therapeutic efficacy (104).

### 8.6 Safety considerations for chronic intestinal disease

Safety evaluation should be tailored to both disease context and platform composition (**Table 3**). Current quality-and-safety guidance for therapeutic EV products (83) highlights the need to assess biological activities that may affect immunity, coagulation, fibrosis, angiogenesis, epithelial proliferation, and tumor-related pathways. Milk-derived EVs and PELNs additionally require evaluation of allergenicity, microbial or environmental contamination, source variability, and compositional uncertainty, as emphasized in recent plant-vesicle quality-control analyses (99). For engineered or cargo-loaded products, regulatory assessments (62, 101) should further address altered biodistribution, off-target gene or protein regulation, immunogenicity of introduced components, residual reagents or free cargo, and process-related impurities. These risks cannot be extrapolated uniformly across unmodified native, donor-conditioned, dietary, plant-derived, and post-isolation engineered vesicle products.

**Table 3:** Platform-specific translational risks, required controls, and conditional development roles for EV-based IBD products.

Development domain	Platform-specific risk	Mitigation/required controls	Potential development role
Unmodified native MSC-EVs	Donor, passage, baseline culture, cargo, and potency variability; unclear active component	Standardized source/culture; cargo fingerprints; dose metrics; target-cell and potency assays	Preclinical reference or comparator platform
Donor-conditioned MSC-EVs	Programming changes yield, cargo, surface signals, impurities, and batch comparability	Define conditioning exposure; compare with unmodified EVs; cargo/impurity profile; stability and safety	Candidate biology-modulated EV platform conditional on successful comparability and safety evaluation
Milk-derived EVs	Food matrix, digestion, processing, allergenicity, source variation, and oral dose uncertainty	Digestion-stability; matrix/allergen controls; source specification; loading and free-cargo controls	Candidate oral carrier or barrier-support adjunct
Minimally processed PELNs	Plant source variation, co-isolated metabolites, residues, microbes, and uncertain biogenesis	Species/tissue/growth documentation; purity/residue and microbial testing; particle-dependence controls	Candidate oral mucosal platform with preparation-specific evidence
Decoction-, heat-, or extraction-derived plant particles	Processing may alter particle identity and enrich non-vesicular phytochemicals	Document processing; compare raw-tissue particles and soluble fractions; composition and activity attribution	Candidate processed botanical particle formulation; EV identity unconfirmed
Post-isolation engineered PELNs	Surface or chemical modification may change biodistribution, immunogenicity, and safety	Modified/unmodified comparison; receptor dependence; off-target distribution; repeat-dose safety	Candidate ligand-guided epithelial or immune-cell engagement
Cargo-engineered mammalian or milk EVs	Residual free cargo, loading heterogeneity, off-target effects, and dose-response uncertainty	Free-cargo, unloaded, and mock-loaded controls; functional-delivery quantification; dose-response	Candidate defined biologic or RNA delivery platform
Checkpoint-displaying EVs	Excessive immune suppression, infection risk, systemic exposure, and T-cell overprogramming	Local/systemic exposure; infection-response monitoring; receptor dependence; durability and repeat dosing	Candidate localized immune-modulation platform conditional on satisfactory systemic-safety evaluation
Biomaterial-retained EV products	Formulation may change vesicle integrity, release kinetics, mucosal compatibility, and manufacturability	Release kinetics; vesicle stability; local toxicity; degradation products; sterility and comparability	Candidate localized exposure or retention strategy

Because IBD treatment may require repeated administration, safety assessment should extend beyond acute tolerability. Regulatory and clinical guidance (101, 102) supports monitoring immunogenicity, systemic exposure, cumulative toxicity, and delayed adverse effects during repeat-dose development. The testing strategy should then follow the intended mechanism: immune-suppressive platforms require monitoring of infection susceptibility and host defense; regenerative products require assessment of epithelial proliferation, differentiation, and colitis-associated cancer risk; cargo-loaded EVs require off-target pharmacology and residual-cargo testing; and microbiota-interacting products require longitudinal evaluation of ecological stability and barrier function. Reproductive toxicity should be considered when clinically relevant, while long-term studies should specifically examine persistent dysbiosis, abnormal tissue proliferation, and tumor risk.

### 8.7 Future roadmap

**Figure 3:**
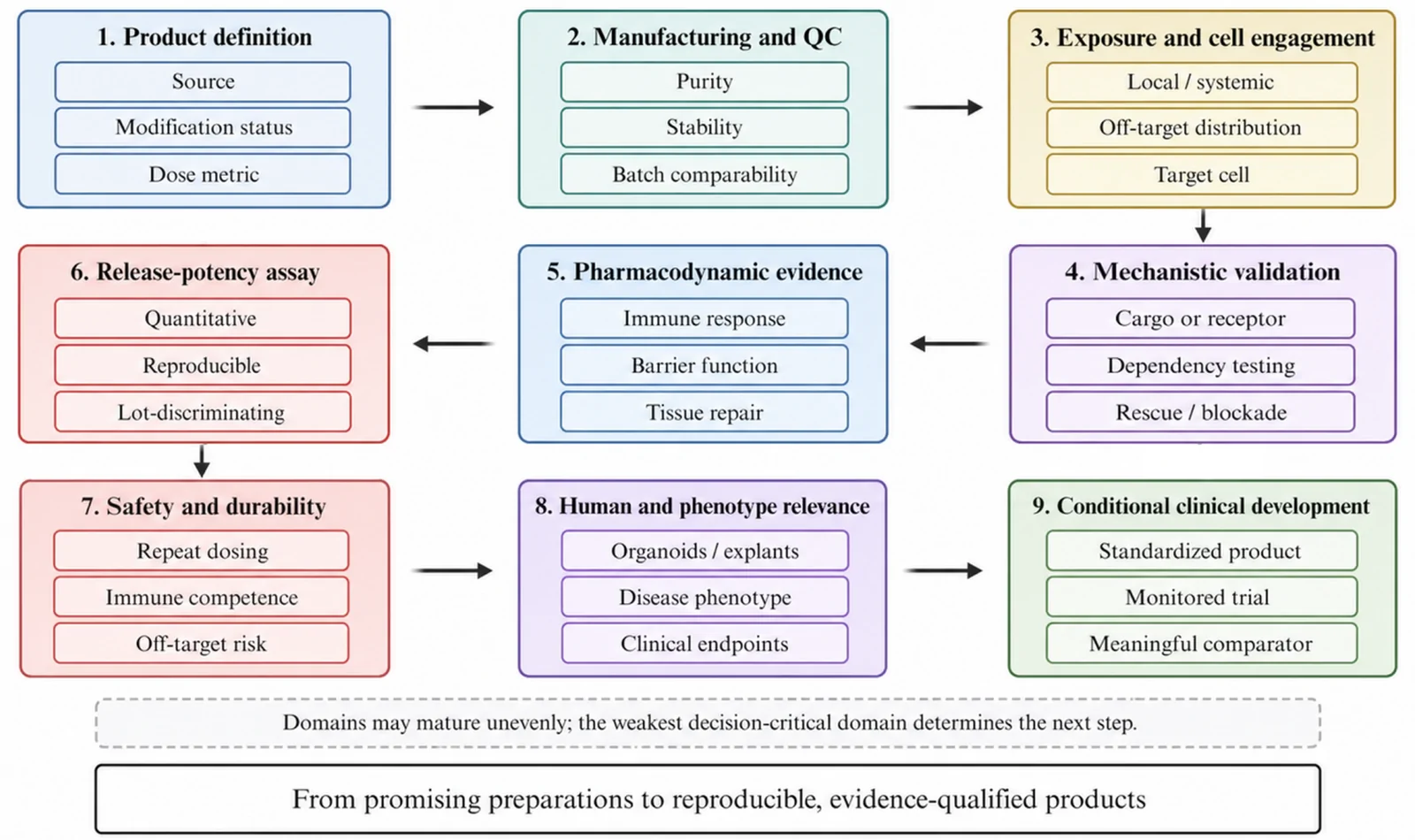
Translational requirements for EV-based and EV-like products in IBD. Product definition and manufacturing control are considered alongside tissue exposure, target-cell engagement, mechanistic validation, pharmacodynamic activity, release-potency testing, safety, durability, and relevance to human disease. Evidence may mature unevenly across these domains; the principal unresolved requirement should guide further development. Clinical progression requires a standardized product, a monitored trial, an appropriate comparator, and a prespecified clinically meaningful benefit. QC, quality control.

Development should align product identity and dose with phenotype, compartment, mechanism, potency, safety, and manufacturing control. Progression requires a prespecified clinical advantage over an appropriate comparator and resolution of each decision-critical gap (**Figure 3**).

## 9 Limitations of this review

This review used a structured, representative rather than exhaustive selection strategy. Study inclusion may therefore remain partly subjective despite prespecified eligibility, prioritization rules, and the supplementary disposition audit. Searches were limited to English-language records, and no formal risk-of-bias or certainty framework was applied. Evidence descriptors map the type and location of evidence; they are not study-quality or certainty ratings.

Direct comparison is further limited by heterogeneity in vesicle sources, donor or plant processing, isolation and purification methods, dose units, routes, treatment schedules, disease models, comparators, and outcome definitions. Many study-level fields remain incompletely reported, and positive findings are dominated by short-term chemically induced rodent colitis. The literature and clinical registry are also evolving rapidly after the 5 August 2026 cutoff. These review-method limitations are separate from, and compound, the experimental and translational limitations of the underlying studies.

## 10 Conclusion

Current evidence supports preparation-specific anti-inflammatory, epithelial-protective, and barrier effects mainly in acute preclinical mucosal-colitis models. It does not establish class-wide efficacy in chronic, fibrostenotic, postoperative, or human luminal IBD; the five-patient fistula study remains a local feasibility signal.

Mechanistic confidence is highest when blockade, loss-of-function, transfer, or comparable interventions test a defined branch. Uptake, cytokine shifts, barrier markers, and microbiota changes otherwise remain associative, and evidence for stem/progenitor-associated regeneration is narrower than evidence for immunomodulation or barrier protection.

Translation requires reproducible identity and dose, phenotype-relevant exposure, mechanism-linked potency, clinically relevant comparators, human systems, and chronic safety. The weakest decision-critical domain should determine progression.

## Glossary

ACSL4: acyl-CoA synthetase long-chain family member 4
ALIX: ALG-2-interacting protein X
AMPK: AMP-activated protein kinase
BM-MSC: bone marrow-derived mesenchymal stromal/stem cell
CAC: colitis-associated cancer
CD: Crohn’s disease
CD4: cluster of differentiation 4
CD9: cluster of differentiation 9
CD44: cluster of differentiation 44
CD63: cluster of differentiation 63
CD81: cluster of differentiation 81
CD163: cluster of differentiation 163
CMC: chemistry, manufacturing, and controls
DAI: disease activity index
DC: dendritic cell
DSS: dextran sulfate sodium
EM: electron microscopy
EV: extracellular vesicle
FOXP3: forkhead box P3
GELN: grape-derived exosome-like nanoparticle
GMP: good manufacturing practice
HA: hyaluronic acid
HO-1: heme oxygenase-1
hUC-MSC: human umbilical cord-derived mesenchymal stromal/stem cell
IBD: inflammatory bowel disease
IL: interleukin
JAK: Janus kinase
Lgr5: leucine-rich repeat-containing G protein-coupled receptor 5
LL37: cathelicidin antimicrobial peptide LL-37
M1/M2: conventional macrophage polarization nomenclature
MISEV2023: Minimal Information for Studies of Extracellular Vesicles 2023
miRNA: microRNA
MMP: matrix metalloproteinase
MPO: myeloperoxidase
MSC: mesenchymal stromal/stem cell
NF-κB: nuclear factor kappa-light-chain-enhancer of activated B cells
NLRP3: NOD-like receptor family pyrin domain-containing protein 3
NTA: nanoparticle tracking analysis
PBMC: peripheral blood mononuclear cell
PD-1: programmed cell death protein 1
PD-L1: programmed death-ligand 1
PDELN: plant-derived exosome-like nanoparticle
PDEV: plant-derived extracellular vesicle
PELN: plant-derived extracellular vesicle-like nanoparticle
PHB1: prohibitin 1
PLGA: poly(lactic-co-glycolic acid)
RNA: ribonucleic acid
ROS: reactive oxygen species
siRNA: small interfering RNA
STAT3: signal transducer and activator of transcription 3
TAT: trans-activator of transcription peptide
TEER: transepithelial electrical resistance
Th17: T helper 17 cell
TLR4: Toll-like receptor 4
TNBS: 2,4,6-trinitrobenzene sulfonic acid
TNF-α: tumor necrosis factor alpha
Treg: regulatory T cell
TRPS: tunable resistive pulse sensing
TSG101: tumor susceptibility gene 101 protein
UC: ulcerative colitis.

## References

[1] Kaplan GG Windsor JW . The four epidemiological stages in the global evolution of inflammatory bowel disease. Nat Rev Gastroenterol Hepatol. (2021) 18:56–66. doi: 10.1038/s41575-020-00360-x 33033392 PMC7542092

[2] Roda G Chien Ng S Kotze PG Argollo M Panaccione R Spinelli A . Crohn’s disease. Nat Rev Dis Primers. (2020) 6:22. doi: 10.1038/s41572-020-0156-2 32242028

[3] Turner D Ricciuto A Lewis A D’Amico F Dhaliwal J Griffiths AM . STRIDE-II: An update on the selecting therapeutic targets in inflammatory bowel disease (STRIDE) initiative of the International Organization for the Study of IBD (IOIBD): Determining therapeutic goals for treat-to-target strategies in IBD. Gastroenterology. (2021) 160:1570–83. doi: 10.1053/j.gastro.2020.12.031 33359090

[4] Kotla NG Rochev Y . IBD disease-modifying therapies: Insights from emerging therapeutics. Trends Mol Med. (2023) 29:241–53. doi: 10.1016/j.molmed.2023.01.001 36720660

[5] Hou J-J Li W-W Wang X-L Ma A-H Qin Y-H . Efficacy of extracellular vesicles as a cell-free therapy in colitis: a systematic review and meta-analysis of animal studies. Front Pharmacol. (2023) 14:1260134. doi: 10.3389/fphar.2023.1260134 37954844 PMC10637393

[6] Oh M-K Park HS Chae D-H Yu A Park JH Heo J . Engineered extracellular vesicles reprogram T cells by targeting PD-1 and PHB1 signaling in inflammatory bowel disease. Sig Transduct Target Ther. (2025) 10:418. doi: 10.1038/s41392-025-02516-0 41444211 PMC12738735

[7] Ju S Mu J Dokland T Zhuang X Wang Q Jiang H . Grape exosome-like nanoparticles induce intestinal stem cells and protect mice from DSS-induced colitis. Mol Ther. (2013) 21:1345–57. doi: 10.1038/mt.2013.64 23752315 PMC3702113

[8] Deng Z Rong Y Teng Y Mu J Zhuang X Tseng M . Broccoli-derived nanoparticle inhibits mouse colitis by activating dendritic cell AMP-activated protein kinase. Mol Ther. (2017) 25:1641–54. doi: 10.1016/j.ymthe.2017.01.025 28274798 PMC5498816

[9] Zhang M Viennois E Prasad M Zhang Y Wang L Zhang Z . Edible ginger-derived nanoparticles: A novel therapeutic approach for the prevention and treatment of inflammatory bowel disease and colitis-associated cancer. Biomaterials. (2016) 101:321–40. doi: 10.1016/j.biomaterials.2016.06.018 27318094 PMC4921206

[10] Gao C Zhou Y Chen Z Li H Xiao Y Hao W . Turmeric-derived nanovesicles as novel nanobiologics for targeted therapy of ulcerative colitis. Theranostics. (2022) 12:5596–614. doi: 10.7150/thno.73650 35910802 PMC9330521

[11] Kang SJ Lee JH Rhee WJ . Engineered plant-derived extracellular vesicles for targeted regulation and treatment of colitis-associated inflammation. Theranostics. (2024) 14:5643–61. doi: 10.7150/thno.97139 39310109 PMC11413791

[12] Li C Zhao L Wang X Wu J Chen H Lu Q . Biogenesis, preparation, characterization, therapeutic mechanisms and safety evaluation of plant-derived exosome-like nanovesicles in the treatment of ulcerative colitis. J Nanobiotechnol. (2026) 24:402. doi: 10.1186/s12951-026-04209-4 41864908 PMC13130446

[13] Tan F Liu W Li T Li L Chen Z Thakur A . Plant-derived extracellular vesicles as tools and targets for inflammatory diseases. J Nanobiotechnol. (2026) 24:397. doi: 10.1186/s12951-026-04294-5 41864926 PMC13126889

[14] Xu Z Xu Y Zhang K Liu Y Liang Q Thakur A . Plant-derived extracellular vesicles (PDEVs) in nanomedicine for human disease and therapeutic modalities. J Nanobiotechnol. (2023) 21:114. doi: 10.1186/s12951-023-01858-7 36978093 PMC10049910

[15] Welsh JA Goberdhan DCI O’Driscoll L Buzas EI Blenkiron C Bussolati B . Minimal information for studies of extracellular vesicles (MISEV2023): From basic to advanced approaches. J Extracellular Vesicle. (2024) 13:e12404. doi: 10.1002/jev2.12404 38326288 PMC10850029

[16] Mizenko RR Feaver M Bozkurt BT Lowe N Nguyen B Huang K . A critical systematic review of extracellular vesicle clinical trials. J Extracellular Vesicle. (2024) 13:e12510. doi: 10.1002/jev2.12510 39330928 PMC11428870

[17] Wang C Tsai T Lee C . Regulation of exosomes as biologic medicines: Regulatory challenges faced in exosome development and manufacturing processes. Clin Trans Sci. (2024) 17:e13904. doi: 10.1111/cts.13904 39115257 PMC11307316

[18] Njoku GC Forkan CP Soltysik FM Nejsum PL Pociot F Yarani R . Unleashing the potential of extracellular vesicles for ulcerative colitis and Crohn’s disease therapy. Bioact Mater. (2025) 45:41–57. doi: 10.1016/j.bioactmat.2024.11.004 39610953 PMC11602541

[19] Van Niel G D’Angelo G Raposo G . Shedding light on the cell biology of extracellular vesicles. Nat Rev Mol Cell Biol. (2018) 19:213–28. doi: 10.1038/nrm.2017.125 29339798

[20] Liu Q Li D Pan X Liang Y . Targeted therapy using engineered extracellular vesicles: Principles and strategies for membrane modification. J Nanobiotechnol. (2023) 21:334. doi: 10.1186/s12951-023-02081-0 37717008 PMC10505332

[21] Bonsergent E Grisard E Buchrieser J Schwartz O Théry C Lavieu G . Quantitative characterization of extracellular vesicle uptake and content delivery within mammalian cells. Nat Commun. (2021) 12:1864. doi: 10.1038/s41467-021-22126-y 33767144 PMC7994380

[22] Clua‐Ferré L Suau R Vañó‐Segarra I Ginés I Serena C Manyé J . Therapeutic potential of mesenchymal stem cell‐derived extracellular vesicles: A focus on inflammatory bowel disease. Clin Trans Med. (2024) 14:e70075. doi: 10.1002/ctm2.70075 39488745 PMC11531661

[23] Din MAU Wan A Chu Y Zhou J Yan Y Xu Z . Therapeutic role of extracellular vesicles from human umbilical cord mesenchymal stem cells and their wide therapeutic implications in inflammatory bowel disease and other inflammatory disorder. Front Med. (2024) 11:1406547. doi: 10.3389/fmed.2024.1406547 39139783 PMC11319305

[24] Cao L Xu H Wang G Liu M Tian D Yuan Z . Extracellular vesicles derived from bone marrow mesenchymal stem cells attenuate dextran sodium sulfate-induced ulcerative colitis by promoting M2 macrophage polarization. Int Immunopharmacol. (2019) 72:264–74. doi: 10.1016/j.intimp.2019.04.020 31005036

[25] Wu B Su S Wang X Li Y Mei Z Lou F . Bone marrow mesenchymal stem cell-derived exosomes alleviate DSS-induced inflammatory bowel disease in mice through inhibiting intestinal epithelial cell pyroptosis via delivery of TSG-6. Front Immunol. (2025) 16:1601591. doi: 10.3389/fimmu.2025.1601591 40661945 PMC12256234

[26] Wang G Yuan J Cai X Xu Z Wang J Ocansey DKW . HucMSC‐exosomes carrying miR‐326 inhibit neddylation to relieve inflammatory bowel disease in mice. Clin Trans Med. (2020) 10:e113. doi: 10.1002/ctm2.113 32564521 PMC7403704

[27] Wei Z Hang S Wiredu Ocansey DK Zhang Z Wang B Zhang X . Human umbilical cord mesenchymal stem cells derived exosome shuttling mir-129-5p attenuates inflammatory bowel disease by inhibiting ferroptosis. J Nanobiotechnol. (2023) 21:188. doi: 10.1186/s12951-023-01951-x 37303049 PMC10259042

[28] Yang R Huang H Cui S Zhou Y Zhang T Zhou Y . IFN-γ promoted exosomes from mesenchymal stem cells to attenuate colitis via miR-125a and miR-125b. Cell Death Dis. (2020) 11:603. doi: 10.1038/s41419-020-02788-0 32733020 PMC7393506

[29] Liu S Cao Y Chen L Nie Q Liu W Zhao Y . Exosomes from IL-33-stimulated macrophages regulate epithelial barrier function to ameliorate TNBS-induced colitis in mice. Cells. (2026) 15:1217. doi: 10.3390/cells15131217 42439691 PMC13359967

[30] Tang S Feng W Li Z Liu X Yang T Wei F . Extracellular vesicles derived from lipopolysaccharide-pretreated periodontal ligament stem cells ameliorate inflammatory responses in experimental colitis via the PI3K/AKT signaling pathway. Int J Nanomed. (2024) 19:11997–2013. doi: 10.2147/IJN.S494321 39583323 PMC11583767

[31] Tong L Hao H Zhang Z Lv Y Liang X Liu Q . Milk-derived extracellular vesicles alleviate ulcerative colitis by regulating the gut immunity and reshaping the gut microbiota. Theranostics. (2021) 11:8570–86. doi: 10.7150/thno.62046 34373759 PMC8344018

[32] Li Y-J Yu Z-Y Zhang D Zhang F-R Zhang D-M Chen M . Extracellular vesicles for the treatment of ulcerative colitis: A systematic review and meta-analysis of animal studies. Heliyon. (2024) 10:e36890. doi: 10.1016/j.heliyon.2024.e36890 39281542 PMC11400994

[33] Nazari H Alborzi F Heirani-Tabasi A Hadizadeh A Asbagh RA Behboudi B . Evaluating the safety and efficacy of mesenchymal stem cell-derived exosomes for treatment of refractory perianal fistula in IBD patients: Clinical trial phase I. Gastroenterol Rep. (2022) 10:goac075. doi: 10.1093/gastro/goac075 36518984 PMC9733972

[34] Tolomeo AM Castagliuolo I Piccoli M Grassi M Magarotto F De Lazzari G . Extracellular vesicles secreted by mesenchymal stromal cells exert opposite effects to their cells of origin in murine sodium dextran sulfate-induced colitis. Front Immunol. (2021) 12:627605. doi: 10.3389/fimmu.2021.627605 33927713 PMC8076641

[35] Seegobin N Taub M Vignal C Waxin C Chris V Awad A . Small milk-derived extracellular vesicles: Suitable vehicles for oral drug delivery? Eur J Pharm Biopharm. (2025) 212:114744. doi: 10.1016/j.ejpb.2025.114744 40355010

[36] Zhang W Dong W Cheng C Zhao H Liu Y Zaydel K . Macrophages internalize epithelial‐derived extracellular vesicles that contain ferritin via the macrophage scavenger receptor 1 to promote inflammatory bowel disease. J Extracell Vesicles. (2025) 14:e70105. doi: 10.1002/jev2.70105 40545965 PMC12183398

[37] Hu X Wang J Guo M Guan C Wang Y Wang J . Meta-analysis of plant-derived exosome-like nanoparticles for the treatment of ulcerative colitis: Efficacy and mechanisms insights. Front Pharmacol. (2026) 17:1845981. doi: 10.3389/fphar.2026.1845981 42440456 PMC13333397

[38] Ning H Huang X Deng N Lin X Zheng L Zhu Y . Plant-derived exosome-like nanovesicles: A novel strategy for targeted oral therapy in ulcerative colitis. Int J Nanomed. (2025) 20:10595–611. doi: 10.2147/IJN.S536056 40927660 PMC12415109

[39] Hu S Zhao R Xu Y Gu Z Zhu B Hu J . Orally-administered nanomedicine systems targeting colon inflammation for the treatment of inflammatory bowel disease: Latest advances. J Mater Chem B. (2024) 12:13–38. doi: 10.1039/D3TB02302H 38018424

[40] Zhang L Zubair M Chu Y . Oral plant-derived exosome-like nanovesicles: A new therapeutic perspective for intestinal diseases. Front Pharmacol. (2026) 17:1827379. doi: 10.3389/fphar.2026.1827379 42292798 PMC13260049

[41] Xu C Xu H Zhu Z Shi X Xiao B . Recent advances in mucus-penetrating nanomedicines for oral treatment of colonic diseases. Expert Opin Drug Delivery. (2023) 20:1371–85. doi: 10.1080/17425247.2023.2242266 37498079

[42] Ensign LM Cone R Hanes J . Oral drug delivery with polymeric nanoparticles: The gastrointestinal mucus barriers. Adv Drug Delivery Rev. (2012) 64:557–70. doi: 10.1016/j.addr.2011.12.009 22212900 PMC3322271

[43] Mu J Zhuang X Wang Q Jiang H Deng Z Wang B . Interspecies communication between plant and mouse gut host cells through edible plant derived exosome‐like nanoparticles. Mol Nutr Food Res. (2014) 58:1561–73. doi: 10.1002/mnfr.201300729 24842810 PMC4851829

[44] Liu C Yan X Zhang Y Yang M Ma Y Zhang Y . Oral administration of turmeric-derived exosome-like nanovesicles with anti-inflammatory and pro-resolving bioactions for murine colitis therapy. J Nanobiotechnol. (2022) 20:206. doi: 10.1186/s12951-022-01421-w 35488343 PMC9052603

[45] Rescigno M Di Sabatino A . Dendritic cells in intestinal homeostasis and disease. J Clin Invest. (2009) 119:2441–50. doi: 10.1172/JCI39134 19729841 PMC2735931

[46] Stagg AJ . Intestinal dendritic cells in health and gut inflammation. Front Immunol. (2018) 9:2883. doi: 10.3389/fimmu.2018.02883 30574151 PMC6291504

[47] Na YR Stakenborg M Seok SH Matteoli G . Macrophages in intestinal inflammation and resolution: A potential therapeutic target in IBD. Nat Rev Gastroenterol Hepatol. (2019) 16:531–43. doi: 10.1038/s41575-019-0172-4 31312042

[48] Zhang K Guo J Yan W Xu L . Macrophage polarization in inflammatory bowel disease. Cell Commun Signal. (2023) 21:367. doi: 10.1186/s12964-023-01386-9 38129886 PMC10734116

[49] Barker N Van Es JH Kuipers J Kujala P Van Den Born M Cozijnsen M . Identification of stem cells in small intestine and colon by marker gene Lgr5. Nature. (2007) 449:1003–7. doi: 10.1038/nature06196 17934449

[50] Sato T Vries RG Snippert HJ Van De Wetering M Barker N Stange DE . Single Lgr5 stem cells build crypt-villus structures *in vitro* without a mesenchymal niche. Nature. (2009) 459:262–5. doi: 10.1038/nature07935 19329995

[51] Hammerhøj A Chakravarti D Sato T Jensen K Nielsen O . Organoids as regenerative medicine for inflammatory bowel disease. iScience. (2024) 27:110118. doi: 10.1016/j.isci.2024.110118 38947526 PMC11214415

[52] Shan Y Lee M Chang E . The gut microbiome and inflammatory bowel diseases. Annu Rev Med. (2022) 73:455–68. doi: 10.1146/annurev-med-042320-021020 34555295 PMC10012812

[53] Stolfi C Maresca C Monteleone G Laudisi F . Implication of intestinal barrier dysfunction in gut dysbiosis and diseases. Biomedicines. (2022) 10:289. doi: 10.3390/biomedicines10020289 35203499 PMC8869546

[54] Gao B Huang X Fu J Chen L Deng Z Wang S . Oral administration of Momordica charantia-derived extracellular vesicles alleviates ulcerative colitis through comprehensive renovation of the intestinal microenvironment. J Nanobiotechnol. (2025) 23:261. doi: 10.1186/s12951-025-03346-6 40170075 PMC11959773

[55] Shi R Tan W Jin H Chan S Li W Lei S . MicroRNA-enriched plant-derived exosomes alleviate colitis by modulating systemic immunity, metabolic homeostasis, and gut microbiota. Adv Sci. (2025) 12:e05921. doi: 10.1002/advs.202505921 40994128 PMC12622485

[56] Liu C-X Han Y-J Zhao N Wang Q-N Wan R-R Cao T-T . Vesicle-like nanoparticles extracted from pueraria lobata decoction alleviate colitis by modulating the intestinal microbiota. Extracell Vesicles Circ Nucl Acids. (2026) 7:234–58. doi: 10.20517/evcna.2025.134 41983055 PMC13074279

[57] Wu H Pang M-M Li Y-L Huang J-J Geng S-Z Hong J-H . Flos sophorae immaturus exosome-like nanovesicles alleviate ulcerative colitis by attenuating intestinal oxidative stress and inflammation through activating aryl hydrocarbon receptor via gut microbiota and tryptophan metabolism regulation. J Nanobiotechnol. (2026) 24:132. doi: 10.1186/s12951-026-04083-0 41639673 PMC12879441

[58] Li D Du L Yi G Liang T Midgley A Shu G . Extremophyte-derived exosome-like nanovesicles remodel intestinal barrier dysfunction by multi-dimensional intervention: Physical barrier repair with immune homeostasis and microbiome regulation. J Nanobiotechnology. (2026). doi: 10.1186/s12951-026-04800-9 42432695 PMC13637334

[59] Hou L Cao J Gao S Wang X Zhang Z Li M . Thermally induced reassembly of ginger extracellular vesicles for oral therapy of intestinal inflammation. Res (Wash D C). (2026) 9:1377. doi: 10.34133/research.1377 42548959 PMC13429916

[60] Yue Y-Q Ai Z-L Li L Sheng Y-L Xu N Zhang L . Magnolia biondii-derived exosome-like nanoparticles ameliorate ulcerative colitis by suppressing inflammation, reducing oxidative stress, and modulating gut microbiota. Int J Nanomedicine. (2026) 21:605192. doi: 10.2147/IJN.S605192 42544276 PMC13429129

[61] Zhong H Liu Z Zhao Z Yao S Ning D Qiao H . PO-EVLPs alleviate inflammatory bowel disease and colitis-associated colorectal cancer by delivering miR159-y and suppressing TGFBR2 expression, thereby modulating the phenotypic plasticity of innate lymphoid cells. Acta Pharm Sin B. (2026). doi: 10.1016/j.apsb.2026.07.04442574925

[62] Xu G Jin J Fu Z Wang G Lei X Xu J . Extracellular vesicle-based drug overview: Research landscape, quality control and nonclinical evaluation strategies. Sig Transduct Target Ther. (2025) 10:255. doi: 10.1038/s41392-025-02312-w 40804047 PMC12350758

[63] Li Z Sun Y Wu J Zhang W Li T Tang S . Mesenchymal stem cells derived extracellular vesicles in inflammatory bowel disease: Therapeutic efficacy and bioengineering applications. Int J Nanomed. (2026) 21:584494. doi: 10.2147/IJN.S584494 41993781 PMC13082256

[64] Liu J Ren H Zhang C Li J Qiu Q Zhang N . Orally‐delivered, cytokine‐engineered extracellular vesicles for targeted treatment of inflammatory bowel disease. Small. (2023) 19:2304023. doi: 10.1002/smll.202304023 37728188

[65] Cao J Luo R Miao R Li W Zhu B Yu L . Engineered exosome nanovesicles for delivery of antibodies to treat inflammatory bowel disease. Nat Commun. (2026) 17:2737. doi: 10.1038/s41467-026-69382-4 41688436 PMC13013668

[66] Hagedorn L Jürgens D Merkel O Winkeljann B . Endosomal escape mechanisms of extracellular vesicle-based drug carriers: lessons for lipid nanoparticle design. Extracell Vesicles Circ Nucleic Acids. (2024) 5:344–57. doi: 10.20517/evcna.2024.19 39697635 PMC11648457

[67] Jing R Zhang L Li R Yang Z Song J Wang Q . Milk‐derived extracellular vesicles functionalized with anti‐tumour necrosis factor‐α nanobody and anti‐microbial peptide alleviate ulcerative colitis in mice. J Extracellular Vesicle. (2024) 13:e12462. doi: 10.1002/jev2.12462 38840457 PMC11154809

[68] Han G Kim H Jang H Kim E Kim S Yang Y . Oral TNF-α siRNA delivery via milk-derived exosomes for effective treatment of inflammatory bowel disease. Bioact Mater. (2024) 34:138–49. doi: 10.1016/j.bioactmat.2023.12.010 38223538 PMC10784143

[69] Lee Y Yoo C Kim K-M Kim S Oh Y Cho S . Immunoregulatory protein-hybrid extracellular vesicles via self-loadable backbone cyclization for oral inflammatory bowel disease therapy. Bioact Mater. (2026) 58:70–88. doi: 10.1016/j.bioactmat.2025.11.036 41438203 PMC12720103

[70] Sharpe A Pauken K . The diverse functions of the PD1 inhibitory pathway. Nat Rev Immunol. (2018) 18:153–67. doi: 10.1038/nri.2017.108 28990585

[71] Gan J Sun L Chen G Ma W Zhao Y Sun L . Mesenchymal stem cell exosomes encapsulated oral microcapsules for acute colitis treatment. Adv Healthcare Mater. (2022) 11:2201105. doi: 10.1002/adhm.202201105 35737997

[72] Nie M Huang D Chen G Zhao Y Sun L . Bioadhesive microcarriers encapsulated with IL‐27 high expressive MSC extracellular vesicles for inflammatory bowel disease treatment. Adv Sci. (2023) 10:2303349. doi: 10.1002/advs.202303349 37759399 PMC10646269

[73] Wan S Wang K Huang P Guo X Liu W Li Y . Mechanoelectronic stimulation of autologous extracellular vesicle biosynthesis implant for gut microbiota modulation. Nat Commun. (2024) 15:3343. doi: 10.1038/s41467-024-47710-w 38637580 PMC11026491

[74] Cho C Roh J Lee S Cho H Lee J So G . Orally delivered, pH-resistant dual coated extracellular vesicles restore intestinal barrier function and suppress colitis. Biomaterials. (2026) 331:124146. doi: 10.1016/j.biomaterials.2026.124146 41865564

[75] Mairal A Tariq U Mehrotra S Matheshwaran S Marsal J Kumar A . Probiotic-derived vesicles rectally delivered via thermo-gelling copolymer promote mucosal healing and reduce inflammation in ulcerative colitis. Adv Sci (Weinh). (2026):e76585. doi: 10.1002/advs.76585 42468020 PMC13379261

[76] Belaid M Chng W Muthuramalingam R Lim Y Javorovic J Zhang Y . Oral delivery of mesenchymal stem cell-derived extracellular vesicles to treat intestinal inflammation. ACS Appl Mater Interfaces. (2026) 18:38483–501. doi: 10.1021/acsami.6c07496 42376871 PMC13422774

[77] Zhao M Hou J Wang J Zhu X Zou A Wang Y . Food-derived biohybrid probiotic extracellular vesicles for synergistic therapy of inflammatory bowel disease. Small. (2026) 22:e73858. doi: 10.1002/smll.73858 42160026 PMC13351475

[78] You X Ma J Zou L Shu Y Jia Z Kang Y . Restoring immunoglobulin a/polymeric immunoglobulin receptor transport axis for the oral treatment of inflammatory bowel disease. J Control Release. (2026) 395:115002. doi: 10.1016/j.jconrel.2026.115002 42107777

[79] Liu Y Huang Y Zhang M Zhang Y Zhu C Xia S . Parabacteroides distasonis-derived extracellular vesicles alleviate ulcerative colitis by regulating tryptophan metabolism to activates AhR. Microbiol Res. (2026) 309:128535. doi: 10.1016/j.micres.2026.128535 42034038

[80] Xi Y Zou J Chen L Ma X Yan Y Liang D . Callicarpa nudiflora-derived extracellular vesicle-like particles loaded with budesonide enhance the treatment of ulcerative colitis by regulating M1/M2 macrophage polarization. Biomater Adv. (2026) 184:214834. doi: 10.1016/j.bioadv.2026.214834 41865696

[81] Hou W Sheng K Gao M Liu B Wang H Li X . Bletilla striata-derived extracellular vesicles armed probiotic by supramolecular assembly ameliorates colitis via macrophage modulation and microbiota restoration. J Nanobiotechnol. (2026). doi: 10.1186/s12951-026-04866-538164791

[82] Zou L Jia Z Shu Y You X Ma J Wang Y . Extracellular vesicles from pasteurized akkermansia muciniphila ameliorate inflammatory bowel disease through suppression of STING-driven inflammatory signaling. Front Microbiol. (2026) 17:1876636. doi: 10.3389/fmicb.2026.1876636 42558378 PMC13437525

[83] Takakura Y Hanayama R Akiyoshi K Futaki S Hida K Ichiki T . Quality and safety considerations for therapeutic products based on extracellular vesicles. Pharm Res. (2024) 41:1573–94. doi: 10.1007/s11095-024-03757-4 39112776 PMC11362369

[84] Garrido-Trigo A Corraliza A Veny M Dotti I Melón-Ardanaz E Rill A . Macrophage and neutrophil heterogeneity at single-cell spatial resolution in human inflammatory bowel disease. Nat Commun. (2023) 14:4506. doi: 10.1038/s41467-023-40156-6 37495570 PMC10372067

[85] Thomas T Friedrich M Rich-Griffin C Pohin M Agarwal D Pakpoor J . A longitudinal single-cell atlas of anti-tumour necrosis factor treatment in inflammatory bowel disease. Nat Immunol. (2024) 25:2152–65. doi: 10.1038/s41590-024-01994-8 39438660 PMC11519010

[86] Mennillo E Kim Y Lee G Rusu I Patel R Dorman L . Single-cell and spatial multi-omics highlight effects of anti-integrin therapy across cellular compartments in ulcerative colitis. Nat Commun. (2024) 15:1493. doi: 10.1038/s41467-024-45665-6 38374043 PMC10876948

[87] Yang C Sharma K Mow R Bolay E Srinivasan A Merlin D . Unleashing the potential of oral deliverable nanomedicine in the treatment of inflammatory bowel disease. Cell Mol Gastroenterol Hepatol. (2024) 18:101333. doi: 10.1016/j.jcmgh.2024.03.005 38490294 PMC11176790

[88] Ribeiro A Nunes R . Selective nanoparticulate systems for drug delivery in inflammatory bowel disease. Pharmaceutics. (2025) 18:55. doi: 10.3390/pharmaceutics18010055 41599162 PMC12844886

[89] Liu J Yang C Merlin D Xiao B . Hyaluronic acid-functionalized nanoparticles for ulcerative colitis-targeted therapy: A comparative study of oral administration and intravenous injection. Biomater Sci. (2024) 12:5834–44. doi: 10.1039/D4BM00898G 39415593

[90] Lei F Zeng F Yu X Deng Y Zhang Z Xu M . Oral hydrogel nanoemulsion co-delivery system treats inflammatory bowel disease via anti-inflammatory and promoting intestinal mucosa repair. J Nanobiotechnol. (2023) 21:275. doi: 10.1186/s12951-023-02045-4 37596598 PMC10436423

[91] Xiao B Zhang Z Viennois E Kang Y Zhang M Han M . Combination therapy for ulcerative colitis: Orally targeted nanoparticles prevent mucosal damage and relieve inflammation. Theranostics. (2016) 6:2250–66. doi: 10.7150/thno.15710 27924161 PMC5135446

[92] Hlaing S Cao J Lee J Kim J Saparbayeva A Kwak D . Hyaluronic acid-conjugated PLGA nanoparticles alleviate ulcerative colitis via CD44-mediated dual targeting to inflamed colitis tissue and macrophages. Pharmaceutics. (2022) 14:2118. doi: 10.3390/pharmaceutics14102118 36297553 PMC9612393

[93] Wen Y Wang H Tian D Wang G . TH17 cell: a double-edged sword in the development of inflammatory bowel disease. Therap Adv Gastroenterol. (2024) 17:17562848241230896. doi: 10.1177/17562848241230896 38390028 PMC10883129

[94] Kong C Yang M Yue N Zhang Y Tian C Wei D . Restore intestinal barrier integrity: An approach for inflammatory bowel disease therapy. JIR. (2024) 17:5389–413. doi: 10.2147/JIR.S470520 39161679 PMC11330754

[95] Liang X Li C Song J Liu A Wang C Wang W . HucMSC-exo promote mucosal healing in experimental colitis by accelerating intestinal stem cells and epithelium regeneration via wnt signaling pathway. Int J Nanomed. (2023) 18:2799–818. doi: 10.2147/IJN.S402179 37256205 PMC10226545

[96] Feng T Qiu Y Chen B Huang X Feng R He Y . Mesenchymal stem cell–derived exosomes derived from induced pluripotent stem cells ameliorate inflammation and promote mucosal healing via miR-34a-5p in crohn disease. Inflammatory Bowel Dis. (2026) 32:852–68. doi: 10.1093/ibd/izag014 41773922

[97] Oliver A Huang N Bartolome-Casado R Li R Koplev S Nilsen H . Single-cell integration reveals metaplasia in inflammatory gut diseases. Nature. (2024) 635:699–707. doi: 10.1038/s41586-024-07571-1 39567783 PMC11578898

[98] Kobayashi T Siegmund B Le Berre C Wei S Ferrante M Shen B . Ulcerative colitis. Nat Rev Dis Primers. (2020) 6:74. doi: 10.1038/s41572-020-0205-x 32913180

[99] Shu F Hu Y Sarsaiya S Jin L Yang X Liu F . Plant-derived extracellular vesicles quality control: Key process progress and future research directions. Discover Nano. (2025) 20:233. doi: 10.1186/s11671-025-04399-0 41400892 PMC12708502

[100] Li D Tang Q Yang M Xu H Zhu M Zhang Y . Plant-derived exosomal nanoparticles: potential therapeutic for inflammatory bowel disease. Nanoscale Adv. (2023) 5:3575–88. doi: 10.1039/D3NA00093A 37441251 PMC10334410

[101] Li Q Li Y Shao J Sun J Hu L Yun X . Exploring regulatory frameworks for exosome therapy: Insights and perspectives. Health Care Sci. (2025) 4:299–309. doi: 10.1002/hcs2.70028 40861511 PMC12371722

[102] Terai S Asonuma M Hoshino A Kino-oka M Ochiya T Okada K . Guidance on the clinical application of extracellular vesicles. Regenerative Ther. (2025) 29:43–50. doi: 10.1016/j.reth.2025.02.008 40124467 PMC11930518

[103] Humbert C Cordier C Drut I Hamrick M Wong J Bellamy V . GMP‐compliant process for the manufacturing of an extracellular vesicles‐enriched secretome product derived from cardiovascular progenitor cells suitable for a phase I clinical trial. J Extracellular Vesicle. (2025) 14:e70145. doi: 10.1002/jev2.70145 40831309 PMC12365392

[104] Xiao Y Yuan W Chen S Zhang G Fu Q Cai Z . Bafilomycin A1 potentially enhances mesenchymal stem cell-derived extracellular vesicle production in iPSC-MSC cultures. Discov Med. (2025) 37:1651. doi: 10.24976/discov.med.202537199.144

