# Supplementary material for: Extracellular vesicle-based therapies in IBD: immunomodulation, regeneration, and translation
Source: Front Immunol. 2026 Sep 7;17:1943448. doi: 10.3389/fimmu.2026.1943448 (PMC13591779; doi:10.3389/fimmu.2026.1943448)
Supplement: Supplementary file 1 [file Table1.docx]

**Supplementary Methods: Literature Search and Evidence Selection**

Extracellular Vesicle-Based Therapies in IBD: Immunomodulation, Regeneration, and Translation

These tables document the structured narrative-review search updated on 5 August 2026. PubMed was the primary biomedical search and screening denominator; OpenAlex was an openly accessible title-and-abstract and citation-tracing supplement. Crossref was used only to verify DOI, title, publication type, and publication-date metadata. The four concept modules overlap, so their counts are not additive and do not represent a PRISMA screening denominator. Negative and counter-evidence was intentionally retained when it directly tested product instability, absence of therapeutic benefit, or source/cargo-dependent harm.

# Table S1. Search concept blocks

| **Block** | **PubMed fielded syntax** | **Purpose** |
| --- | --- | --- |
| D - intestinal disease | ("inflammatory bowel disease"[Title/Abstract] OR "ulcerative colitis"[Title/Abstract] OR "Crohn disease"[Title/Abstract] OR colitis[Title/Abstract]) | Captures UC, CD, IBD, and experimental colitis records. |
| V - vesicle platform | ("extracellular vesicle"[Title/Abstract] OR "extracellular vesicles"[Title/Abstract] OR exosome*[Title/Abstract] OR "small extracellular vesicle"[Title/Abstract] OR "small extracellular vesicles"[Title/Abstract] OR "milk-derived extracellular vesicle"[Title/Abstract] OR "plant-derived extracellular vesicle"[Title/Abstract] OR "plant-derived exosome-like nanoparticle"[Title/Abstract] OR PELN*[Title/Abstract] OR PDEV*[Title/Abstract]) | Captures mammalian EVs, milk EVs, engineered EVs, and plant-derived EV-like platforms. |
| E - engineering/delivery | (engineer*[Title/Abstract] OR target*[Title/Abstract] OR "cargo loading"[Title/Abstract] OR "surface modification"[Title/Abstract] OR "oral delivery"[Title/Abstract] OR "mucosal delivery"[Title/Abstract] OR hybrid[Title/Abstract] OR hydrogel[Title/Abstract] OR microcarrier*[Title/Abstract]) | Identifies engineered, targeted, hybrid, and biomaterial-assisted platforms. |
| M - immune-regenerative mechanism | (macrophage*[Title/Abstract] OR "dendritic cell"[Title/Abstract] OR "dendritic cells"[Title/Abstract] OR Treg[Title/Abstract] OR Th17[Title/Abstract] OR epithelial[Title/Abstract] OR barrier[Title/Abstract] OR organoid*[Title/Abstract] OR microbiota[Title/Abstract] OR regeneration[Title/Abstract]) | Captures immune, epithelial, organoid, barrier, microbiota, and repair evidence. |
| T - translation/quality | (potency[Title/Abstract] OR "quality control"[Title/Abstract] OR manufacturing[Title/Abstract] OR GMP[Title/Abstract] OR regulatory[Title/Abstract] OR safety[Title/Abstract] OR biodistribution[Title/Abstract] OR "functional delivery"[Title/Abstract]) | Captures potency, CMC, safety, biodistribution, and regulatory literature. |
| F - fixed limits | ("0001/01/01"[Date - Publication] : "2026/08/05"[Date - Publication]) AND english[Language] | Fixes the publication cutoff at 5 August 2026 and the English-language boundary. |

**Abbreviations:** CMC, chemistry, manufacturing, and controls; GMP, good manufacturing practice.

# Table S2. Search modules and database implementation

| **Source/module** | **Expression or adaptation** | **Role** | **Audit result** |
| --- | --- | --- | --- |
| PubMed Q1 | D AND V AND F | Foundational EV/PELN evidence in IBD or colitis | 617 records; update interval 16 June-5 August 2026: 29 |
| PubMed Q2 | D AND V AND E AND F | Engineering, targeting, oral delivery, hybrids, and retention | 304 records; update interval: 23 |
| PubMed Q3 | D AND V AND M AND F | Immune, barrier, microbiota, organoid, and repair mechanisms | 420 records; update interval: 25 |
| PubMed Q4 | D AND V AND T AND F | Functional delivery, potency, manufacturing, safety, and regulation | 117 records; update interval: 5 |
| OpenAlex OA1-OA4 | OpenAlex title_and_abstract.search modules OA1=D AND V; OA2=D AND V AND E; OA3=D AND V AND M; OA4=D AND V AND T. PubMed field tags were removed, truncated terms were expanded, and filters were language:en and to_publication_date:2026-08-05. | Open cross-disciplinary discovery plus backward/forward citation tracing; records deduplicated against PubMed by PMID, DOI, normalized title, and manual comparison. | OA1-OA4: 942, 451, 644, 204 records; update interval: 25, 19, 22, 7. Three additional eligible original studies after deduplication. |
| Crossref metadata verification | Crossref REST API /works/{doi} lookups for newly identified records. | Verification of DOI, title, journal-article status, and online/publication date only; not an independent topical-search denominator. | Three OpenAlex-only eligible records verified: 10.3389/fmicb.2026.1876636; 10.1186/s12951-026-04866-5; 10.1016/j.apsb.2026.07.044. |
| Google Scholar | Targeted phrase combinations such as "inflammatory bowel disease" "engineered extracellular vesicles", "plant-derived exosome-like nanoparticles" colitis, "milk extracellular vesicle" gastrointestinal stability, and "pathogenic extracellular vesicles" colitis, together with citation searches for included papers. | Online-first discovery, backward/forward citation chasing, and targeted identification of negative or counter-evidence | Not treated as an exhaustive database denominator |
| ClinicalTrials.gov | (inflammatory bowel disease OR ulcerative colitis OR Crohn disease) AND (extracellular vesicle OR extracellular vesicles OR exosome OR exosomes) | Identifies registered interventional programs; registry status is separated from published efficacy | Records manually checked for disease and intervention relevance; last searched 5 August 2026 |

# Table S3. Eligibility and representative-evidence rules

| **Domain** | **Operational rule** | **Reason** |
| --- | --- | --- |
| Disease relevance | Include human IBD, UC, CD, or experimental colitis with an explicit intestinal therapeutic or delivery question. | Prevents extrapolation from unrelated inflammatory or cancer models. |
| Negative and counter-evidence | Include studies that directly test product instability, lack of therapeutic benefit, or source/cargo-dependent harm in an intestinal setting. Such studies may enter Table 1 when they evaluate an otherwise eligible therapeutic platform; non-interventional pathogenic EV studies remain context-only. | Reduces positive-outcome selection bias and identifies preparation-specific failure modes. |
| Eligible platforms | Mammalian EVs, milk-derived EVs, PELNs, engineered/hybrid biological vesicles, and vesicle-biomaterial formulations. | Matches the platform taxonomy of the review. |
| Context-only evidence | Reviews, guidance, regulatory analyses, GMP/manufacturing papers, non-EV delivery systems used as direct design comparators, and selected disease-derived or pathogenic EV studies used solely as mechanistic counter-evidence. | Supports terminology, translation, and risk interpretation without counting context-only studies as therapeutic efficacy. |
| Primary exclusions | Diagnostic biomarker studies; disease-derived EVs without intervention except selected context-only counter-evidence; non-intestinal studies; conference abstracts; non-vesicular nanoparticles without an EV component. | Keeps the therapeutic evidence map aligned with the review question while allowing limited discussion of source- or cargo-dependent harm. |
| Bacterial vesicles | Include probiotic, commensal, or deliberately engineered bacterial vesicles when evaluated as an intestinal therapeutic intervention; retain pathogenic bacterial or fungal EV studies as context-only counter-evidence unless an intervention is tested. | Addresses the emerging therapeutic evidence while preserving the distinct biogenesis, innate-immune/endotoxin safety profile, manufacturing controls, and regulatory pathway of bacterial-vesicle products. |
| Representative selection | Prioritize direct IBD relevance, distinct engineering strategy, mechanistic depth, functional delivery/receptor dependence, human relevance, translational information, and informative null or harmful findings; avoid exhaustive listing of near-duplicate examples. | Makes the narrative selection rule explicit, auditable, and less vulnerable to positive-outcome selection bias. |
| Data extracted | Source, engineering strategy, model, route, target compartment, direction of effect (benefit, null, or harm), functional/mechanistic readouts, human/clinical evidence, and major limitation. | Aligns narrative text and evidence-map fields and preserves negative or counter-evidence. |
| Interpretive boundary | No meta-analysis or formal risk-of-bias tool; mechanistic descriptors are not study-quality scores; absence from the table is not evidence of ineffectiveness, and a negative result from one preparation does not invalidate an entire platform class. | Prevents overinterpretation of either positive or negative findings in a narrative evidence map. |
| Evidence-descriptor assignment | Model relevance, mechanistic causality, delivery evidence, translational readiness, and clinical status were assigned using prespecified operational definitions aligned with Table 1. When evidence was ambiguous, the more conservative descriptor was retained. | Improves consistency and reduces overinterpretation across heterogeneous vesicle platforms. |
| Evidence assessment process | Two authors independently assessed the evidence descriptors after study-level data extraction. Disagreements were resolved through discussion and consensus. | Reduces individual interpretive bias and improves assessment consistency. |

Evidence-descriptor definitions. Descriptive association indicates that treatment effects and biological changes occurred concurrently without direct perturbation testing. A defined mechanistic link requires evidence connecting a specific cargo, surface signal, formulation, or pathway with a functional outcome. Dependency terminology is reserved for blockade, loss-of-function, rescue, transfer, receptor-dependence, or equivalent perturbation experiments. Delivery evidence distinguishes tissue exposure, target-cell engagement, functional cargo delivery or receptor-axis signaling, and validated targeting. Validated targeting additionally requires preferential localization and disease-relevant cellular engagement, ideally supported by ligand- or receptor-dependence. Translational readiness reflects product identity, dose definition, potency testing, safety assessment, manufacturing control, and human-relevant validation rather than therapeutic efficacy alone. Negative/counter-evidence denotes direct evidence of product instability, absent therapeutic benefit, or harmful biological activity; such findings are interpreted as preparation-specific unless broader class-level evidence is available.

# Table S4. Updated-search audit and study-selection disposition

**Scope note.** This table records studies already represented in the main evidence map, reviewer-specified additions, all PubMed foundational-module records published from 16 June through 5 August 2026, and the three additional eligible original studies found by OpenAlex after deduplication. Main-text selection is distinguished from supplementary retention, context-only use, and exclusion.

| **Record** | **Study/platform** | **Disposition** | **Reason** |
| --- | --- | --- | --- |
| Ref 24 | BM-MSC EVs - Cao et al. | Included in main evidence map | Foundational mammalian EV study; acute DSS model; macrophage/cytokine outcomes. |
| Ref 25 | BMSC-derived exosomes carrying TSG-6 - Wu et al. | Included in main-text narrative and supplementary evidence ledger | Original acute DSS-colitis study with epithelial-pyroptosis and barrier readouts plus donor-cell TSG-6 perturbation in complementary cell experiments; retained as a distinct preparation-specific mammalian EV study. |
| Ref 26 | hUC-MSC EVs carrying miR-326 | Included in main evidence map | Defined cargo/pathway link; preparation-specific preclinical evidence. |
| Ref 27 | hUC-MSC EVs carrying miR-129-5p | Included in main evidence map | Defined cargo/ACSL4-ferroptosis link. |
| Ref 34 | Naive and cytokine-primed MSC-EVs versus parental MSCs - Tolomeo et al. | Included as counter-evidence and comparative evidence | Original DSS-colitis comparison showing divergent effects of MSCs and their EVs and conditioning-dependent EV activity; prevents inference of EV behavior from source-cell effects. |
| Ref 35 | Unloaded bovine milk EVs - Seegobin et al. | Included as negative evidence | Biorelevant gastrointestinal stability failure and null DSS efficacy. |
| Ref 67 | Milk EVs carrying anti-TNF-alpha nanobody/LL37 | Included in main evidence map | Engineered oral milk-EV platform; cargo and barrier readouts. |
| Ref 7 | Grape EV-like nanoparticles | Included in main evidence map | Stem-cell engagement and lipid/beta-catenin perturbation. |
| Ref 8 | Broccoli-derived nanoparticles | Included in main evidence map | AMPK dependence and dendritic-cell transfer evidence. |
| Ref 9 | Ginger-derived nanoparticles | Included in main evidence map | Representative natural edible particle platform with multiple intestinal readouts. |
| Ref 10 | Turmeric-derived nanovesicles - Gao et al. | Included; separate from Ref 43 | Distinct preparation and findings; must not be grouped with another turmeric preparation. |
| Ref 44 | Turmeric-derived exosome-like nanovesicles - Liu et al. | Included; separate from Ref 10 | Distinct preparation and findings; retained separately for preparation-specific interpretation. |
| Ref 54 | Momordica charantia-derived EVs | Included in main evidence map | Recent plant platform with barrier, microbiota, and oxidative-stress readouts. |
| Ref 11 | HA-modified red cabbage particles | Included in main evidence map | Engineering/targeting comparison with unmodified preparation. |
| Ref 6 | PD-L1/miR-27a-3p EVs | Included in main evidence map | Relatively strong receptor/pathway perturbation and patient-derived systems. |
| Ref 71 | Biomaterial-retained MSC EVs - Gan et al. | Included in main evidence map | Formulation-enabled oral retention platform. |
| Ref 64 | Gal-IL10-EVs | Included in main evidence map | Donor engineering, protected oral delivery, and partial mechanism support. |
| Ref 72 | MSC-IL-27 EV microcarriers - Nie et al. | Included in main evidence map | Local retention and cytokine-engineered EV platform. |
| Ref 68 | Milk EVs carrying TNF-alpha siRNA | Included in main evidence map | Defined cargo/target suppression and oral delivery. |
| Ref 69 | Protein-hybrid EVs - Lee et al. | Included in main evidence map | Acid-resistant hybrid protein cargo platform. |
| Ref 65 | PrEXO-a23 hybrid vesicles | Included in main evidence map | Multi-model engineered platform including fibrosis and CAC. |
| Ref 33 | MSC exosomes for perianal CD fistula | Included clinical evidence | Five-patient uncontrolled phase I local-injection study. |
| PMID 41983055 | Pueraria lobata decoction-derived vesicle-like nanoparticles | Included in revised evidence map | Chronic DSS model with pseudo-germ-free loss-of-efficacy evidence supporting microbiota dependence. |
| PMID 41639673 | Flos Sophorae immaturus exosome-like nanovesicles | Included in revised evidence map | Antibiotic, FMT, organoid, mono-colonization, and AhR-antagonist evidence supports a microbiota-tryptophan-AhR branch. |
| DOI 10.24976/Discov.Med.202537199.144 | Bafilomycin A1-conditioned iPSC-MSC EV production | Included as manufacturing/translational evidence | Donor-cell conditioning increases EV yield; efficacy reported as comparable to untreated EVs in an IBD model. |
| PMID 42548959 | Thermally reassembled ginger EVs | Included in revised evidence map | Original therapeutic platform; heat processing, intestinal uptake, and NLRP3-related activity. |
| PMID 42544276 | Magnolia biondii decoction-derived exosome-like nanoparticles | Included in revised evidence map | Original decoction-derived therapeutic study; relevant to processing and co-isolate interpretation. |
| PMID 42499024 | Nanotechnology in IBD bibliometric analysis | Excluded | Bibliometric study; no vesicle intervention. |
| PMID 42468020 | Probiotic-derived EVs in a thermogelling rectal copolymer | Included in revised evidence map | Original probiotic-vesicle/biomaterial intervention; addresses Reviewer 2's bacterial-vesicle scope request. |
| PMID 42463074 | MSC and MSC-exosome gut-microbiota review | Context only | Review article; not an original therapeutic study. |
| PMID 42447860 | Candida EV antigens and pathogenic Th17 cells | Context-only counter-evidence | Disease-mechanism study without therapeutic intervention; relevant to pathogenic microbial EV risk. |
| PMID 42440456 | Meta-analysis of plant-derived particles in UC | Context only | Secondary synthesis; not an original therapeutic study. |
| PMID 42439691 | IL-33-stimulated macrophage exosomes | Included in revised evidence map | Original TNBS-colitis therapeutic study; donor conditioning and epithelial-barrier mechanism. |
| PMID 42432695 | Extremophyte-derived exosome-like nanovesicles | Included in revised evidence map | Reviewer-specified recent original study; barrier, immune, and microbiota outcomes. |
| PMID 42406421 | Thymus-derived IL-7R-enriched pathogenic EVs | Context-only counter-evidence | Pathogenic EV mechanism; retained to illustrate source/cargo-dependent harm. |
| PMID 42390648 | BMSC exosomes carrying miR-148a-3p | Included in supplementary evidence ledger | Original therapeutic cargo/pyroptosis study; near-duplicate mammalian platform not prioritized for expanded main-text discussion. |
| PMID 42378030 | Donkey milk exosomes | Included in supplementary evidence ledger | Original milk-EV therapeutic study with miRNA and microbiota claims. |
| PMID 42376871 | Double-coated oral MSC-EVs | Included in revised evidence map | Original oral-delivery study addressing gastrointestinal stability and colon targeting. |
| PMID 42364261 | Microbiome-epigenome axis review | Excluded | Broad narrative review; not specific original EV therapy evidence. |
| PMID 42333832 | Postbiotic extracellular vesicles for UC | Context only | Review/perspective; useful for scope but not original efficacy evidence. |
| PMID 42303094 | Edible extracellular vesicles for IBD review | Context only | Review article; not an original therapeutic study. |
| PMID 42295683 | Cucurbitacin derivatives review | Excluded | Non-vesicular intervention. |
| PMID 42250389 | Polyphenols as prebiotics review | Excluded | Non-vesicular intervention. |
| PMID 42213778 | Milk EVs in pre-IBD | Supplementary/context only | Original intervention but uses a pre-IBD state rather than established IBD/colitis; not used for therapeutic efficacy conclusions. |
| PMID 42160026 | Food-derived biohybrid probiotic EVs | Included in revised evidence map | Original engineered probiotic-vesicle therapy with gastrointestinal protection and multi-omics readouts. |
| PMID 42107777 | Hybrid EV therapy targeting the IgA/pIgR axis | Included in revised evidence map | Original oral hybrid-EV study with a defined mucosal transport target. |
| PMID 42068599 | Bacterial EVs in the oral-gut axis | Context only | Review; supports balanced discussion of commensal and pathogenic BEVs. |
| PMID 42034038 | Parabacteroides distasonis-derived EVs | Included in revised evidence map | Original bacterial EV therapy with microbiota/tryptophan/AhR mechanism. |
| PMID 42030800 | Chlorella pyrenoidosa-derived EVs | Included in supplementary evidence ledger | Original microalgal EV intervention; expands source taxonomy. |
| PMID 42019096 | Fibroblasts in IBD-associated CRC review | Excluded | No vesicle intervention. |
| PMID 41926795 | hUCMSC-sEV miR-320d study | Included in supplementary evidence ledger | Original cargo/ferroptosis study; near-duplicate mammalian platform retained in supplement. |
| PMID 41875611 | Perilla frutescens-derived nanovesicles | Included in supplementary evidence ledger | Original oral plant-vesicle therapy; main-text expansion optional due platform redundancy. |
| PMID 41865696 | Callicarpa nudiflora EV-like particles carrying budesonide | Included in revised evidence map | Original cargo-loaded plant-particle study with free-drug comparator. |
| PMID 41865564 | Dual-coated macrophage EVs carrying 5-ASA | Included in revised evidence map | Original oral engineered-EV study with stability and delivery controls. |
| DOI 10.3389/fmicb.2026.1876636 | EVs from pasteurized Akkermansia muciniphila | Included in supplementary evidence ledger | OpenAlex-identified original DSS-colitis study without a PMID on the search date; compares vesicles from live and pasteurized bacteria. |
| DOI 10.1186/s12951-026-04866-5 | Bletilla striata EV-armed Escherichia coli Nissle 1917 | Included in revised evidence map | OpenAlex-identified original hybrid plant-EV/probiotic study published on 1 August 2026; adds a distinct cross-species delivery platform. |
| DOI 10.1016/j.apsb.2026.07.044 | Portulaca oleracea EV-like particles carrying miR159-y | Included in revised evidence map | OpenAlex-identified original IBD/CAC study without a PMID on the search date; reports biodistribution and a defined miRNA/TGFBR2/ILC mechanism. |
| Ref 30; PMID 39583323; DOI 10.2147/IJN.S494321 | LPS-pretreated PDLSC EVs - Tang et al. | Included in revised evidence map | Original acute DSS-colitis study comparing conditioned and unconditioned PDLSC EVs; conditioning benefit was observed, whereas PI3K/AKT dependency and the active cargo were not tested. |
| Ref 63; PMID 41993781; DOI 10.2147/IJN.S584494 | MSC-EVs in IBD and bioengineering - Li et al. | Context only | Recent review used to frame source selection, targeting, cargo engineering, and biomaterial integration; not entered in the original-study evidence ledger. |
| Ref 95; PMID 37256205; DOI 10.2147/IJN.S402179 | hUC-MSC exosomes - Liang et al. | Included in revised evidence map | Original DSS/TNBS and organoid study with Lgr5 and epithelial-repair readouts plus Wnt-C59 pathway inhibition; no lineage tracing or long-term functional validation. |
| Ref 96; PMID 41773922; DOI 10.1093/ibd/izag014 | iPSC-MSC-derived exosomes - Feng et al. | Included in revised evidence map | Original TNBS study with Lgr5/Bmi1/Ki-67 readouts, miR-34a-5p gain/loss experiments, PPP2R3A/Wnt linkage, and patient CD LPMC assays; no lineage tracing or durable functional validation. |

# Table S5. Study-level therapeutic evidence ledger

Evidence-availability note. Full texts and available supplementary files for all included reports were reviewed. 'Not reported' or 'unclear' indicates that the source did not provide the relevant information or contained an internal inconsistency.

**Table S5A. Product identity, preparation, model, dose, and route**

| **Study / main-text linkage** | **Product/source** | **Preparation & characterization** | **Model/phenotype** | **Dose/schedule/timing** | **Route** |
| --- | --- | --- | --- | --- | --- |
| BM-MSC EVs - Cao et al. (24) | Native/unmodified EVs from mouse bone-marrow MSCs | Serum-free low-glucose DMEM for 48 h; conditioned medium centrifuged at 1,000 x g, 2,000 x g, and 10,000 x g (20 min each), then 100,000 x g for 70 min; pellet stored at -80 C. TEM and western blot for CD63, CD81, and TSG101; particle concentration and a negative marker were not reported. | Male BALB/c mice (7-8 weeks); 3% DSS for 7 days; n=10/group | 50 microg EVs/mouse/day for 7 days during DSS exposure; sacrifice on day 7 | Intraperitoneal |
| BMSC-derived exosomes carrying TSG-6 - Wu et al. (25) | Native exosomes from passage 3-5 C57BL/6J mouse bone-marrow MSCs; siTSG-6 and control-siRNA donor preparations were used for complementary in-vitro cargo perturbation. | Exosome-depleted-serum medium for 48 h; sequential 300 x g (10 min), 2,000 x g (10 min), and 10,000 x g (30 min) clarification at 4 C; 100,000 x g for 70 min, PBS wash, and repeat ultracentrifugation. TEM and immunoblotting for CD63, CD81, and TSG101; protein quantified by BCA. | Male C57BL/6J mice (8 weeks); 2.5% DSS for 7 days followed by water; acute colitis; n=5/group. Complementary LPS-stimulated Caco-2 epithelial-cell model. | Reported as a 1-mg exosomal-protein dose administered on days 4, 6, and 8; the article does not clearly state whether 1 mg denotes each injection or the cumulative schedule. Mice were euthanized on day 10. Caco-2 cells received 100, 200, or 400 microg/mL exosomes. | Intraperitoneal in mice; direct addition in vitro |
| hUC-MSC EVs - miR-326 EVs (26) | miR-326 cargo | hUC-MSC conditioned medium was cleared of cells/debris, concentrated with a 100-kDa ultrafiltration device, precipitated overnight with an SBI reagent, washed, resuspended in PBS, passed through a 0.22-micro m filter, and stored at -80 C. NTA, TEM, and western blotting for CD9/CD63/CD81 were reported. | Male BALB/c mice (6 weeks); 3% DSS acute colitis; n=6/group; sacrifice on day 10 | 1 mg hUC-MSC exosomes/mouse by tail vein on days 3, 6, and 9. The same dose/schedule was used for the modified-cargo comparisons but was not separately restated for every arm. | Tail-vein injection |
| hUC-MSC EVs - miR-129-5p EVs (27) | miR-129-5p cargo | hUC-MSC conditioned medium underwent serial clarification, 100-kDa ultrafiltration, ultracentrifugation at 100,000 x g, PBS resuspension, 0.22-micro m filtration, and -80 C storage. NTA, TEM, and western blotting for CD9/CD81/ALIX with calnexin as a negative marker were reported. | Mouse 3% DSS acute colitis; outcomes assessed on day 10 | 1 mg exosome protein per 20 g body weight (50 mg/kg) by tail vein on days 3, 6, and 9. Modified miR-129-5p groups followed the study protocol, although the dose was not restated in every experiment. | Tail-vein injection |
| Naive and cytokine-primed MSC-EVs versus parental MSCs - Tolomeo et al. (34) | Small EV-enriched fractions from passage-5 murine bone-marrow MSCs cultured either without priming or for 24 h with IL-6 (20 ng/mL), TNF-alpha (25 ng/mL), and IL-1beta (25 ng/mL), followed by 24-h collection medium. | Conditioned medium centrifuged at 1,200 rpm for 6 min, filtered through 0.22 micro m, and concentrated by 100,000-NMWL Amicon ultrafiltration at 3,200 x g, 4 C, for 15 min. TRPS/qNano particle concentration and size; TEM; CD9/CD63 plus ALIX and TSG101 characterization. | C57BL/6N mice (8-12 weeks); 3% DSS for 6 days followed by 3 days of water; n=5/group; euthanasia on day 10. | 1.00 x 10^9 naive EVs or cytokine-primed EVs per administration on days 4, 6, and 8 in 200 microL PBS. Comparator MSC groups received 4.00 x 10^6 cells per administration on days 4 and 8. | Intraperitoneal |
| Unloaded bovine milk EVs - Seegobin et al. (35) | Native/unloaded small EVs from pasteurized, unhomogenized semi-skimmed bovine milk | Milk diluted in PBS; 3,000 x g for 15 min; equal-volume 0.25 M EDTA on ice for 15 min; sequential 12,000, 35,000, and 70,000 x g spins (1 h each) with 0.22-micro m filtration after each; 100,000 x g for 2 h; qEV Gen2 SEC. NTA, zeta potential, BCA, TEM, TSG101/CD81 with calnexin-negative western blot, pH testing, laurdan bilayer assay, and model-cargo release studies. | Female C57BL/6 mice (6 weeks); 2% DSS on days 1-5 of an 8-day study; n=7/group | 1 mg EVs/mouse/day for 8 consecutive days; oral volume 100 microL or rectal volume 50 microL | Oral gavage or rectal enema |
| Milk EVs - anti-TNF-alpha nanobody / LL37 (67) | Nanobody and LL37 loading | Commercial pasteurized milk was clarified at 13,000 x g and filtered (0.22 micro m). Casein was dissociated with 0.25 M EDTA, removed after dialysis/Ca2+-driven reaggregation, and EVs were pelleted at 150,000 x g for 1.5 h. NTA, TEM, and CD63/CD81/Alix western blots were reported; calnexin and casein were assessed as contaminants. TAT-fused VHH or LL37 was incubated with EVs (3 microg cargo/10 microg EV) and purified by ultrafiltration. | Male C57BL/6 mice; 3% DSS acute colitis and a three-cycle DSS chronic-colitis model | Acute model: oral treatment daily for 7 days from day 8; EVs 50 mg/kg, free VHH or LL37 0.8 micromol/kg, or loaded EVs containing 50 mg/kg EV plus 0.8 micromol/kg cargo. Chronic model used two 14-day treatment periods beginning on days 8 and 29; the wording of the schedule is not fully explicit. | Oral gavage |
| Grape EV-like nanoparticles (7) | Natural lipid-rich particles | Washed, peeled grapes were pressed; juice was diluted in cold PBS and purified by differential centrifugation followed by sucrose-gradient ultracentrifugation (individual spin settings were cited to an earlier protocol rather than restated). Cryo-EM, size/zeta-potential, lipidomic, protein, and RNA analyses were reported; pellets were weighed for dosing. | Male Lgr5-EGFP-IRES-CreERT2 mice (6 weeks, 18-25 g); 3% DSS colitis; intestinal crypt/organoid and reporter experiments | 2 mg GELNs/mouse/day by oral gavage during DSS exposure. Ancillary trafficking experiments used 1 mg/mouse in 200 microL PBS; homeostatic Wnt/stem-cell experiments used 2 mg/mouse/day for 7 days. | Oral gavage |
| Broccoli-derived nanoparticles (8) | Plant lipids and sulforaphane-related activity | Broccoli was homogenized and sequentially centrifuged (1,000 x g for 10 min, 3,000 x g for 20 min, 10,000 x g for 40 min, then 100,000 x g for 90 min); the pellet was further purified by Biomax-500 filtration and sucrose fractionation. Electron microscopy, size distribution, zeta potential, and protein quantification were reported. | C57BL/6 DSS colitis, naive-CD4 T-cell-transfer colitis, and anti-CD40 Rag1-knockout colitis | 250 microg BDNs/mouse in 200 microL PBS orally daily for 10 days before 2.5% DSS and every 2 days for 12 days thereafter; twice weekly after T-cell transfer; or daily for 10 days before anti-CD40 challenge. | Oral gavage |
| Ginger-derived nanoparticles (9) | Lipids, miRNAs, and ginger compounds | Ginger juice was clarified at 3,000 x g for 20 min and 10,000 x g for 40 min, pelleted at 150,000 x g for 2 h, and fractionated on an 8%/30%/45%/60% sucrose gradient at 150,000 x g for 2 h; the 30%-45% GDNP2 band was used. DLS/zeta potential, AFM/TEM, and proteomic, lipidomic, and miRNA analyses were reported. | FVB/NJ 1.5% DSS acute colitis and wound-healing models; IL-10-knockout spontaneous colitis; AOM/DSS colitis-associated cancer | 0.3 mg GDNP2/mouse orally each day for 7 days in acute DSS; daily during the DSS/recovery wound-healing experiment; daily from 4 weeks of age in IL-10-knockout mice; and daily throughout the AOM/DSS CAC protocol. | Oral gavage |
| Turmeric-derived nanovesicles - Gao et al. (Ref 10) | Curcumin-associated cargo | Fresh turmeric juice was sequentially centrifuged at 1,000, 2,000, 3,000, and 10,000 x g for 10, 20, 30, and 60 min; vesicles were pelleted at 150,000 x g for 1.5 h and purified on an 8%/30%/45%/60% sucrose gradient at 150,000 x g for 1.5 h. DLS, TEM, proteomics, lipidomics, and LC-MS curcuminoid measurements were reported. | ICR mice (6-8 weeks); 3.5% DSS acute colitis and three-cycle 3% DSS chronic colitis | Acute model: TNVs 10 mg/kg/day orally (5-ASA 100 mg/kg positive control) during a 12-day study. Chronic model: TNVs or 5-ASA orally every other day during each DSS-free interval; the chronic-study dose was not restated in the methods. | Oral administration |
| Turmeric-derived exosome-like nanovesicles - Liu et al. (Ref 44) | Curcumin-associated cargo | Turmeric was homogenized and processed by differential centrifugation and sucrose-gradient ultracentrifugation to obtain TDNP1 (8%-30% interface) and TDNP2 (30%-45% interface); exact differential-spin settings were not restated in this report. DLS, TEM/AFM, zeta potential, HPLC curcumin measurement, proteomics, and lipidomics were reported. | FVB/NJ mice; 3% DSS acute colitis and a DSS-withdrawal wound-healing model | 3 mg TDNP1 or TDNP2/mouse/day orally for 7 days during 3% DSS; in the wound-healing experiment, 3 mg TDNP2/mouse/day for 1 week after 7 days of DSS. | Oral gavage |
| Momordica charantia-derived EVs - Gao et al. (54) | Lipid- and carotenoid-associated cargo | Pith-free Momordica charantia juice was centrifuged at 3,000 x g for 30 min and 10,000 x g for 40 min, pelleted at 150,000 x g for 90 min, purified at the 30%-45% interface of an 8%/30%/45% sucrose gradient (150,000 x g, 2 h), and washed at 150,000 x g for 2 h. TEM, DLS/zeta potential, and compositional analyses were reported. | Mouse DSS-induced acute ulcerative-colitis model; n=6/group. The DSS concentration was not explicitly stated in the published methods. | Therapeutic experiment: 15 or 30 mg/kg orally once daily for 5 days beginning on day 6; harvest on day 11. A concurrent/protective experiment used the same doses on alternate days during an 8-day DSS protocol. Safety study: 30 mg/kg/day for 7 days. | Oral gavage |
| Engineered PELNs - HA-modified red cabbage particles (11) | HA surface conjugation | Red-cabbage Rabex particles were obtained by blending, centrifugation, ultrafiltration, and size-exclusion chromatography (fractions 7-9). Hyaluronic acid was displayed by insertion of DSPE-PEG-HA to form t-Rabex. NTA, TEM, zeta potential/PDI, and stability tests were reported. | Mouse 3% DSS for 5 days followed by water; 14-day experimental-colitis protocol | Ten oral doses over 12 days (one before DSS, five during DSS, four after DSS): Rabex low dose 5 × 10^10 particles/mL, Rabex high dose 5 × 10^11 particles/mL, or t-Rabex low dose 5 × 10^10 particles/mL. Supplementary Figure S1 defines 5 × 10^10 particles as one dose for its cross-source cost comparison; however, the mouse gavage volume and administered particle number per mouse are not explicitly stated in the in-vivo protocol. Sulfasalazine control: 100 mg/kg/day. | Oral gavage |
| Checkpoint EVs - PD-L1 / miR-27a-3p EVs (6) | PD-L1 surface signal and miR-27a-3p cargo | Wharton's-jelly MSCs were dual lentivirally engineered to express PD-L1 and miR-27a-3p, selected by puromycin/FACS, and cultured under the reported priming conditions. The publisher supplementary methods report collection from EV-depleted conditioned medium, serial centrifugation at 300 × g, 2,500 × g, and 10,000 × g, followed by ultracentrifugation at 100,000 × g; the EV pellet was washed, passed through a 0.2-µm filter, and resuspended in PBS. NTA, TEM, zeta potential, and western blotting for CD63/CD81 with calnexin as a negative marker were reported. Individual spin durations were not stated. | Male C57BL/6 3% DSS colitis; PBMC-humanized NSG TNBS colitis; patient PBMC and colonoid experiments | DSS: total 300 microg EVs/mouse intraperitoneally on days 1, 3, and 5; sacrifice day 10. Humanized TNBS: total 400 microg EVs/mouse intravenously in preventive (days 6-9) and therapeutic (days 13-17) schedules. Short-term safety: 500 microg/mouse once intravenously. | Intraperitoneal or intravenous, model-dependent |
| Biomaterial-retained MSC EVs - Gan et al. (71) | hUC-MSC exosomes encapsulated in sodium-alginate/gelatin microcapsules with an outer Eudragit FS30D enteric coating (EGAE) | Serum-free hUC-MSC supernatant; 10,000 x g for 30 min and 100,000 x g for 60 min, repeated three times; PBS resuspension and 0.22-micro m filtration. BCA, TEM, particle sizing, and western blot markers. Exosomes (20 mg/mL) were loaded into 1% alginate and CaCl2-solidified, enclosed in 60% gelatin by W/O microfluidics, and Eudragit-coated; microscopy/SEM, encapsulation, storage, simulated GI degradation, release, and biodistribution were assessed. | Male C57 mice; continuous 5% DSS acute colitis; 30 mice randomized to five groups (n=6/group) | 20 mg exosome protein/kg by oral gavage every other day beginning on day 2 of DSS exposure; outcomes assessed on day 8 | Oral gavage |
| Gal-IL10-EVs (C/A) - Liu et al. (64) | HEK293T EVs donor-engineered to contain murine IL-10, surface-modified with DSPE-PEG-galactose, and protected by an in-situ chitosan/alginate hydrogel | Conditioned medium centrifuged at 300 x g for 15 min, 2,000 x g for 20 min, and 4,696 x g for 25 min at 4 C; 0.45-micro m filtration; 120,000 x g for 70 min, wash, and PBS resuspension. NTA, TEM, TSG101/CD63/CD81/IL-10 western blot, IL-10 ELISA, and BCA. EVs (1 mg/mL) were mixed with DSPE-PEG-galactose (0.1 mg/mL) for 30 min and re-pelleted; 3:7 chitosan/alginate hydrogel was formed by sequential double gavage. | Male C57BL/6 mice (7-8 weeks); 4% DSS for 7 days; 20 mice randomized to five groups (n=4/group) | 0.3 mg EV protein/kg by oral gavage on days 3 and 5; separate biodistribution used a single 0.6 mg/kg dose with 12-, 24-, and 48-h sampling | Oral double gavage |
| MSC-IL-27 EV microcarriers - Nie et al. (72) | IL-27-high MSC-EVs | IL-27-overexpressing MSC conditioned medium was clarified at 300 x g for 10 min, 2,000 x g for 10 min, and 10,000 x g for 30 min, then ultracentrifuged twice at 100,000 x g for 70 min at 4 C. TEM, DLS, CD81 western blotting, and IL-27 measurement were reported. GelMA/dopamine-methacrylamide pregel was mixed with 10 mg EVs/mL and formed into UV-solidified microfluidic particles. | Rat 5% DSS colitis for 10 days; in-vitro LPS-stimulated epithelial/macrophage assays | Rectal treatment on days 1 and 3; 500 microL of the corresponding microparticle suspension was used in the adhesion experiment. The formulation contained 10 mg EVs/mL pregel, but the therapeutic per-rat EV amount was not explicitly stated; sacrifice on day 7. | Rectal enema |
| Milk EVs carrying TNF-alpha siRNA - Han et al. (68) | Electroporation-loaded siRNA | Low-fat milk was clarified at 5,000 x g for 30 min and 12,000 x g for 1 h, fractionated by sucrose-gradient ultracentrifugation for 3 h, and washed at 100,000 x g for 1 h. TNF-alpha siRNA (125 pmol/10^10 particles) was loaded by five 300-V electroporation pulses, followed by 3 h recovery and 30-kDa ultrafiltration. NTA, cryo-TEM, TSG101/CD9 western blots, negative markers, and lipidomics were reported. | Female BALB/c mice; 2.5% DSS and delayed-treatment 3% DSS colitis models | Intraperitoneal every 3 days for four doses from day 16: 16.1 nmol siRNA/kg plus 2 x 10^12 particles/kg, or 32.1 nmol/kg plus 4 x 10^12 particles/kg. Oral schedule: 6.42 nmol siRNA/kg plus 8 x 10^11 particles/kg every 3 days; delayed therapy used the oral dose three times at 3-day intervals. | Oral gavage or intraperitoneal injection |
| Protein-hybrid EVs - Lee et al. (69) | Backbone-cyclized immunoregulatory protein | Paeonia lactiflora-root vesicles were prepared by screw extraction, mesh clarification, centrifugation at 2,000 x g for 30 min and 12,000 x g for 60 min, and 100-kDa tangential-flow filtration. NTA, DLS, and TEM were reported. A backbone-cyclized C-R4-ppTCPTP protein was passively incubated with PeoEVs and purified with ExoQuick. | Male C57BL/6 mice (7 weeks); acute DSS colitis; intestinal organoid and macrophage-conditioned-medium assays | Daily oral treatment for 8 days: PeoEVs 1 x 10^9 particles/mouse or Cyc@PeoEV containing a total cyclic-protein concentration corresponding to 50 micromol/L; 5-ASA 50 mg/kg comparator. Methods state 2.5% DSS, whereas the figure text states 3.0% DSS. | Oral gavage |
| PrEXO-a23 hybrid vesicles - Cao et al. (65) | Treg-exosome/platelet-membrane hybrid | Treg-derived rEXOs were isolated by ultracentrifugation and fused 1:1 by mass with platelet-membrane vesicles by extrusion. Anti-IL-23 was attached through an MMP-cleavable linker (optimized PrEXO:a23 mass ratio 1:0.5). TEM, NTA/zeta potential, immunogold imaging, and western blots for exosome, platelet, CCR6, and antibody components were reported. | Female C57BL/6 3% DSS acute colitis and AOM/2.5% DSS colitis-associated-cancer model | Acute DSS: 5 mg/kg rEXO-equivalent plus 1.5 mg/kg anti-IL-23 intravenously on days 2, 4, and 6; sacrifice day 8. AOM/DSS model: intravenous treatment every 2 days during each DSS period. | Intravenous injection |
| MSC exosomes - perianal CD fistula phase I (33) | Native MSC exosomes | Third-passage hUC-MSCs were cultured for 48 h in exosome-depleted medium; conditioned medium was clarified, centrifuged at 2,500 x g for 30 min, ultracentrifuged at 100,000 x g for 120 min, washed, and re-spun at 100,000 x g for 120 min. DLS, TEM, and CD9/CD63/CD81 assays were reported. | Five patients with refractory perianal Crohn's-disease fistulas; open-label phase-I study; follow-up to 6 months | Single perilesional injection of 5 mL exosome suspension at 50 microg/mL (250 microg total), placed 2-3 mm around the fistula tract. | Local perianal injection |
| Liu et al. 2026; PMID 41983055 [main-text Ref 56] | Pueraria lobata (GeGen) decoction-derived vesicle-like nanoparticles; heat/decoction-processed plant product | Rhizome powder boiled in water (1:6) for 45 min; 1,000×g 10 min, 3,000×g 20 min, 10,000×g 40 min, then 150,000×g 70 min (4 °C); PBS resuspension. TEM/NTA, particle count, zeta potential, simulated-GI stability and HPLC puerarin analysis. | Male C57BL/6J mice (6–8 weeks); acute 2% DSS colitis for 7 days; antibiotic-depleted pseudo-germ-free cohort | 15 mg/kg vesicle fraction by daily oral gavage for 7 days during DSS; separate whole-decoction and vesicle-depleted decoction arms at 37 mg/kg; reported puerarin-equivalent exposure 0.531 mg/kg | Oral gavage |
| Wu et al. 2026; PMID 41639673 [main-text Ref 57] | Flos Sophorae immaturus exosome-like nanovesicles | Fresh juice sequentially cleared at 500×g 10 min, 2,000×g 20 min and 10,000×g 30 min; 0.45-µm filtration; 120,000×g 60 min (4 °C), followed by sucrose-density purification. TEM/size/purity and biosafety characterized. | Male C57BL/6J mice (6–8 weeks); acute 3% DSS colitis for 7 days; organoids, antibiotic/FMT and mono-colonization cohorts | 5 or 10 mg/kg daily for 7 days during DSS (dose-ranging included 1–20 mg/kg); 5-ASA 100 mg/kg positive control; additional IAA 20 mg/kg and AhR-antagonist experiments | Oral gavage |
| Xiao et al. 2025; DOI 10.24976/Discov.Med.202537199.144 [main-text Ref 104] | Bafilomycin A1-conditioned iPSC-MSC EVs; donor-conditioned mammalian product | Conditioned medium cleared at 500×g 10 min, 2,000×g 15 min and 10,000×g 30 min (4 °C), then 0.22-µm filtered and ultracentrifuged; TEM/NTA and CD63, CD9, TSG101 and ALIX characterization. Donor cells received 1 nM Baf-A1 for 10 days. | Acute 2.5% DSS colitis for 7 days in mice; separate acute liver-injury model | PBS, native iPSC-MSC EV or Baf-A1-conditioned EV given intravenously every 2 days beginning day −1; n=5/group; administered EV amount not stated in the article or supplementary file | Intravenous |
| PMID 42548959 [main-text Ref 59] | Fresh versus thermally reassembled ginger EVs; heat-processed plant product, with optional TNF-α siRNA loading | Ginger juice filtered through 100-mesh sieve; aliquots kept at 4 °C or boiled at 100 °C for 60 min; differential centrifugation plus commercial exosome-isolation kit. TEM/cryo-TEM, NTA, zeta, proteomics and metal/protein-shell analyses. | BALB/c acute 4% DSS colitis; cellular uptake and biodistribution studies | 5×10^9 particles of fresh or thermally reassembled EVs; siRNA-loaded arms delivered 30 nmol/kg TNF-α siRNA; 6-day treatment, euthanasia day 14 | Oral |
| PMID 42544276 [main-text Ref 60] | Magnolia biondii decoction-derived exosome-like nanoparticles | Dried Xinyi decoction filtered, then 8,000×g 30 min, 16,000×g 30 min, 0.22-µm filtration and 100,000×g 90 min (4 °C); PBS resuspension. TEM, size/zeta, concentration, BCA and HPLC-MS metabolomics. | Male C57BL/6 mice; acute 2.5% DSS for 5 days with follow-up to day 14; n=6/group; LPS macrophage assays | 12.5 mg/kg by daily oral gavage for 14 days starting with DSS exposure; vehicle controls | Oral gavage |
| PMID 42468020 [main-text Ref 75] | VSL#3 probiotic-derived EVs plus 5-ASA in a PNHA thermogelling hyaluronic-acid copolymer | Probiotic EVs prepared by ultrafiltration and 2-h ultracentrifugation; formulated at 100 µg/mL in 2.5% PNHA with 5-ASA. TEM/DLS/AFM, proteomics, rheology, release and retention characterized. | Female Wistar rats (6 weeks); TNBS colitis (100 mg/kg in 40% ethanol, intrarectal) for 7 days; six groups, n=8 | Rectal PNHA, PNHA–5-ASA, PNHA–ProEV or PNHA–5-ASA–ProEV beginning day 8 after bowel cleansing; exact per-rat formulation dose is reported only in Supplementary Table S2, not in the supplied main report | Intrarectal |
| PMID 42439691 [main-text Ref 29] | Exosomes from IL-33-stimulated RAW264.7 macrophages; donor-conditioned mammalian EVs | PEG precipitation followed by ultracentrifugation; BCA, NTA, TEM and CD63/HSP70/TSG101-positive, calnexin-negative characterization. | Male BALB/c mice (5–6 weeks); TNBS colitis induced intrarectally (2.5 mg in 150 µL 50% ethanol); n=6/group | 200 µg IL-33-stimulated or control-macrophage exosomes intraperitoneally 24 h after TNBS; XAV939 2.5 mg/kg used in the pathway-perturbation arm | Intraperitoneal |
| PMID 42432695 [main-text Ref 58] | Extremophyte-derived exosome-like nanoparticles from Rosa roxburghii (R-ELNs) and Artemisia sphaerocephala (A-ELNs) | Plant material was rinsed and homogenized in ice-cold PBS (pH 7.2), held at 4 °C for 12 h, then cleared at 1,200×g for 30 min, 3,000×g for 60 min and 10,000×g for 60 min (4 °C). ELNs were collected at 100,000×g for 120 min, quantified by BCA and stored at −20 °C. TEM, DLS/zeta potential, NTA and simulated-GI stability were assessed. | Male C57BL/6J mice (20–22 g); acute 4% DSS colitis for 7 days; macrophage polarization and secondary macrophage-exosome assays | R-ELNs at 1.5 or 3 mg/kg by oral gavage beginning 2 weeks before DSS and continuing during DSS; R-ELN/A-ELN comparison at 3 mg/kg; 3 mg/kg for 21 days in the safety cohort | Oral gavage |
| PMID 42390648 [Supplementary evidence ledger; not cited in main text] | Mouse bone-marrow mesenchymal-stromal-cell exosomes enriched in miR-148a-3p | Serum-free BMSC conditioned medium collected after 48 h was cleared at 300×g for 10 min, 2,000×g for 10 min and 10,000×g for 30 min, filtered through 0.22 µm, and ultracentrifuged twice at 100,000×g for 70 min (4 °C). Exosomes were resuspended in PBS, stored at −80 °C, and characterized by TEM/NTA and CD9, CD63 and TSG101 positivity with calnexin negativity. | Male C57BL/6 mice (6–8 weeks); acute 3% DSS colitis for 7 days; three groups, n=6; LPS/ATP-stimulated macrophage assays | 100 µg BMSC exosomes intraperitoneally on DSS days 2, 4 and 6; animals euthanized day 8; cellular assays used 100 µg/mL exosomes | Intraperitoneal |
| PMID 42378030 [Supplementary evidence ledger; not cited in main text] | Donkey-milk exosomes containing anti-inflammatory miRNAs | Milk defatted at 3,000×g 15 min; pH 4.6 casein removal; 5,000×g 30 min and 10,000×g 60 min; 120,000×g 90 min twice; PBS wash and 0.22-µm filtration. NTA/TEM, zeta, TSG101/CD63/CD9, lipidomics and miRNA profiling. | Male C57BL/6J mice (19±2 g); 3% DSS on days 15–21 after a 14-day prophylactic phase; four groups, n=8 | 0.8 or 3.2 µg/g (0.8 or 3.2 mg/kg) daily oral gavage for 14 days before DSS; saline controls; euthanasia day 22 | Oral gavage |
| PMID 42376871 [main-text Ref 76] | Chitosan/Eudragit S100 double-coated human bone-marrow MSC-EVs | MSC conditioned medium cleared at 500×g twice, 2,000×g 15 min and 0.22-µm filtration; 25% sucrose-cushion ultracentrifugation at 100,000×g 1.5 h plus PBS wash/re-spin. Sequential 0.1% chitosan and 0.2% Eudragit S100 coating; NTA, cryo-EM/SEM, markers and GI-fluid stability. | Male C57BL/6J mice (8–10 weeks); acute 3% DSS for 5 days; six groups, n=5 | 1×10^9 particles/mouse/day on DSS days 1–5; coated or uncoated EVs orally (100 µL/dose), with an IV EV arm and coated-empty-liposome control | Oral gavage; intravenous comparator |
| PMID 42160026 [main-text Ref 77] | Lactiplantibacillus plantarum EVs with chitosan/tannic-acid biohybrid coating (CS@EVs@TA) | Culture cleared at 8,000×g 15 min; 0.22-µm filtration, 100-kDa ultrafiltration at 4,500×g 20 min, 150,000×g 1.5 h and PBS re-spin; CS and TA self-assembled electrostatically. TEM/NTA, compositional, coating-efficiency and GI-stability analyses. | Male BALB/c mice (6–8 weeks); acute 3% DSS for 7 days followed by water; four groups | Uncoated EVs or CS@EVs@TA at 1 mg/kg (uncoated EV arm ≈1.63×10^9 particles), daily oral administration for 7 days; euthanasia day 15 | Oral |
| PMID 42107777 [main-text Ref 78] | Garlic-derived EVs fused with Bacteroides thetaiotaomicron EVs (GB EVs) to target the epithelial IgA/pIgR axis | Garlic EVs purified by differential centrifugation, 150,000×g ultracentrifugation and 8%–60% sucrose gradient; bacterial EVs by 0.22-µm filtration and ultracentrifugation. Parent EVs mixed 1:1 by particle number, pulse-sonicated and incubated at 37 °C; NTA/TEM/protein and GI-stability characterization. | Male C57BL/6J mice; acute 2.5% DSS prevention model and 3% DSS therapeutic model; chronic DSS characterization also reported | 1×10^10 particles/mouse by daily oral gavage: prevention on DSS days 0–7; therapeutic dosing on days 6–10 after 3% DSS on days 1–5 | Oral gavage |
| PMID 42034038 [main-text Ref 79] | Parabacteroides distasonis-derived extracellular vesicles | Culture cleared at 5,000 rpm for 20 min twice, filtered at 0.4 and 0.22 µm, then ultracentrifuged at 150,000×g for 2 h (4 °C); PBS wash/re-spin; TEM/NTA. | Male C57BL/6J mice (25±2 g); acute 3% DSS; preventive dosing cohort, n=5/group | 50 µg EVs in 200 µL PBS daily for 17 days, with DSS on days 10–17; AhR-inhibitor arm included | Oral gavage |
| PMID 42030800 [Supplementary evidence ledger; not cited in main text] | Chlorella pyrenoidosa-derived extracellular vesicles | Log-phase culture cleared at 4,000×g 20 min, 10,000×g 30 min and 20,000×g 30 min; 8%/30%/45%/60% sucrose gradient at 150,000×g 60 min; 30%–45% interface collected and 0.22-µm filtered. TEM/DLS, electrophoresis, TLC, WB and LC-MS/MS. | Male C57BL/6 mice (6 weeks); acute 2.5% DSS for 10 days; n=6/group; antibiotic-depleted and Caco-2 ferroptosis cohorts | 2 µg/g/day (2 mg/kg/day) by gavage during DSS; dose-range 0.7–2.64 µg/g/day; mesalazine 0.31 g/kg/day positive control | Oral gavage |
| PMID 41926795 [Supplementary evidence ledger; not cited in main text] | Human umbilical-cord MSC small EVs enriched in miR-320d | Ultracentrifugation-based isolation as previously reported; TEM/NTA and calnexin, HSP70, TSG101, CD81 and CD9 characterization; HFL1-sEV comparator and miRNA sequencing/target assays. | Male BALB/c mice (6–8 weeks, 20±2 g); 3% DSS model; three groups, n=5; LPS macrophage models | 1 mg hUCMSC-sEV by tail-vein injection on days 3, 6 and 9 during DSS; euthanasia day 11 | Intravenous (tail vein) |
| PMID 41875611 [Supplementary evidence ledger; not cited in main text] | Perilla frutescens leaf-derived nanovesicles | Fresh leaves homogenized in PBS (1:2); 100-µm filtration; 1,000×g 10 min, 3,000×g 20 min, 10,000×g 30 min; 0.45/0.22-µm filtration; 100,000×g 70 min, PBS wash and repeat spin. TEM/NTA/zeta, RNA/protein/lipid/metabolite multi-omics, gastric stability and endotoxin testing. | Male C57BL/6 mice (8 weeks); acute 3.5% DSS for 7 days after 7-day prophylaxis; n=6/group; FMT cohort | 5 or 10 mg/kg by daily oral gavage for 7 days before DSS and continued for 7 days during DSS | Oral gavage |
| PMID 41865696 [main-text Ref 80] | Callicarpa nudiflora EV-like particles loaded with budesonide | Fresh-leaf juice cleared at 1,000×g 15 min, 5,000×g 30 min, 10,000×g 60 min and 150,000×g 2 h; 8%/30%/45%/60% sucrose gradient at 150,000×g 2 h; 30%–45% fraction washed/re-spun. Bud loaded by co-incubation; unencapsulated drug removed by 450-nm filtration. | BALB/c mice; acute 3.5% DSS for 7 days followed by 7-day treatment; n=4 for reported colon outcomes; LPS macrophage and Caco-2 assays | Oral CN-EVLP, free budesonide or CN-EVLP–Bud on days 8–14; formulations normalized to budesonide 1 mg/kg | Oral |
| PMID 41865564 [main-text Ref 74] | M2-macrophage extracellular vesicles in a dual-coated, oral 5-aminosalicylic-acid formulation (DCEV) | PMA-differentiated THP-1 macrophages were rested for 24 h and polarized with IL-4 (30 ng/mL) for 72 h. Conditioned medium was concentrated by tangential-flow filtration and EVs purified by size-exclusion chromatography. EVs were combined with DOTAP:DOPE liposomes (3:1; equal particle numbers, 37 °C for 1 h) and then coated with 0.6% sodium alginate containing 25 µM 5-ASA. NTA, SDS-PAGE, CD63 and formulation-stability/release tests were reported. | Male C57BL/6 mice (5–7 weeks); acute 2.5% DSS for 7 days followed by 3 days of recovery; n=5/group | Beginning on day 2, three oral doses at 3-day intervals: EV or coated EV formulations at 1×10^11 particles/mouse; free or encapsulated 5-ASA at 2.5 mg/mouse | Oral |
| DOI 10.3389/fmicb.2026.1876636 [main-text Ref 82] | EVs from live versus pasteurized Akkermansia muciniphila; bacterial/postbiotic EV product | Live EVs: culture supernatant 10,000×g 30 min, 0.22-µm filtration, 170,000×g 60 min, PBS wash/re-spin. Pasteurized product: bacterium and supernatant heated at 70 °C for 30 min before the same isolation; TEM/NTA and markers/composition characterized. | Male C57BL/6 mice (6–8 weeks); acute 2% DSS for 5 days; four groups | 20 µg EVs in 150 µL PBS by daily oral gavage from day 1; euthanasia day 7 | Oral gavage |
| DOI 10.1186/s12951-026-04866-5 [main-text Ref 81] | Bletilla striata-derived extracellular vesicles displayed on engineered Escherichia coli Nissle 1917 (EcN@BEVs) | Peeled B. striata rhizomes were cut, homogenized in cold PBS (1:4), and cleared at 1,000×g for 20 min, 5,000×g for 40 min and 10,000×g for 1 h; the resulting BEVs were stored at −80 °C. EcN and BEVs were separately functionalized with 1 mM DSPE-PEG-β-cyclodextrin or DSPE-PEG-adamantane for 1 h at 37 °C, combined for 30 min and washed twice. TEM/NTA/zeta potential and targeted flavonoid metabolomics were reported; no further BEV purification step or concentration was stated. | Male BALB/c mice (6–8 weeks); acute 3% DSS for 6 days; five groups, n=5 | Methods report oral PBS, EcN (1 × 10^8 CFU/day), BEVs (100 µL/day), or EcN@BEVs (1 × 10^8 CFU/day) for 4 days, with euthanasia on day 11. The results narrative instead describes 5 × 10^8 CFU daily for 5 days; this internal dosing discrepancy is unresolved. Supplementary Figure S7 reports 50 and 100 µg/mL BEVs for an HT-29 cell-viability experiment, but does not state the concentration of the 100-µL in-vivo BEV dose. | Oral gavage |
| DOI 10.1016/j.apsb.2026.07.044 [main-text Ref 61] | Portulaca oleracea-derived extracellular-vesicle-like particles (PO-EVLPs) carrying plant miR159-y | Fresh P. oleracea was washed, cut and homogenized in PBS; juice was cleared at 4,000×g for 1 h and 10,000×g for 1 h, followed by ultracentrifugation at 120,000×g for 2 h and PBS resuspension. NTA, TEM, TLC, SDS-PAGE and multi-omics were used; 1 mg corresponded to approximately 5.66×10^8 particles. | Acute 3% DSS colitis for 1 week; chronic 2% DSS (three cycles); AOM/DSS colitis-associated-cancer model; intestinal innate-lymphoid-cell assays | PO-EVLPs at 30 or 60 mg/kg by daily oral gavage during modeling; biodistribution used 30 mg/kg. Separate mechanistic arms used miR159-y agomir (500 pmol or 1 nmol, intraperitoneal) and miR159-y antagomir. | Oral gavage; intraperitoneal oligonucleotide perturbation |
| Tang et al. 2024; PMID 39583323 [main-text Ref 30] | Human PDLSC EVs after no conditioning (P-EVs) or 24-h LPS conditioning at 1 microg/mL (LPS pre-EVs) | P2-P6 PDLSCs were conditioned for 24 h, followed by 48 h in 5% EV-depleted FBS; supernatant was cleared of cells/debris and the article's workflow was used for EV recovery. TEM, NTA, and western blotting for TSG101, CD9, CD81, and calnexin were reported. | Female C57BL/6 mice (7 weeks, 18-20 g); acute 3% DSS colitis; n=6/group | P-EVs or LPS pre-EVs in 200 microL were injected on days 1, 3, and 5; the administered particle/protein amount was not stated in the article text; euthanasia on day 7 | Tail-vein injection |
| Liang et al. 2023; PMID 37256205 [main-text Ref 95] | Native/unmodified hUC-MSC exosomes | TEM, NanoFCM size distribution, and exosomal-marker western blotting were reported; detailed isolation information is provided in the article supplement. | Male C57BL/6 and BALB/c mice (6-8 weeks); acute 2% DSS and TNBS colitis; TNF-alpha-injured intestinal organoids | DSS: 300 microg in 200 microL PBS intraperitoneally every other day from day 4; TNBS: 300 microg on days 1 and 3; organoids: 100 microg/mL for 48 h; Wnt-C59: 10 mg/kg orally for 5 days | Intraperitoneal; ex vivo organoid |
| Feng et al. 2026; PMID 41773922 [main-text Ref 96] | iPSC-MSC-derived exosomes; native, miR-34a-5p-overexpressing (Exo-OE), and miR-34a-5p-knockdown (Exo-KD) preparations | The accessible abstract identifies microarray-guided miR-34a-5p gain/loss preparations; isolation and characterization details are reported in the full article but are not stated in the abstract. | TNBS-induced colitis in mice; colonic Lgr5/Bmi1/Ki-67 and immune-cell readouts; patient CD LPMCs | Single intraperitoneal administration; benefit followed for 7 days; administered amount was not stated in the accessible abstract | Intraperitoneal; ex vivo patient LPMC assays |

**Table S5B. Comparators, mechanistic evidence, outcomes, interpretation, and limitations**

Study order is identical to Table S5A so that product/design information and evidence interpretation remain linked. Records designated as 'Supplementary evidence ledger' are included for completeness and are not cited in the main-text narrative or reference list.

| **Study / main-text linkage** | **Principal comparators** | **Mechanistic/perturbation evidence** | **Main outcome** | **Evidence interpretation** | **Major limitation** |
| --- | --- | --- | --- | --- | --- |
| BM-MSC EVs - Cao et al. (24) | Healthy control; DSS plus saline; DSS plus EVs. In vitro: control, LPS, LPS plus EVs (100 microg/mL after 30-min LPS pre-stimulation), and EV-only groups. | PKH67 uptake, macrophage-state markers, cytokines, and JAK1/STAT1/STAT6 phosphorylation were measured. No pathway inhibitor, knockdown, macrophage depletion, or cargo loss-of-function test was performed. | EVs improved weight loss, DAI, colon shortening, gross and histological injury, MPO activity, inflammatory mediators, and M1/M2-associated readouts. | Therapeutic association with macrophage-state and JAK/STAT readouts; the study does not establish pathway or macrophage dependence. | Acute concurrent-treatment DSS model; no particle-number dose, purity-negative marker, cargo dependency, cell-specific dependency, chronic model, or human validation. |
| BMSC-derived exosomes carrying TSG-6 - Wu et al. (25) | In vivo: healthy/PBS, DSS/PBS, and DSS plus BMSC exosomes. In vitro: untreated/LPS controls, BMSC exosomes, control-siRNA exosomes, and exosomes from siTSG-6-transfected BMSCs. | PKH26 uptake and pyroptosis/barrier readouts were assessed. Donor-cell siRNA reduced exosomal TSG-6 and weakened suppression of NLRP3, cleaved caspase-1/GSDMD-N, IL-1beta, and IL-18 in LPS-treated Caco-2 cells. The TSG-6 loss-of-function experiment was in vitro; the mouse study did not test TSG-6 depletion. | BMSC exosomes improved weight, DAI, colon length, spleen index, and histology; reduced NLRP3/caspase-1/GSDMD-associated pyroptosis and IL-1beta/IL-18; and increased ZO-1 and occludin. TSG-6-depleted exosomes showed reduced anti-pyroptotic activity in vitro. | Supportive preparation-specific evidence linking BMSC exosomes to epithelial-pyroptosis and barrier readouts, with in-vitro cargo perturbation supporting a TSG-6 contribution; it does not establish in-vivo TSG-6 dependence or direct TSG-6-NLRP3 binding. | Acute DSS model with n=5/group; dose reported by protein mass with per-injection versus cumulative wording unclear; no particle-number dose or negative purity marker; transformed human Caco-2 line; no in-vivo TSG-6 loss-of-function, chronic model, or human validation. |
| hUC-MSC EVs - miR-326 EVs (26) | Healthy/PBS; DSS/PBS; native hUC-MSC exosomes; exosomes from cells transfected with miR-326 mimic, inhibitor, or matched negative controls. In vitro, MLN4924 served as a neddylation-pathway comparator. | miR-326 gain/loss arms, a NEDD8 wild-type/mutant 3-prime-UTR luciferase assay, and NEDD8-cullin1 co-immunoprecipitation support a miR-326/NEDD8-neddylation link. No in-vivo rescue with miR-326-resistant NEDD8 was reported. | Exosome treatment improved clinical and histological colitis measures and inflammatory readouts; miR-326 enrichment strengthened, whereas miR-326 inhibition weakened, these effects together with reduced neddylation-related signaling. | Cargo-manipulation and target-binding evidence support a contributory miR-326/NEDD8 mechanism, but do not establish that this cargo is the sole active EV component. | Acute mouse model, high intravenous protein dose, precipitation-based isolation, and no human mucosal or target-rescue validation. |
| hUC-MSC EVs - miR-129-5p EVs (27) | Healthy/PBS; DSS/PBS; native hUC-MSC exosomes; miR-129-5p mimic, inhibitor, and matched negative-control exosome groups. | miR-129-5p gain/loss arms and ACSL4 wild-type/mutant 3-prime-UTR luciferase testing support direct targeting; ferroptosis-associated iron, GPX4/GSH, DMT1, COX2, and ACSL4 readouts were measured. No ACSL4 genetic rescue was reported. | Exosomes reduced colitis injury and ferroptosis-associated changes; miR-129-5p enrichment enhanced and inhibition attenuated the reported benefit. | The data support a contributory miR-129-5p/ACSL4-ferroptosis axis, not exclusive cargo causality. | Very high intravenous dose, acute preclinical model, transfection-based cargo manipulation, and no ACSL4 rescue or human mucosal validation. |
| Naive and cytokine-primed MSC-EVs versus parental MSCs - Tolomeo et al. (34) | Healthy untreated; DSS plus PBS; DSS plus naive MSCs; DSS plus cytokine-primed MSCs; DSS plus naive EVs; DSS plus cytokine-primed EVs. | Direct source-cell/product and donor-conditioning comparisons were combined with macrophage assays, intestinal iNOS/CD163 and Nos2/Arg1 readouts, lymph-node Treg/Teff ratio, collagen, CD31/angiogenic markers, and MUC5AC. No cargo depletion, receptor blockade, or rescue experiment established pathway dependence. | Naive and primed MSCs worsened clinical and histological colitis. Naive EVs showed only a nonsignificant trend, whereas primed EVs improved weight, DAI, histological injury, macrophage polarization, Treg/Teff ratio, fibrosis, angiogenesis, and MUC5AC expression. | Counter-evidence against assuming that MSCs and their EVs share effects, and comparative evidence that donor conditioning can determine EV activity. The findings support product- and preparation-specific interpretation rather than a general MSC-EV class effect. | Acute DSS model with n=5/group; EV enrichment by ultrafiltration without a higher-resolution purification step; cell and EV schedules were not exposure-equivalent; no causal cargo/pathway perturbation, chronic model, pharmacokinetics, or human validation. |
| Unloaded bovine milk EVs - Seegobin et al. (35) | Route-matched dPBS negative control and ciclosporin 70 mg/kg/day positive control; oral and rectal arms. Liposomes served as a positive control for the laurdan stability assay. | Route comparison and simulated saliva/gastric/intestinal-fluid experiments directly tested delivery stability; laurdan and lipophilic/hydrophilic model cargos distinguished bilayer damage from apparent particle stability. No molecular dependency experiment was performed. | Unloaded milk EVs did not improve DAI, colon measures, histology, or inflammatory outcomes by either route; oral EVs significantly worsened weight loss and DAI versus PBS. Ciclosporin showed the expected oral benefit. | Preparation-specific negative evidence against intrinsic efficacy and conventional oral delivery of this unloaded milk-EV product; it does not exclude protected or therapeutically loaded milk-EV formulations. | Single acute female-mouse model and one milk preparation; findings may vary with sex, strain, processing, cargo, and colon-targeting technology. |
| Milk EVs - anti-TNF-alpha nanobody / LL37 (67) | PBS/DSS controls; unloaded milk EVs; free VHH or LL37; VHH-loaded, LL37-loaded, and dual-loaded EV formulations. | Single-cargo and dual-cargo arms partly separate anti-TNF and antimicrobial/barrier contributions; cytokine, barrier, microbiome, and antimicrobial readouts were measured. No TNF rescue, receptor blockade, or compartment-specific cargo-dependence test was performed. | Loaded formulations improved acute and chronic DSS outcomes; the dual VHH/LL37 product generally showed the broadest anti-inflammatory, barrier, and microbiota-associated effects. | Comparator structure supports combined formulation activity, while the relative contributions of encapsulated versus surface-bound cargo and endogenous milk-EV cargo remain uncertain. | High EV-protein dose; TAT-assisted loading and residual surface cargo; milk/casein lot variability and allergenicity; chronic schedule wording and repeat-dose translation remain uncertain. |
| Grape EV-like nanoparticles (7) | PBS vehicle; GELNs; GELN-derived lipids; reassembled lipid nanoparticles; beta-catenin inhibitor carnosic acid; lipid-pathway inhibitor propranolol; untreated crypt/organoid controls. | GELN uptake by Lgr5-positive cells, lipid reassembly experiments, Tcf/LEF reporters, and partial reversal by beta-catenin or phosphatidic-acid-pathway inhibition support a lipid/Wnt-associated stem-cell response. No in-vivo Lgr5-cell ablation or cargo-depletion rescue was reported. | Oral GELNs increased Lgr5-positive/Ki67-positive stem-cell and repair readouts, improved DSS-associated tissue injury, and delayed mortality under continued DSS exposure. | Perturbation evidence supports a lipid-associated Wnt/beta-catenin branch, but does not prove that intact GELNs or a single lipid species is necessary and sufficient for protection. | Older preparation with incompletely restated centrifugation settings; severe concurrent DSS model; no chronic, human-mucosal, or cell-dependency validation. |
| Broccoli-derived nanoparticles (8) | PBS/colitis controls; BDNs; wild-type versus AMPK-alpha1-deficient dendritic cells/mice; BDN lipids; sulforaphane-depleted and sulforaphane-reconstituted liposome-like particles; DC adoptive-transfer controls. | Loss of protection with AMPK-deficient DCs, adoptive transfer of BDN-conditioned DCs, and sulforaphane depletion/reconstitution provide pathway- and cargo-oriented perturbation evidence. | BDNs reduced DSS, T-cell-transfer, and anti-CD40 colitis readouts and shifted DC/T-cell cytokine phenotypes toward a more tolerogenic profile. | This is comparatively strong preclinical evidence for DC AMPK dependence and a sulforaphane-related contribution, although the complete active composition remains unresolved. | Predominantly preventive dosing, plant-mixture attribution, mouse-focused evidence, and no human intestinal DC or mucosal validation. |
| Ginger-derived nanoparticles (9) | Healthy/PBS and disease controls across DSS, DSS-withdrawal, IL-10-knockout, and AOM/DSS models; GDNP2 treatment; uptake/exposure experiments. | Colon exposure, epithelial/macrophage uptake, cytokine, barrier, survival, and compositional readouts were measured, but no cargo-depleted GDNP2, receptor knockout, or recipient-cell loss-of-function experiment was reported. | GDNP2 improved inflammatory injury and tissue-repair readouts across acute, spontaneous, and cancer-associated models and reduced tumor burden in AOM/DSS studies. | Broad model replication supports preparation-level activity; specific lipid, RNA, or phytochemical causality remains associational. | Multicomponent plant fraction, prevention-heavy schedules, limited active-comparator structure, and no human mucosal or chronic-product safety validation. |
| Turmeric-derived nanovesicles - Gao et al. (Ref 10) | Healthy control; DSS/PBS; 5-ASA 100 mg/kg; TNVs 10 mg/kg. Chronic studies used the same four group types; in vitro assays included blank, inflammatory stimulus, and TNV groups. | Macrophage polarization, epithelial tight-junction, biodistribution, microbiome, proteomic/lipidomic, and curcuminoid readouts were measured. No curcumin-depleted TNV, cargo rescue, or recipient-cell genetic dependency test was reported. | TNVs improved acute and chronic colitis indices, inflammatory and barrier readouts, macrophage-state markers, and microbiome-associated measures, with efficacy compared against 5-ASA. | Separate preparation-level evidence supports oral TNV activity, but the proposed curcuminoid-macrophage-microbiota-barrier chain is not proven as a causal sequence. | Plant-mixture attribution, curcuminoid-equivalent exposure uncertainty, model-specific chronic dose not restated, and no human validation. |
| Turmeric-derived exosome-like nanovesicles - Liu et al. (Ref 44) | Healthy and DSS controls; TDNP1 versus TDNP2; vehicle; in uptake studies, endocytosis inhibitors; NF-kappaB reporter experiments. | Fraction comparison, uptake inhibition, reporter activity, and curcumin/compositional assays support preparation-specific bioactivity. No curcumin-depleted TDNP2, rescue, or recipient-cell genetic dependency test was performed. | TDNP2 reduced acute DSS inflammation and enhanced post-DSS mucosal healing, with associated macrophage, barrier, and pro-resolving readouts. | The evidence supports TDNP2-level activity and NF-kappaB-associated effects, not direct proof that curcumin or any single cargo mediates efficacy. | Dose reported per mouse rather than by body weight; differential-spin settings not restated; acute/preclinical evidence and multicomponent-cargo uncertainty. |
| Momordica charantia-derived EVs - Gao et al. (54) | Healthy/PBS; DSS/PBS; MCEVs 15 or 30 mg/kg; dose-response, safety, and biodistribution experiments. RNase and proteinase-K component-depletion conditions were used in vitro. | In-vitro nuclease/protease perturbation suggests that multiple molecular fractions contribute; antioxidant, inflammatory, barrier, microbiome, metabolomic, and biodistribution readouts were measured. No in-vivo component depletion or recipient-cell dependency test was reported. | The 30-mg/kg dose consistently improved weight, DAI, colon injury, mucus/barrier, inflammatory/oxidative, immune-cell, microbiome, and metabolite readouts; 15 mg/kg effects were less consistent. | Dose-response and in-vitro component perturbation support multicomponent preparation activity, but the proposed microbiome/metabolite/barrier mechanism remains only partly causal. | Short acute model, no established-drug comparator, high plant-EV protein dose, DSS concentration not explicit in the published methods, and no human validation. |
| Engineered PELNs - HA-modified red cabbage particles (11) | Healthy; DSS; sulfasalazine 100 mg/kg/day; low- and high-concentration Rabex; low-concentration HA-modified t-Rabex. Uptake assays included CD44-blocking antibody and endocytosis inhibitors. | A low-dose t-Rabex versus high-dose unmodified Rabex comparison and partial reduction of uptake by CD44 blockade support a targeting contribution; no CD44 knockout or in-vivo receptor-dependency test was performed. | Rabex improved colitis outcomes dose-dependently; low-concentration t-Rabex increased cell engagement and achieved efficacy comparable to or better than the higher unmodified concentration. | The formulation comparison supports an HA-associated targeting advantage, but receptor dependence and the contribution of unmodified particle cargo are not fully resolved. | Supplementary cost analysis defines a nominal 5 × 10^10-particle dose, but the in-vivo protocol reports concentrations without gavage volume; per-animal particle exposure therefore remains uncertain. Other limitations include a modest blockade effect, label-based biodistribution, no chronic or human validation, and incomplete repeat-dose/off-target safety assessment. |
| Checkpoint EVs - PD-L1 / miR-27a-3p EVs (6) | Healthy/PBS; DSS/PBS; native/normoxic, mock-engineered, primed, and dual PD-L1/miR-27a-3p EVs. Mechanistic controls included PD-1 blockade, miR-27 inhibitor, wild-type/mutant PHB1 3-prime-UTR reporters, and transcriptional blockade; patient PBMCs and colonoids were tested ex vivo. | PD-1 blockade reversed T-cell effects, whereas miR-27 inhibition and PHB1 reporter assays support the miR-27a-3p/PHB1 branch. These orthogonal tests support two contributory mechanisms, although donor priming and other EV cargo remain potential contributors. | Dual-engineered EVs improved murine DSS and humanized TNBS colitis readouts and rebalanced T-cell/barrier phenotypes in patient-derived experimental systems. | Mechanistic perturbation and human-relevant ex-vivo evidence are substantial, but the product has not been tested clinically and should not be described as proving durable immune reprogramming. | Supplementary methods report serial centrifugation and 100,000 × g ultracentrifugation, but individual spin durations are not stated. Other limitations include mixed priming/engineering effects, an acute humanized model, a short safety window, and unresolved repeat-dose immunosuppression risk. |
| Biomaterial-retained MSC EVs - Gan et al. (71) | Healthy saline control; DSS plus saline; DSS plus free MSC exosomes; DSS plus alginate-encapsulated exosomes (AE); DSS plus multilayer enteric microcapsules (EGAE). Biodistribution additionally compared free exosomes, AE, gelatin-AE, and EGAE. | Stepwise formulation comparators and GI-fluid/release/biodistribution studies support protection and colon retention. In vitro macrophage-state, epithelial migration, TEER, and cytokine assays support biological activity, but no cell-specific loss-of-function or cargo-depletion test was performed. | EGAE produced greater colonic retention and the strongest improvements in weight, DAI, colon length, histology, MPO, epithelial coverage, and inflammatory/repair markers. | Strong formulation-retention-function evidence for oral delivery; not proof of a specific exosomal cargo or recipient-cell dependency. | Very high protein dose, acute 5% DSS model, small groups, no empty multilayer-microcapsule efficacy control, complex manufacturing, and no repeat-dose or human validation. |
| Gal-IL10-EVs (C/A) - Liu et al. (64) | Healthy PBS; DSS plus PBS; DSS plus naive EVs; DSS plus IL10-EVs; DSS plus Gal-IL10-EVs. In vitro uptake compared EVs, Gal-EVs, and free-galactose-preblocked Gal-EVs. | Naive-EV and IL10-EV comparators partly separate donor cargo and galactose targeting; free galactose reduced Gal-EV uptake, supporting a receptor-associated uptake component. No MGL knockout/blocking antibody, IL-10 neutralization, free IL-10 comparator, or empty-hydrogel efficacy arm was included. | Gal-IL10-EVs gave the greatest improvement in weight loss, DAI, colon length and histology and reduced inflammatory cytokines; coated particles accumulated in the colon and showed favorable short-term safety. | Comparator and competition evidence supports, but does not prove, contributions from IL-10 loading and galactose-associated macrophage uptake. | Small acute model; complex HEK293T-derived product; hydrogel and free-IL-10 contributions are incompletely separated; no receptor/cargo dependency test, chronic safety, or human validation. |
| MSC-IL-27 EV microcarriers - Nie et al. (72) | Healthy; DSS/PBS; empty adhesive G-DM particles; native MSC EVs; IL-27-high MSC EVs; and IL-27-high EV-loaded adhesive particles. In vitro, epithelial and macrophage inflammatory conditions were included. | Stepwise EV/cargo/formulation comparisons, adhesion and sustained-release testing, uptake, cytokine, and tight-junction readouts support combined contributions. No IL-27 neutralization, receptor blockade, cargo depletion, or cell-specific loss-of-function test was reported. | IL-27-high EVs improved colitis and barrier/inflammatory readouts, and the bioadhesive microcarrier produced the strongest retention and therapeutic response. | The comparator ladder supports formulation-enhanced delivery and an IL-27-enrichment association, but not IL-27 or recipient-cell dependence. | Small acute rat groups, therapeutic per-rat EV dose not explicit, formulation/cargo effects incompletely separated, and no chronic or human validation. |
| Milk EVs carrying TNF-alpha siRNA - Han et al. (68) | Healthy/saline; DSS/saline; unloaded milk exosomes; low- and high-dose siRNA-loaded products. In vitro controls included scrambled siRNA, free siRNA, non-electroporated mixtures, and alternative exosome preparations. | Scrambled, free-siRNA, and physical-mixture controls plus TNF-alpha mRNA/protein suppression support functional siRNA delivery. No rescue with siRNA-resistant TNF-alpha was performed. | Loaded milk exosomes reduced TNF-alpha and improved inflammatory, histological, and clinical colitis outcomes after intraperitoneal or oral dosing; unloaded EVs were less effective. | The control structure supports sequence-specific siRNA activity, although endogenous milk-EV cargo and route-specific contributions remain. | Very high particle doses, electroporation/lot variability, milk allergenicity, route heterogeneity, and limited repeat-dose safety and human-dose translation. |
| Protein-hybrid EVs - Lee et al. (69) | Healthy/PBS; DSS/PBS; 5-ASA; native PeoEVs; free or hybrid cyclic immunoregulatory protein in organoid/cell assays; PeoEV and Cyc@PeoEV animal arms. | Acid/protease stability, transwell delivery, pSTAT1/pSTAT6, organoid-barrier, and macrophage-conditioned-medium experiments support functional protein delivery. No cargo-depleted vesicle, target-gene knockout, or in-vivo rescue was reported. | Cyc@PeoEV improved DSS-associated weight, colon, histology, barrier, organoid, and inflammatory readouts more strongly than native PeoEVs. | The hybrid-versus-carrier comparison supports added protein/formulation activity, but intracellular target engagement and exclusive cargo dependence are not established. | Methods/figure DSS-concentration discrepancy; protein dose not expressed per kg; ExoQuick cleanup; acute model and no human validation. |
| PrEXO-a23 hybrid vesicles - Cao et al. (65) | Healthy/PBS; DSS/PBS; PrEXO carrier; free anti-IL-23; rEXO-a23; and PrEXO-a23. In vitro studies included exosome, platelet-vesicle, physical-mixture, cleavable versus non-cleavable linker, and MMP-inhibitor controls; CAC experiments included p53 inhibition. | Stepwise carrier/antibody controls, MMP-cleavable versus non-cleavable linker, GM6001-sensitive release, dual-delivery imaging, and attenuation by pifithrin-alpha provide formulation and pathway perturbation evidence. | PrEXO-a23 improved acute colitis, fibrosis-associated, and AOM/DSS tumor outcomes and altered DC/Treg and IL-23-related readouts more strongly than single-component controls. | The study offers strong formulation-release-function evidence and supports a p53-associated antitumor branch, but does not prove a single recipient-cell or exosomal-cargo dependency. | Complex multicomponent manufacture, intravenous route, incomplete attribution among rEXO/platelet/antibody effects, and no direct human-mucosal or biologic-comparator validation. |
| MSC exosomes - perianal CD fistula phase I (33) | Single-arm, open-label phase-I study; no vehicle, placebo, standard-of-care, or active comparator group. | Clinical feasibility, local safety, fistula closure, and MRI outcomes were observed. No mechanistic perturbation, biodistribution, or exposure-response analysis was included. | Four of five patients met the reported response criterion, including three complete healings; one patient did not respond. No local or systemic adverse event was reported during 6 months. | This provides preliminary local safety and feasibility evidence only; efficacy cannot be separated from natural history, procedures, or concomitant care. | n=5, uncontrolled and non-randomized; refractory perianal fistula indication only; prior/concurrent management may confound outcomes; no inference to luminal UC/CD or long-term safety. |
| Liu et al. 2026; PMID 41983055 [main-text Ref 56] | Healthy; DSS+PBS; vesicles; whole decoction; vesicle-depleted decoction; antibiotic-depleted pseudo-germ-free cohorts | Antibiotic depletion abolished most benefit and bacterial uptake was shown, supporting microbiota dependence; no cargo depletion/rescue experiment | Improved DAI, colon length/histology, cytokines and barrier markers; altered microbiota | Supports a microbiota-dependent therapeutic branch, not a complete vesicle-cargo mechanism | Acute model; complex decoction vesicles and co-isolates; microbiota shifts may partly be secondary; no human validation |
| Wu et al. 2026; PMID 41639673 [main-text Ref 57] | Vehicle, 5-ASA, dose range, antibiotics, FMT, IAA, AhR antagonist, L. paracasei mono-colonization and organoids | Convergent perturbations support microbiota→tryptophan/IAA→AhR signaling with downstream ROS/NLRP3 modulation; AhR antagonism reduced benefit | Reduced inflammation/oxidative stress and improved barrier and organoid outcomes | Relatively strong preclinical dependency evidence for the microbiota-metabolite-AhR branch | Acute model; intact-vesicle/cargo attribution, chronic safety and human validation unresolved |
| Xiao et al. 2025; DOI 10.24976/Discov.Med.202537199.144 [main-text Ref 104] | PBS; native iPSC-MSC EVs; Baf-A1-conditioned EVs; donor-cell secretion/reuptake comparisons | 1 nM Baf-A1 reduced parental-cell EV reuptake and increased yield; conditioned EVs retained activity comparable to native EVs | Higher EV output with broadly comparable efficacy in colitis and liver-injury models | Manufacturing/process evidence; it does not demonstrate superior efficacy, scale-up or batch equivalence | EV dose not stated; prophylactic IV schedule; acute models; donor-conditioning may alter cargo; no long-term safety |
| PMID 42548959 [main-text Ref 59] | Fresh GEVs; thermally reassembled GEVs; each with/without TNF-α siRNA; uptake inhibitors | Multiple clathrin-pathway inhibitors reduced enhanced cellular uptake; NLRP3 and cytokine changes support anti-inflammatory activity, but no in-vivo NLRP3 dependency test | Thermal EVs improved intestinal uptake and intrinsic efficacy; siRNA-loaded thermal EVs performed best | Defined processing–uptake relation plus formulation efficacy; inflammatory-pathway evidence remains partly associative | Commercial isolation kit and heat-induced product-identity changes; single acute model; chronic safety/systemic exposure unresolved |
| PMID 42544276 [main-text Ref 60] | Healthy; DSS+PBS; MFELNs; LPS cell controls; no established-drug comparator | Macrophage-state, microbiota and SRC/NF-κB readouts plus docking/bioinformatics; no inhibitor, cargo-depletion or rescue test | Improved DAI, histology, cytokines, barrier-associated and microbial outcomes | Therapeutic association only; the proposed compound/SRC/NF-κB mechanism is hypothesis-generating | Acute model; individual active components and microbial metabolites unresolved; decoction co-isolates, chronic safety and human validation |
| PMID 42468020 [main-text Ref 75] | PNHA alone; PNHA–5-ASA; PNHA–ProEV; combined PNHA–5-ASA–ProEV; PBS; live VSL#3 route comparisons | Retention/release, barrier, cytokine and microbiota readouts; no pathway-specific perturbation | Combined formulation gave the broadest mucosal recovery and homeostasis benefit | Good component/formulation comparison, but synergy and molecular mechanism remain incompletely separated | Exact per-rat formulation dose is confined to unavailable Supplementary Table S2; complex manufacture, bacterial-EV safety and no human validation |
| PMID 42439691 [main-text Ref 29] | Ethanol control; TNBS+PBS; untreated-macrophage exosomes; IL-33-exosomes; XAV939 inhibitor | Pharmacologic Wnt/β-catenin inhibition with XAV939 reduced the exosome benefit in vivo; DKK1/XAV939 also used in vitro | Reduced TNBS injury and improved epithelial-barrier outcomes | Supports Wnt/β-catenin dependence at pharmacologic level; responsible EV cargo is not identified | Acute TNBS model; PEG-based isolation; no genetic dependency, specific cargo assignment or human validation |
| PMID 42432695 [main-text Ref 58] | Healthy and DSS controls; low/high R-ELNs; R-ELN versus A-ELN comparison; pathway activators/inhibitors in macrophage assays | R-ELNs shifted macrophages from M1- toward M2-associated states and reduced AKT and p38 phosphorylation. AKT/p38 activators attenuated the cellular benefit, whereas inhibitors produced no further reduction. Secondary macrophage-exosome miRNA profiling and luciferase assays support target interactions, but EV-cargo necessity was not tested in vivo. | Improved weight/DAI, colon length, histology, inflammatory and barrier indices; altered microbiota-associated outcomes | Supports AKT/p38 involvement at the cellular pharmacologic level and a downstream macrophage-exosome hypothesis; it does not establish an in-vivo cargo-dependent chain | Predominantly prophylactic acute model; pathway dependency is cell-based; secondary-exosome/cargo causality and human relevance remain unresolved |
| PMID 42390648 [Supplementary evidence ledger; not cited in main text] | Healthy, DSS and DSS+BMSC-exosome groups; miR-148a-3p mimic/inhibitor, Ets-1 knockdown/overexpression and dual-luciferase cellular experiments | The miR-148a-3p inhibitor partly reversed exosome effects; Ets-1 knockdown rescued inhibitor-associated changes, Ets-1 overexpression attenuated mimic effects, and luciferase assays supported direct targeting. These perturbations were cellular rather than in vivo. | Reduced DSS injury and macrophage inflammatory/pyroptosis-associated readouts | Strong cellular miR-148a-3p→Ets-1 cargo-target evidence, but no in-vivo cargo-depleted EV or antagomir dependency experiment | Acute DSS model; one EV dose; incomplete reversal suggests additional cargo; long-term safety, off-target effects and human validation remain unresolved |
| PMID 42378030 [Supplementary evidence ledger; not cited in main text] | Vehicle, low/high exosome dose, in-silico miRNA targets and cellular uptake; no exosome-depleted milk control | let-7g/TLR4–NF-κB and miR-148a/NLRP3 links are based on abundance, prediction/expression and uptake rather than cargo loss/rescue | Improved inflammation, antioxidant/barrier indices and microbiota composition | Compatible with miRNA and microbiota mediation but does not establish intact-delivery or specific-cargo necessity | Prophylactic acute model; milk-matrix contribution, digestion/allergenicity, cargo dependency and human validation unresolved |
| PMID 42376871 [main-text Ref 76] | Uncoated oral EVs; coated oral EVs; IV EVs; coated empty liposomes; healthy/DSS controls | GI-fluid/enzyme release and coating controls show protection and pH-responsive delivery; no cell-specific receptor perturbation | Only coated oral EVs consistently improved DAI/colon outcomes; effect exceeded uncoated oral and was comparable or better than IV EVs | Strong formulation and route comparison supporting colon delivery, without defining a molecular therapeutic mechanism | Small groups; acute model; complex coating/batch comparability and repeat-dose safety unresolved |
| PMID 42160026 [main-text Ref 77] | Vehicle; uncoated EVs; CS@EVs@TA; healthy/DSS controls | Coating stability, ROS scavenging, colon accumulation, 16S/SCFA and barrier readouts; no microbiota depletion/FMT or component-only CS/TA arms in vivo | Reduced inflammation/oxidative injury and improved barrier and microbiota/SCFA profiles | Engineered-platform efficacy with plausible multi-component actions; EV versus coating contributions and microbiota causality remain unresolved | No standard-drug comparator; acute model; coating optimization, large-animal/clinical validation and manufacturing consistency needed |
| PMID 42107777 [main-text Ref 78] | Parent garlic, B. thetaiotaomicron and A. muciniphila EVs; two hybrid EVs; vehicle; prevention and therapeutic schedules | Hybrid GB EVs raised epithelial pIgR/SIgA; p-STAT6 inhibitor AS1517499 blocked pIgR induction in vitro | GB EVs were strongest across colitis, barrier, mucosal-immunity and microbiota outcomes | Supports STAT6-dependent pIgR induction in vitro and therapeutic efficacy in vivo; in-vivo pIgR necessity is untested | Complex fused identity; inhibitor evidence is cell-based; chronic model efficacy and human validation remain limited |
| PMID 42034038 [main-text Ref 79] | Vehicle; P. distasonis EVs; AhR inhibitor; microbiome/tryptophan-metabolite analyses | AhR inhibition diminished benefit; I3A/IPA and microbiota changes align with a microbiota–tryptophan–AhR branch | Reduced colitis severity and improved barrier/inflammatory outcomes | Perturbation-supported AhR involvement, though inhibitor specificity and EV cargo source remain uncertain | Prophylactic acute model; composition and bacterial-EV safety unresolved; no human validation |
| PMID 42030800 [Supplementary evidence ledger; not cited in main text] | Vehicle; mesalazine; low/high EV doses; antibiotic depletion; microbiota-metabolite transfer; RSL3, MK2206 and SC79 cell perturbations | Antibiotic depletion removed significant benefit; transferred microbial metabolites retained activity; AKT inhibitor/agonist bidirectionally modulated ferroptosis readouts in vitro | Improved UC-like indices, barrier and microbiota/metabolite profiles; suppressed ferroptosis | Convergent evidence supports microbiota dependence and an AKT/mTOR–ferroptosis branch, strongest in vitro | Acute model; pathway perturbation not demonstrated genetically/in vivo; microalgal product standardization and human validation needed |
| PMID 41926795 [Supplementary evidence ledger; not cited in main text] | PBS; HFL1-sEV; miR-320d mimic/inhibitor; target-site luciferase and RNA-binding assays | miR-320d mimic/inhibitor, dual-luciferase and RIP/MeRIP assays support targeting of ALKBH5/YTHDF2 and altered m6A stability/expression of BCAT2 | Reduced macrophage ferroptosis/inflammation and DSS disease severity | Detailed cellular cargo–target evidence, but in-vivo EV/cargo necessity was not established with depleted EVs or antagomir rescue | Very high IV EV dose (1 mg/injection), acute model, prior-method isolation detail, no human validation |
| PMID 41875611 [Supplementary evidence ledger; not cited in main text] | Vehicle; 5/10 mg/kg doses; antibiotic-depleted/FMT cohorts; cellular inflammatory controls | FMT transferred part of the phenotype; TLR4/MyD88–NF-κB and macrophage-state changes were measured without receptor/pathway blockade | Reduced inflammation and apoptosis; improved mucus/tight junctions, SCFAs and microbiota | Microbiota contribution is supported by FMT, whereas the proposed TLR4/NF-κB mechanism and multi-cargo attribution remain associative | Prophylactic design; fluorescence localization and multi-omics do not establish active cargo; acute model and no human validation |
| PMID 41865696 [main-text Ref 80] | Free budesonide; unloaded CN-EVLP; loaded CN-EVLP–Bud; vehicle | Component comparison supports carrier contribution to uptake and M1/M2 shift; authors explicitly state NF-κB/STAT6 inhibition and tissue-level macrophage validation are still needed | Loaded product outperformed free budesonide and unloaded particles across several UC outcomes | Relevant free-drug and carrier controls support formulation benefit, but the polarization mechanism is not causal | Group description is internally incomplete; residual-free-drug control, source variability, chronic safety and human validation unresolved |
| PMID 41865564 [main-text Ref 74] | Healthy and DSS/PBS controls; uncoated EVs; free 5-ASA; coated EVs without drug (CEV); dual-coated EVs containing 5-ASA (DCEV) | Component/formulation comparisons, pH-dependent coating stability and release, transepithelial uptake and macrophage delivery support colon-targeted delivery. Tight-junction, cytokine and NF-κB readouts were measured without pathway-specific perturbation. | DCEV produced the broadest improvements in colitis, epithelial-barrier and inflammatory outcomes | Strong formulation and component-comparator evidence for oral delivery; molecular mechanism and pharmacologic synergy are not causally established | Acute model; high particle dose; fluorescent-dye transfer and formulation/batch effects require control; repeat-dose safety, scale-up/GMP and human validation unresolved |
| DOI 10.3389/fmicb.2026.1876636 [main-text Ref 82] | Healthy; DSS+PBS; live-bacterium EVs; pasteurized-bacterium EVs | STING/IκB/NF-κB, cytokine, tight-junction and microbiota readouts; no inhibitor/knockout, and authors call for STING-deficient validation | Pasteurized-bacterium EVs produced broader protection than live-bacterium EVs | Comparative preparation evidence with an active comparator; pathway association is not STING dependence | Acute model; no dose response; possible soluble/bacterial contaminants; active components, chronic safety and human validation unresolved |
| DOI 10.1186/s12951-026-04866-5 [main-text Ref 81] | PBS; EcN alone; BEVs alone; EcN@BEVs; cellular protocatechualdehyde and infection-model single-cell/INVADE-seq analyses | Targeted metabolomics identified protocatechualdehyde, which recapitulated anti-M1 and ROS-associated effects in vitro. No protocatechualdehyde-depleted BEV or pathway-dependency experiment was reported; barrier, cytokine and microbiota findings are supportive. | The hybrid EcN@BEV product produced the broadest improvement in colitis, barrier, inflammatory and microbiota outcomes | Supports a live-biotherapeutic carrier/formulation effect and a candidate BEV metabolite; the active-component and molecular-mechanism claims remain non-causal | Complex live product; no dose response; methods/results dosing mismatch; BEV concentration and fuller purification not reported; no component-depleted control, containment/safety study or human validation |
| DOI 10.1016/j.apsb.2026.07.044 [main-text Ref 61] | Vehicle/model controls; 30/60 mg/kg PO-EVLPs; isolated RNA/protein/lipid fractions; miR159-y mimic/agomir, reporter and antagomir arms; acute, chronic and CAC models | Only the RNA fraction suppressed TGFBR2; mimic and wild-type/mutant luciferase assays supported direct targeting; agomir reproduced benefit and miR159-y antagomir significantly attenuated PO-EVLP protection in colitis. | Improved acute/chronic colitis and CAC outcomes and shifted ILC3/IL-22, NK-ILC1 and ILCreg-associated responses | Relatively strong preclinical cargo-dependency evidence for the miR159-y→TGFBR2/ILC axis, including loss-of-function attenuation | High oral mass dose; cross-kingdom miRNA quantification, delivery and human relevance require independent validation; systemic antagomir specificity and long-term safety remain unresolved |
| Tang et al. 2024; PMID 39583323 [main-text Ref 30] | Healthy control; DSS; unconditioned P-EVs; LPS pre-EVs | Conditioned-versus-unconditioned EV comparison, colon imaging, proteomics, 16S profiling, and PI3K/AKT readouts; no pathway inhibitor, cargo depletion, loss-of-function, or rescue experiment | LPS pre-EVs outperformed P-EVs for DAI, colon length, perianal findings, barrier markers, myeloid-state markers, microbiota diversity, and selected protein/pathway readouts | Supports donor-conditioning benefit and a PI3K/AKT association, not PI3K/AKT dependence or a defined cargo mechanism | Acute DSS model; dose amount not stated; active cargo and causal pathway unresolved; no chronic, human-mucosal, or long-term safety validation |
| Liang et al. 2023; PMID 37256205 [main-text Ref 95] | Control; DSS/TNBS plus PBS; hUC-MSC exosomes; Wnt-C59 or vehicle; TNF-alpha-injured organoids with or without exosomes | Wnt-C59 attenuated therapeutic, Lgr5, Ki-67, EdU, WNT2, and active beta-catenin readouts; this is pharmacologic pathway testing rather than lineage analysis | Improved disease, histology, permeability, Lgr5 expression, epithelial proliferation, and organoid growth | Supports a Wnt/beta-catenin-related repair branch with stem/progenitor-associated marker and organoid evidence; it does not establish lineage renewal | Acute models; no lineage tracing, stable epithelial differentiation, durable tissue architecture/function, defined active cargo, or long-term safety assessment |
| Feng et al. 2026; PMID 41773922 [main-text Ref 96] | TNBS controls; native iPSC-MSC exosomes; Exo-OE; Exo-KD; patient CD LPMCs | miR-34a-5p gain/loss experiments and PPP2R3A 3-prime-UTR targeting, with Wnt/beta-catenin and CD44 readouts | Improved colitis and Lgr5/Bmi1/Ki-67-associated repair readouts; Exo-OE enhanced and Exo-KD abolished the reported benefit; effects were followed for 7 days | Supports a preparation-specific miRNA-target/pathway branch with patient-cell relevance; it remains short-term marker-based regeneration evidence rather than lineage renewal | Acute TNBS model; 7-day follow-up; no lineage tracing, stable differentiation, durable tissue-function recovery, controlled clinical efficacy, or accessible full-text dose verification |

# Table S6. Registered therapeutic and ex vivo EV/exosome studies relevant to IBD

ClinicalTrials.gov API version 2.0.5, data timestamp 5 August 2026 at 09:00:06, was queried using IBD, ulcerative colitis, and Crohn disease condition modules combined with exosome and extracellular-vesicle intervention terms. After manual disease and intervention review, nine participant-treatment records were retained and one ex vivo human-relevance record was listed separately. Registration, recruitment, or completion status is not evidence of therapeutic efficacy.

| **Trial** | **Product/source** | **Phenotype** | **Route** | **Design/phase** | **Enrollment** | **Status** | **Results availability** |
| --- | --- | --- | --- | --- | --- | --- | --- |
| NCT04879810 | Ginger/plant exosomes with or without curcumin | Registry condition: 'Irritable Bowel Disease'; verify disease labeling | Oral daily for 28 days | Randomized, open-label, three-arm; phase not applicable | 4 actual | Completed | No registry-posted results; no efficacy inference |
| NCT05130983 | ExoFlo; bone-marrow MSC-derived EV product | Medically refractory CD | Intravenous; repeated dosing | Single-group, open-label phase 1 | 3 actual | Terminated | No registry-posted results; no linked peer-reviewed outcomes |
| NCT05176366 | ExoFlo; bone-marrow MSC-derived EV product | Medically refractory UC | Intravenous | Single-group, open-label phase 1 | 4 actual | Terminated | No registry-posted results; not evidence of efficacy |
| NCT05499156 | Placental MSC-derived exosomes | Perianal fistula in CD; registry text contains inconsistent Crohn/non-Crohn wording | Local fistula injection | Randomized, placebo-controlled, double-masked phase 1/2 | 80 actual (registry) | Unknown | No registry-posted results; status not verified since 2021 |
| NCT05836883 | ExoFlo; bone-marrow MSC-derived EV product | Perianal fistula in CD | Local fistula injection | Randomized, placebo-controlled, single-masked phase 1/2 | 5 actual | Terminated | No registry-posted results; no linked peer-reviewed outcomes |
| NCT06755021 | Whey-protein milk-derived exosome supplement | UC | Oral; 5 g or 10 g twice daily | Single-group, open-label; phase not applicable | 100 estimated | Not yet recruiting | No registry-posted results |
| NCT06853522 | Human umbilical-cord MSC exosomes | Active UC | Localized administration; exact procedure requires registry/protocol confirmation | Randomized, participant-masked early phase 1 | 40 estimated | Not yet recruiting | No registry-posted results |
| NCT06918808 | DB-3Q; bmMSC secretome proteins and EVs | Perianal fistulizing CD | Direct fistula injection, with one cohort also receiving IV dosing | Randomized, placebo-controlled, quadruple-masked phase 2 | 36 estimated | Recruiting | No registry-posted results |
| NCT07625293 | DB-3Q; bmMSC-derived EV-enriched secretome | Medically refractory luminal CD | Intravenous; dose-ranging | Randomized, placebo-controlled, double-masked phase 2 | 36 estimated | Not yet recruiting | No registry-posted results |
| NCT07721207 | hAMSC-derived secretome containing soluble factors and EVs | IBD patient biopsies, PBMCs, and organoid co-cultures | Ex vivo sample exposure; no participant dosing | Single-group ex vivo study; phase not applicable | 40 estimated | Not yet recruiting | Human-relevance study only; no registry-posted results |

**Registry interpretation note.** None of the ten retained records had registry-posted results or a registry-linked outcome publication at the data timestamp; an exact-NCT-identifier PubMed search also returned no indexed records. NCT07721207 exposes patient-derived samples ex vivo and is not a therapeutic participant-dosing study. NCT06568653 was excluded because its registered population is complex anal fistula without an established IBD phenotype. Biomarker, diagnostic, non-IBD, and false-positive intervention matches were also excluded after manual review.
